# Discovery of Two Novel Pyrazole Derivatives as Anticancer Agents Targeting Tubulin Polymerization and MAPK Signaling Pathways

**DOI:** 10.32604/or.2026.074945

**Published:** 2026-03-23

**Authors:** Denisse A. Gutierrez, Elisa Robles-Escajeda, Jose A. Lopez-Saenz, Robert A. Kirken, Edgar A. Borrego, Ana P. Betancourt, Soumya Nair, Sourav Roy, Armando Varela-Ramirez, Renato J. Aguilera

**Affiliations:** Border Biomedical Research Center and Department of Biological Sciences, The University of Texas at El Paso, El Paso, TX, USA

**Keywords:** Pyrazoles, cytotoxicity, triple-negative breast cancer (TNBC), apoptosis, tubulin polymerization inhibition, phosphorylation

## Abstract

**Objectives:**

Drug resistance is the major determinant of chemotherapy failure, leading to relapse and tumor progression, demonstrating the urgent need for novel antineoplastic drugs. This study aimed to evaluate the anticancer potential of two novel pyrazole derivatives, P3C.1 and P3C.2, and to elucidate their mechanism of action in cancer cells.

**Methods:**

The cytotoxicity of the compounds was evaluated across 27 different cancer cell lines via a nuclear staining assay. Subsequent flow cytometric and biochemical analyses were performed to assess reactive oxygen species (ROS) generation, apoptosis induction, mitochondrial integrity, and cell cycle progression. Additional studies included transcriptome analyses and immunoassays to characterize the molecular mechanisms underlying drug activity.

**Results:**

Two novel pyrazole derivatives, P3C.1 and P3C.2, were identified with potent cytotoxicity on a variety of cancer cell lines. Among the adherent cell lines tested, the triple-negative breast cancer (TNBC) cell line MDA-MB-231 exhibited the highest sensitivity to both compounds and was therefore selected for further experimentation. *In vitro* assays demonstrated that both compounds induced ROS generation, mitochondrial membrane depolarization, cell cycle arrest and apoptosis. Whole-transcriptome sequencing of P3C.1 and P3C.2-treated MDA-MB-231 and two lymphoblastic leukemia cell lines revealed four genes in common associated with cell signaling and membrane dynamics. Connectivity Map (CMAP) database comparisons of shared genes for each cancer subtype revealed a strong similarity between the two compounds with tubulin inhibitors, and subsequent assays confirmed that these compounds act as microtubule-disrupting agents. Moreover, protein phosphorylation analysis indicated that both compounds induced hyperphosphorylation of JNK, and ERK1/2, along with hypophosphorylation of p38 kinases.

**Conclusions:**

P3C.1 and P3C.2 emerged as promising anti-breast cancer agents with dual mechanisms of action involving microtubule disruption and altered kinase signaling, leading to induction of apoptosis.

## 1 Introduction

Cancer is the second leading cause of mortality worldwide, comprising a selection of hundreds of diseases denoted by uncontrolled cell growth and spread [1]. Breast cancer is the most prevalent cancer in women in the US, excluding skin cancer [2]. It has been estimated that today, approximately 1 in 43 women will die from breast cancer [3]. Interestingly, since 1989, the breast cancer death rate has decreased by 44% due to the development of new treatments and early detection [3]. Despite significant progress, tumor resistance to existing therapies remains one of the biggest challenges to overcome. Consequently, the continuous innovation, discovery, and advancement of new anticancer therapies and the identification of new anticancer drugs are of critical need.

Pyrazole derivatives are heterocyclic compounds with unique structures that possess extensive pharmacological activities, and importantly, they have been described as anticancer agents [4,5]. Their prominent anticancer potential is seen in many pyrazole-containing FDA-approved anticancer drugs. For example, niraparib, crizotinib, ruxolitinib, avapritinib, and ibrutinib are indicated for use in ovarian, lung, blood and bone marrow, and Leukemia/lymphoma types of cancer, respectively [4,6–10]. These drugs have diverse mechanisms of action; some are distinguished for their kinase-inhibitory activity, such as ibrutinib, ruxolitinib, and crizotinib, which target Bruton’s Tyrosine Kinase (BTK), JAK1/2, and ALK kinases, respectively [4,6,7]. Interestingly, a recent publication described pyrazole derivatives as antiangiogenic agents affecting Ca^2+^ mobilization and cytoskeletal organization [11]. Moreover, pyrazoles have also been described as tubulin polymerization inhibitors [4,12]. Tubulin polymerization is indispensable during cell proliferation, signaling, trafficking, and migration; thus, its inhibition has become a crucial target for anticancer drug design and discovery. Currently, several microtubule-targeting agents (MTA’s) are widely used in clinics for cancer therapy. For example, vinblastine, vincristine, vinorelbine, and vindesine are classified as vinca alkaloids [4,13,14].

The present study aimed to examine the antitumor activity of two novel pyrazole derivatives, P3C.1 and P3C.2, and to determine their mode of action in cancer cells.

## 2 Materials and Methods

### 2.1 Cell Lines and Culture Conditions

All the cell lines used in this study were found to be mycoplasma negative and were consistently cultured at 37°C with a 5% CO_2_ humidified atmosphere. The following cell lines were grown in Dulbecco’s Modified Eagle’s Medium (DMEM) culture medium (Corning, MT10013CV, NY, USA) supplemented with 100 U/mL of penicillin, 100 μg/mL of streptomycin (Corning, 30001138) and 10% fetal bovine serum (FBS) (Seraprime, F117-13, CO, USA): MDA-MB-231 (ATCC, HTB-26, VA, USA) MDA-MB-231 LM2-4 [15], MDA-MB-468 (ATCC, HTB-132), MCF-7 (ATCC, HTB-22), PANC-1 (ATCC, CRL-1469), A375 (ATCC, CRL-1619), HEPG2 (ATCC, HB-8065), HeLa (ATCC, CCL-2) and HS-27 (ATCC, CRL-1634). Additionally, the MCF-7 cells were supplemented with 10 μg/mL of insulin (Sigma-Aldrich, *I1882*, MO, USA). In addition, the RAMOS (ATCC, CRL-1596), CEM (ATCC, CCL-119), JURKAT (ATCC, TIB-152), NALM-6 (ATCC, CRL-3273), RPMI-8226, MM.1S, MM.1R, U266, Rec-1, JVM-13, A549 (ATCC, CRM-CCL-185), HCC70 (ATCC, CRL-2315), HCC1419 (ATCC, CRL-2326), T47D (ATCC, CRL-2865), KMS-11, and OVCAR-5 cell lines were grown in Roswell Park Memorial Institute-1640 (RPMI-1640) culture media (Cytiva-Hyclone, SH30027FS, UT, USA,) supplemented with 10% FBS, 100 U/mL of penicillin, and 100 μg/mL of streptomycin. Similarly, the MiNo (ATCC, CRL-3000), Jeko-1 (ATCC, CRL-3006), and HL-60 (ATCC, CCL-240), cell lines were grown in RPMI culture medium (Cytiva-Hyclone, SH30027FS) but supplemented with 15% FBS for MiNo and 20% for Jeko-1 and HL-60. Lastly, PC-3 (ATCC, CRL-1435), and MCF10A (ATCC, CRL-10317), cell lines were grown in Dulbecco’s Modified Eagle’s Medium-F12 (DMEM-F12) media (Corning, MT10090CV) with 10% FBS, 100 U/mL of penicillin, and 100 µg/mL of streptomycin. Additionally, for the MCF10A cells, the media was supplemented with 0.5 µg/mL of hydrocortisone (Sigma-Aldrich, H0888-1G), 20 ng/mL of epidermal growth factor (PeproTech, AF-100-15, NJ, USA), and 10 µg/mL of insulin. Unless otherwise specified (see Acknowledgement section), all cell lines were purchased from and authenticated by the American Type Culture Collection.

### 2.2 Pyrazole Derivatives and Other Drugs Used

The P3C.1 compound was initially acquired from Sigma-Aldrich (R495808) and, subsequently, from AKOS GmbH (AKOS024343488, Lörrach, Germany). The P3C.2 compound was purchased from ChemBridge Corporation (ID# 5784143, CA, USA,). The following drugs were acquired from Fisher Scientific: vinblastine (Fisher Scientific, AAJ63598MA, MA, USA), etoposide (Calbiochem, 34120525MG, CA, USA), and paclitaxel (Fisher Scientific, AC328420050). Lyophilized compounds were dissolved in Dimethyl sulfoxide (DMSO) (Fisher Scientific, PI20688) at a concentration of 10 mM (stock solutions) and stored at −20°C.

### 2.3 Cytotoxic Evaluation through the DNS Assay

To evaluate the cytotoxicity of experimental compounds, the Differential Nuclear Staining (DNS) assay was performed [5]. This assay allows the quantification of live and dead cells by using two nuclear dyes and a bioimaging system (ImageXpress Pico, Molecular Devices, San Jose, CA). Hoechst, permeable to all cells, facilitates the quantification of a whole cell population, and Propidium Iodide (PI), which is only permeable to cells with compromised plasma membranes, enables the quantification of dead cells. Treated and stained cells are imaged with an automated imaging system that ultimately provides a percentage of live and dead cells. For this assay, ten thousand cells were seeded in 100 µL of culture medium per well in 96-well plates (Greiner Bio-one CELLSTAR, Kremsmünster, Austria, 07-000-162) and incubated overnight at optimal conditions (37°C, in a 5% CO_2_ humidified atmosphere). Cells were then treated with increasing concentrations of the drugs (P3C.1 and P3C.2; 0.01, 0.05, 0.1, 0.5, 1, 2.5, 5, and 10 µM) and incubated for either 48 or 72 h. Non-adherent cell lines derived from cells growing in suspension, such as Ramos, CEM, HL-60, Jurkat, Nalm-6, RPMI-8226, MM.1S, MM.1R, KMS II, U266, Rec-1, MiNo, Jeko-1, and JVM-13, were incubated with the drugs for 48 h. In contrast, adherent cells such as: MDA-MB-231, MDA-MB-231 LM2-4, MDA-MB-468, HCC70, HCC1419, MCF-7, T47D, OVCAR-5, A549, PC-3, PANC-1, HEPG2, and A375 were incubated with the drugs for 72 h. In addition, DMSO (solvent), Hydrogen peroxide (H_2_O_2_), 1 mM final concentration, (Fisher Scientific, H325-500), and Untreated (UNT) samples were included as controls. Two hours before the incubation time ended, a staining mixture of Hoechst 33342 (Invitrogen, H21492, OR, USA) and PI (MP Biomedicals, ICN19545810, OH, USA) at a final concentration of 5 µg/mL each was added to each well in the dark and incubated for 2 h at optimal conditions [16]. Afterwards, cell imaging was performed using the ImageXpress Pico bioimaging system (Molecular Devices, San Jose, CA). To image the entire well area, a 2 × 2 montage for each fluorescent channel was used using a 4× objective. In this strategy, the Hoechst dye (blue channel) stains all cells’ nuclei, which is used to quantify the total cell population. Moreover, the propidium iodide dye stains only cells with permeable plasma membranes, used to identify dead cells (red channel). Percentages of cell death were obtained using the Cell Reporter Xpress software (Molecular Devices, San Jose, CA) and were used to calculate CC_50_ values. At least three technical replicates were included per treatment in one assay performed.

### 2.4 CC_50_s (Cytotoxic Concentration 50%) and SCI (Selective Cytotoxicity Index) Values Calculation

The CC_50_ value is the cytotoxic concentration of a given drug necessary to kill 50% of a cell population. These values were obtained by using a linear interpolation equation (https://www.johndcook.com/interpolator.html). Selective cytotoxicity index values denote selectivity of a given drug for killing cancer cells without inducing substantial damage to non-cancerous cells [17]. These values were calculated by dividing the CC_50_ values of a non-cancerous cell line (MCF10A for the adherent cell mentioned in Section 2.1, and HS27 for the non-adherent cells also mentioned in Section 2.1) by the CC_50_ of the cancer cell line [5].

### 2.5 Apoptosis Induction Analysis

To evaluate the ability of the compounds to induce apoptosis, the distribution of phosphatidylserine (PS) in cell membranes was assessed using the Annexin V-FITC and PI assay kit (Beckman Coulter, IM3546, PN, USA). The assay was performed according to the manufacturer’s instructions. On day one, MDA-MB-231 cells were seeded in 24-well plates (Corning, 07-200-84, NY, USA) at a density of 1 × 10^5^ cells per well in 1 mL of culture medium and incubated overnight (ON) under optimal conditions. The next day, treatments were added to cells in triplicate as follows: CC_50_ and 2× CC_50_ concentrations calculated at 24 h for each compound were used for experimental treatments (P3C.1 and P3C.2). DMSO (solvent), untreated, and 1 mM Hydrogen peroxide H_2_O_2_, (an apoptosis inducer) were included as controls. Treated cells were incubated for 24 h (optimal conditions) and subsequently trypsinized and harvested into flow cytometry tubes. Then, cells were centrifuged (262× *g*) for 5 min at room temperature, and supernatants were decanted. Cell pellets were resuspended in 100 μL of 1X binding buffer, containing Annexin V-FITC and PI, and then incubated on ice in the dark for 30 min. Afterward, 400 μL of ice-cold 1× binding buffer was added to each sample, and readings were taken immediately in a GALLIOS Flow cytometer (Beckman Coulter, Miami, FL, USA). The FL1 and FL2 detectors were used to identify positive cells for FITC (green fluorescence) and PI (red fluorescence), respectively. At least 10,000 events (cells) were collected per sample. Cells emitting only a green, fluorescent signal were identified as early apoptotic, while cells with both green and red fluorescence signals were identified as late apoptotic. Also, cells positive only for PI or red fluorescent signal were categorized as necrotic. Data collection and analysis were performed using the 1.3 Kaluza software (Beckman Coulter). To exclude debris and doublets, forward scatter (FSC) vs. side scatter (SSC) dot plots were created, and gates were drawn around the main cell population, excluding events with very low FSC and SSC corresponding to debris. In addition, events with very high FSC and SSC, indicative of aggregates or doublets, were excluded, and only gated populations were used for subsequent analyses. To define apoptotic and necrotic populations, two-parameter dot plots of FL1 (*x*-axis) vs. FL2 (*y*-axis) were generated, and quadrant gating was performed. Cells positive only for FL1, corresponding to the early apoptotic cells, were identified on the bottom right quadrant. Late apoptotic cells, positive for FL1 and FL2, were located in the upper right quadrant. Necrotic cells, positive only for FL2, were detected on the upper left quadrant.

### 2.6 Mitochondrial Health Assay

To determine whether the experimental drugs induce mitochondrial damage, the mitochondrial membrane potential was assessed in MDA-MB-231 cells exposed to P3C.1 and P3C.2. The MitoProbe JC-1 assay kit (Molecular Probes, M341520, Eugene, OR, USA) was used according to the manufacturer’s instructions. Cells were seeded at a concentration of 100,000 cells per well in 1 mL of culture media in 24-well plates and incubated overnight at optimal conditions. Then, the cells were treated with experimental and control samples as described in Section 2.5. Cells were incubated with the treatments at optimal conditions for 5 h. Subsequently, culture media containing floating cells were collected from each well, and cells were trypsinized for 5 min or until detached. Both floating and trypsinized cells were carefully harvested in flow cytometry tubes (VWR, 60818433, Radnor, PA, USA). Samples were spun down for 5 min (262× *g*), supernatants decanted, and pellets washed with 1 mL of warmed phosphate-buffered saline (PBS; Cytiva-Hyclone, SH30027FS, Logan, UT, USA). Cells were spun down (5 min, 262× *g*), supernatants decanted, and pellets resuspended in 500 μL of pre-warmed PBS. After that, 5 μL of the JC-1 dye (final concentration, 2 μM) was added to each sample. Each tube was vortexed for 5 s, and cells were incubated for 30 min at 37°C with 5% CO_2_. After the incubation period, 2 mL of warmed PBS was added to each tube and spun down for 5 min at 262× *g*. Next, the supernatants were decanted, and the cells were resuspended in 500 μL of PBS. Measurements were immediately taken by flow cytometry (10,000 events per sample) using the FL1 (green; JC-1 monomers) and FL2 (red; JC-1 aggregates) detectors. A decrease in the red to green fluorescence ratio was used as an indicator of mitochondrial depolarization. Cells emitting only a green fluorescence represent cells with loss of mitochondrial membrane potential, reflecting a shift from JC-1 red aggregates to green monomers. Data acquisition and analysis were accomplished using the Kaluza 1.3 software. At least three technical replicates were included per treatment in one biological assay.

### 2.7 Evaluation of ROS Induction

To monitor the accumulation of ROS in cells treated with the experimental compounds, the Carboxy-H_2_ DCFDA indicator (Fisher Scientific, C400) was used. MDA-MB-231 cells were seeded in 24-well plates at a density of 100,000 cells per well in 1 mL of culture medium and were incubated overnight to promote cell attachment under optimal conditions. The next day, cells were treated with CC_50_ and 2× CC_50_ concentrations calculated at 24 h for each compound (P3C.1 and P3C.2). Untreated, DMSO (solvent), 1 mM H_2_O_2_, and 10 μM Rotenone (Calbiochem, 557368), a well-known ROS inducer, were included as positive controls. Cells were incubated with the drugs for 18 h and were subsequently trypsinized and harvested in flow cytometry tubes. Cells were spun down (262× *g* for 5 min), supernatant decanted, and cells resuspended in 1 mL of PBS containing the Carboxy-H_2_ DCFDA indicator (10 μM final concentration). Samples were incubated for 1 h at 37°C and then centrifuged (262× *g* for 5 min) to remove the dye. Cell pellets were resuspended in 500 μL of pre-warmed PBS, and the samples were returned to 37°C for a recovery time of 20 min. After that, measurements were immediately taken via flow cytometry. Only cells with accumulated ROS in the presence of the indicator emit a green-fluorescent signal as a result of the acetate groups being removed from the indicator by intracellular esterases and oxidation. The FL1 detector was used to identify green, fluorescent cells. A total of 100,000 events were quantified per sample. Data acquisition and analysis were achieved as described above. At least three technical replicates were included per treatment in one biological assay.

### 2.8 Cell Cycle Progression Analysis

Quantification of DNA content was performed to evaluate the cell cycle phase distribution on MDA-MB-231 and Jurkat cells treated with P3C.1 and P3C.2 [5]. A total of 100,000 cells were seeded in 24-well plates, with 1 mL of culture media per well. Cells were incubated at optimal conditions, and the next day, they were treated in triplicate with CC_12.5_, CC_25_ and CC_50_ concentrations of P3C.1 and P3C.2. To reduce large amounts of cell death to facilitate the detection of cell cycle arrest, lower concentrations of the drugs were used (CC_12.5_ and CC_25_). CC_50_ values were used to calculate the CC_12.5_ and CC_25_ concentrations. Untreated, DMSO (solvent), and 100 μM Etoposide were incorporated as controls. The treated cells were returned to the incubator for 72 h. Afterwards, cells were harvested in flow cytometric tubes and centrifuged (262× *g*, 5 min). Cell pellets were resuspended in 500 μL of an isotonic Nuclear Isolation Media containing DAPI (NIM-DAPI, Merk KGaA, 28718-90-3, Darmstadt, Germany) and were immediately analyzed by flow cytometry. The Nuclear Isolation Media consisted of 1x PBS supplemented with calcium and magnesium (0.01 M phosphate buffer saline, 0.1 mM CaCl_2_, 0.5 mM MgSO_4_·7 H_2_O), 0.6% of NP-40, 0.2% Bovine serum albumin (BSA; Sigma-Aldrich, A2153-100G), and 10 μg/mL of DAPI [18]. The 405 nm laser and FL9 detector were used to measure the amount of DAPI bound to DNA in individual cells. For this assay, at least 50,000 events (cells) were collected to obtain a more precise phase distribution profile. The Kaluza 1.3 software was used to collect and analyze data.

### 2.9 Next-Generation RNA Sequencing (RNA-Seq)

For RNA isolation, 1 × 10^6^ cells (MDA-MB-231, Jurkat, and CEM) were seeded in T-25 flasks in 5 mL of culture medium and incubated overnight at 37°C, 5% CO_2_. The next day, cells were treated in triplicate with 2× CC_50_ concentrations calculated at 24 h, for P3C.1 and P3C.2, including the solvent control (0.01% DMSO) for each cell line. Cells were exposed to treatments for 6 h, and then they were harvested in 15 mL conical tubes, centrifuged (262× *g*, 5 min), and pellets were washed with 1 mL of PBS. Cell pellets were formed again by centrifugation, and RNA extraction was performed using the RNeasy Mini Kit (Qiagen, *74104*, Hilden, Germany) according to the manufacturer’s instructions. Before library preparation, RNA quantification was assessed using an RNA BR Assay Kit for Qubit 3.0 (Invitrogen, *Q10210*, Carlsbad, CA, USA). RNA integrity was also evaluated in all samples using an RNA Screen Tape (Agilent, 5067-5576, Santa Clara, CA, USA) in a 4200 TapeStation (Agilent, G2991BA). Only samples with a RIN greater than 7.0 were considered for further experimentation. To transform the mRNA into a cDNA library, the TruSeq Stranded mRNA Library Kit (Illumina, 20020594, San Diego, CA, USA) was used. Subsequently, the cDNA was sequenced via the NextSeq 1000/2000 P2 XLEAP-SBS reagent kit (300 cycles) following the manufacturer’s instructions (Illumina, 20100985) and using the NextSeq 2000 system (Illumina).

### 2.10 RNA-Seq Analysis

The mRNA sequencing data were subjected to quality control using FastQC and the ShortRead package, followed by trimming with Trimmomatic to remove low-quality bases and adapter sequences. The processed reads were aligned to the Human reference genome (NCBI GRCh38) using Tophat2 with default parameters. Gene-level expression was quantified using Cufflinks. Differential gene expression analysis was performed with DESeq2, R package, comparing treatment samples to their respective DMSO-treated controls. Genes exhibiting a log2 fold change value ≥1.0 and ≤−1.0 and a *p*-adjusted value (False Discovery Rate; FDR) ≤ 0.05 were considered significantly differentially expressed and were selected for downstream analyses. The differentially expressed genes (DEGs) were further processed to obtain z-score-standardized expression values (gene-wise normalization), which were used to generate a heatmap using pheatmap (Version 1.0.13), R package. Hierarchical clustering of the genes was performed using Euclidean distance with complete linkage to illustrate the expression patterns for each experimental condition.

### 2.11 Ingenuity Pathway Analysis (IPA)

Ingenuity Pathway Analysis (IPA, Qiagen, Redwood City, CA, version 153384343) [19]. Core analysis was performed using the Ingenuity knowledge base as the reference set to identify the gene-enriched canonical pathways affected by P3C.1 and P3C.2 in MDA-MB-231, Jurkat, and CEM cells. DEGs common between the three cell lines and the two compounds, as well as those common for P3C.1 and P3C.2 in MDA-MB-231, were analyzed. Additionally, genes shared by P3C.1 and P3C.2 in Jurkat and CEM were also used for this analysis. IPA employs Fisher’s exact test to determine the significance of the gene-enriched pathway represented as −log10(*p*-value). The default maximum and minimum values set by IPA were used. The figures were finalized using the “Path Designer” tool available in IPA, which provides more clarity in differentiating the connections among the different types of molecules, and were downloaded directly from the IPA network visualization tool.

### 2.12 CMap Analysis

The Connectivity Map from the Broad Institute library database was used (https://clue.io/query) to compare the expression signatures of P3C.1 and P3C.2 with those of other known drugs included in this database. Genes considered significantly differentially expressed (log_2_ fold change value ≥1.0 and ≤−1.0 and a *p*-adjusted value (FDR) ≤ 0.05) that were induced in common by P3C.1 and P3C.2 in breast cancer cells (MDA-MB-231) and in leukemic cell lines (Jurkat and CEM) were used as input to query CMap for similar perturbagen signatures. The following query parameters were selected: for data category, gene expression L1000 (the largest dataset available), also dataset 1.0, which refers to the pilot Connectivity Map dataset, and individual query, to run a single gene expression signature at a time. The output data were arranged by compound and perturbagen class member and were ranked according to a CMap connectivity score (tau), which represents the similarity of the differentially expressed gene sets, and the query gene set list provided. The magnitude of the score indicates the degree of similarity (positive scores) or dissimilarity (negative scores), considering the range of −100 to 100 as cited in the CMap Broad Institute website: https://clue.io/connectopedia/connectivity_scores. Due to the small number of genes found in common for P3C.1 and P3C.2 in the three analyzed cell lines (MDA-MB-231, Jurkat, and CEM), the software was unable to perform a connectivity map analysis on this subset of genes (requires at least 10 genes as input).

### 2.13 Microtubule Structure Analysis

The morphology of the cytoskeletal protein tubulin (microtubules) was evaluated in MDA-MB-231 cells treated with P3C.1 and P3C.2 via confocal microscopy [20]. Cells were seeded at a density of 2500 cells per well in 100 μL of culture media in 96-well imaging thin-bottom microplates (BD Falcon, Franklin Lakes, NJ, USA, *353216*) and incubated overnight at optimal conditions. The next day, cells were treated with CC_50_ concentrations of P3C.1 and P3C.2, DMSO (the solvent control), vinblastine (3 μM), and untreated cells were included as controls. Cells were exposed to treatments for 2 h and were subsequently fixed, permeabilized, and stained as follows: culture media was removed from cells, and 100 μL of freshly prepared 4% formaldehyde (VWR, *VW3408-1*) was added, and cells were incubated at room temperature for 20 min. Then, formaldehyde was removed, and the cells were washed twice with PBS. They were then permeabilized with 200 μL of washing buffer (0.1% Tween 20, in PBS; Acros Organics, Geel, Belgium, *9005-64-5*). Afterward, the washing buffer was removed, and the cells were treated with 200 μL of a blocking solution (2% BSA; Sigma-Aldrich, *A2153-100G* in PBS) for 1 h. Plates were left on a rocking platform at room temperature. Then, the blocking solution was removed, and cells were stained with 50 μL per well of the following mixture: 1 μL of anti α-tubulin monoclonal antibody conjugated to Alexa Fluor 488 (Fisher Scientific, 16-232-MI, clone DM1A, 1:50 dilution), and 0.5 μL of DAPI (1 mg/mL; KGaA, Merk, 28718-90-3) in 48.5 μL of PBS. Cells were incubated with the staining solution as prepared above on a rocking platform at 4°C overnight. The next day, the staining solution was removed, and cells were washed three consecutive times with 200 μL of PBS, and 200 μL of this buffer was left in each well. Subsequently, cell imaging was achieved by using a laser scanning confocal microscope (LSM-700, Zeiss, Oberkochen, Germany) and a 40× objective (EC Plan-Neofluar). Images were acquired and analyzed using the Zen 2009 6.0 software (Zeiss).

### 2.14 Analysis of Mitotic Spindle Formation

Analyses of the microtubule spindle formation were performed in HeLa cells undergoing active cell division after 2 h of exposure to P3C.1 and P3C.2 by confocal microscopy. Cell preparations, treatments, and staining were performed as described in Section 2.13. In this assay, 2 drops (approximately 100 μL from a dropper bottle) of phalloidin conjugated to Alexa Fluor-568 (Fisher Scientific, R37112) per 1 mL were also added to the staining mixture.

### 2.15 Tubulin Polymerization Analysis

To evaluate the ability of P3C.1 and P3C.2 to inhibit tubulin polymerization, the tubulin polymerization assay kit (Cytoskeleton, BK006P, Denver, CO, USA) was utilized following the manufacturer’s instructions. This method is based on the work of Shelanski et al., who stated that microtubules scatter light to an extent that is proportional to the concentration of microtubule polymer. The rate of tubulin polymerization was assessed every minute over a period of 1 h using a spectrophotometer (SpectraMax 250; Molecular Devices, San Jose, CA, USA) in a kinetic mode. Before starting the procedure, a half area 96-well plate (provided with the kit) was incubated at 37°C for 15 min. Briefly, 9 μL of pre-warmed general tubulin buffer was added to each well, followed by the addition of 1 μL of P3C.1 (10 μM), and P3C.2 (10 μM), vinblastine (5 μM; positive control for tubulin polymerization inhibition), paclitaxel (PTX; 10 μM, tubulin stabilizing control), and DMSO (0.009%, solvent control) into individual wells in duplicate and incubated at 37°C for 5 min. Concentrations shown above denote final concentrations per treatment. During the incubation time, tubulin powder was reconstituted with 1.1 mL of ice-cold general tubulin buffer previously supplemented with GTP and was kept on ice until used. For every 6 samples, tubulin polymerization (TP) buffer was prepared as follows: 750 μL of general tubulin buffer, 250 μL of tubulin glycerol buffer, and 10 μL of GTP stock (100 mM). See the manual for more details. The TP buffer was kept on ice. Next, 200 μL of the reconstituted tubulin was combined with 420 μL of TP buffer. Immediately after, 100 μL of this mixture was added to each sample in the half-area 96-well plate, and measures were taken at an absorbance of 340 nm in a 37°C environment using the SoftMax Pro software version 5.4.1 (Molecular Devices).

### 2.16 Phosphorylation Analysis of MAPK Pathways

To evaluate the levels of phosphorylation/activation of key signaling pathways induced by P3C.1 and P3C.2 in MDA-MB-231 cells, the Milliplex Multi-Pathway 9-plex Magnetic Bead kit, phosphoprotein (Millipore, Burlington, MA, USA, *48-680MAG*), and the Luminex LX-100 platform were used. To prepare cell lysates, 5 × 10^6^ of MDA-MB-231 cells were collected in 15 mL conical tubes and centrifuged for 5 min at 262× *g*. Supernatants were carefully removed with a pipette without disturbing the pellets, and 500 μL of pre-warmed culture media were added to each sample and transferred to 1.5 mL microcentrifuge tubes. Experimental treatments and solvent control were performed in duplicate for each tube, resulting in a final concentration of 10 μM for P3C.1 and P3C.2 and 0.2% for DMSO. Cells were incubated with the treatments for 3 h at 37°C. Tubes were covered with parafilm to prevent evaporation during the incubation period and were gently agitated (every 15 min) to ensure cell-treatment contact. After the incubation period, the cells were centrifuged (5 min at 262× *g*) and the supernatants were carefully removed with a pipette. Then, the cells were washed once with 1 mL of PBS, centrifuged again, and the supernatant was removed carefully. Afterwards, 50 μL of 1X Milliplex lysis buffer with freshly added protease inhibitors (1x HALT; Fisher Scientific, *78439*, and 1 μM PMSF; Acros Organics, *329-98-6*) was added to each sample. The pellets were disturbed by repeatedly flicking the tube until they were completely dissolved. Cell Lysates were then incubated at 4°C under rotation for 1.5 h. Next, the samples were centrifuged at 14,000 rpm for 15 min at 4°C, and supernatants were carefully transferred to new microcentrifuge tubes. Samples were stored immediately at −70°C until used. Protein quantification was assessed by using the NanoDrop Spectrophotometer (protein/280). A total of 25 μg of protein cell lysate was used per sample for the immunoassay protocol following the manufacturer’s instructions. The following analytes were assessed: ERK/MAP kinase 1/2 (Thr185/Tyr187), JNK (Thr183/Tyr185), and p38 (Thr180/Tyr182). Data acquisition and analysis were achieved using the xPONENT 3.1 software (Luminex, Austin, TX, USA).

### 2.17 Statistical Analyses

The statistical significance of the different experiments presented in this study was calculated using two-tailed Student’s *t-*tests (Social Science Statistics *t*-test calculator, 10 March 2025, retrieved from [https://www.socscistatistics.com/tests/studentttest/default2.aspx]). *p*-values represent a comparison of experimental samples against the solvent control (DMSO). Significant *p*-values are represented with asterisks as follows: *p < 0.05, **p < 0.01, and ***p < 0.001. To ensure the reliability and validity of the results, each experiment was conducted with at least three independent measurements, unless otherwise specified. Results are presented as averages and their corresponding standard deviations.

## 3 Results

### 3.1 P3C.1 and P3C.2 Compounds Are Highly Cytotoxic to Multiple Human Cancer Cell Lines

**Figure 1:**
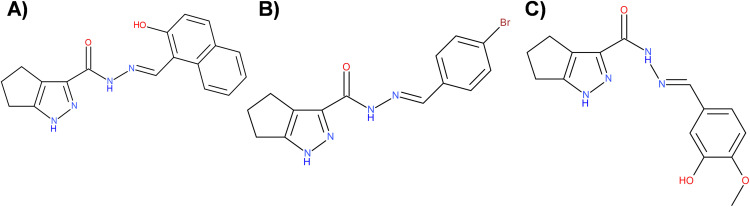
Chemical structure of three pyrazole derivatives. (**A**) P3C; N^′^-[(2-hydroxy-1-naphthyl)methylene]-1,4,5,6-tetrahydrocyclopenta [c]pyrazole-3-carbohydrazide, (**B**) P3C.1; N^′^-(4-bromobenzylidene)-1,4,5,6-tetrahydrocyclopenta [c]pyrazole-3-carbohydrazide and (**C**) P3C.2; N^′^-(3-hydroxy-4-methoxybenzylidene)-1,4,5,6-tetrahydrocyclopenta [c]pyrazole-3-carbohydrazide. Figure was created using BIOVIA Draw 2020 EE (Dassault Systèmes, BIOVIA, San Diego, CA, USA).

**Figure 2:**
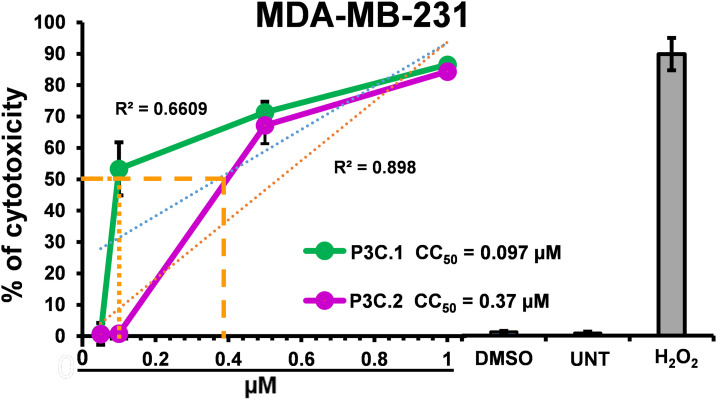
Cell-death curves of P3C.1 and P3C.2 on MDA-MB-231 cells treated for 72 h. CC_50_ values were calculated using a concentration gradient of the experimental compounds utilizing the DNS assay. Control treatments are depicted in the bar graphs: DMSO, UNT, and H_2_O_2_. Linear regression analysis is shown for each compound: P3C.1 (blue dots; R_2_ = 0.6609) and P3C.2 (orange dots; R_2_ = 0.898).

This study focused on two pyrazole derivatives, P3C.1 and P3C.2 (Fig. 1), identified by their structural similarity to P3C, a previously described anticancer pyrazole [5]. The cytotoxicity of P3C.1 and P3C.2 was evaluated via the DNS assay, and CC_50_ values were calculated by linear interpolation (see Section 2). Both compounds exhibited high cytotoxicity against 27 different cancer cell lines tested (Table 1) with CC_50_ values ranging from 0.07 to >40 µM. The triple-negative breast cancer cell line MDA-MB-231 was the most sensitive to the two compounds in adherent cells (Table 1; top part and Fig. 2) with CC_50_ values of 0.097 and 0.37 µM for P3C.1 and P3C.2, respectively. Likewise, the mantle cell lymphoma MiNo cell line was the most sensitive to both compounds in non-adherent tumor cells (Table 1; bottom part) with CC_50_ values of 0.29 and 0.077 µM to P3C.1 and P3C.2, respectively. In contrast, the T47D (breast carcinoma) and U266 (myeloma) cell lines were the most resistant to both compounds, in adherent and non-adherent tumor cells, respectively, with CC_50_ values of 8.11, 7.65, and >40, >20 for P3C.1 and P3C.2, respectively. Overall, most of the cell lines tested displayed CC_50_ values in the nanomolar range (<1 µM), demonstrating the widespread cytotoxic activity of P3C.1 and P3C.2 towards cancer cell lines from diverse tissue origins.

**Table 1:** CC_50_ values were calculated at 48 h (for non-adherent cells) and 72 h (for adherent cells) of exposure to P3C.1 and P3C.2 in 27 different human cancer cell lines and two non-cancerous cell lines.

	Cell Line	Origin	P3C.1 CC_50_ (µM) †	SCI ‡	P3C.2 CC_50_ (µM)	SCI
	MCF10A	Normal breast epithelial tissue	0.42 ± 0.03	1.00	0.36 ± 0.012	1.00
	MDA-MB-231	Breast adenocarcinoma	0.097 ± 0.007	4.33	0.37 ± 0.03	0.97
	MDA-MB-231 LM2-4	Lung metastatic breast adenocarcinoma	0.14 ± 0.042	3.00	0.42 ± 0.01	0.86
**Cytotoxicity adherent cell lines(72 h)**	MDA-MB-468	Breast adenocarcinoma	0.098 ± 0.009	4.29	4.51 ± 0.26	0.08
	HCC70	Breast carcinoma	2.01 ± 0.42	0.21	16.80 ± 1.98	0.02
	HCC1419	Breast carcinoma	3.7 ± 0.45	0.11	>20	0.02
	MCF-7	Breast adenocarcinoma	0.81 ± 0.13	0.52	1.39 ± 0.23	0.26
	T47D	Breast carcinoma	8.11 ± 0.50	0.05	7.65 ± 0.81	0.05
	OVCAR-5	Ovarian carcinoma	0.36 ± 0.003	1.17	2.74 ± 0.20	0.13
	A549	Lung carcinoma	0.18 ± 0.02	2.33	18.37 ± 1.23	0.02
	PC-3	Prostatic adenocarcinoma	0.43 ± 0.01	0.98	0.30 ± 0.051	1.20
	PANC-1	Pancreatic carcinoma	0.62 ± 0.13	0.68	0.65 ± 0.10	0.55
	HEPG2	Liver carcinoma	0.82 ± 0.12	0.51	11.53 ± 0.38	0.03
	A375	Melanoma	0.082 ± 0.001	5.12	0.62 ± 0.057	0.58
	Hs27	Normal foreskin fibroblast	0.44 ± 0.018	1.00	0.76 ± 0.015	1.00
	Ramos	Burkitt’s lymphoma	0.32 ± 0.01	1.38	0.28 ± 0.023	2.71
	CEM	Acute lymphoblastic leukemia	0.07 ± 0.01	6.29	0.44 ± 0.011	1.73
**Cytotoxicity non-adherent cell lines (48 h)**	HL-60	Acute promyelocytic leukemia	0.42 ± 0.01	1.05	0.19 ± 0.07	4.00
	Jurkat	Acute T cell leukemia	0.39 ± 0.04	1.13	0.26 ± 0.044	2.92
	Nalm-6	Acute lymphoblastic leukemia	0.48 ± 0.09	0.92	0.26 ± 0.134	2.92
	RPMI-8226	Plasmacytoma	0.07 ± 0.001	6.29	0.39 ± 0.023	1.95
	MM.1S	Myeloma	0.75 ± 0.01	0.59	0.42 ± 0.007	1.81
	MM.1R	Multiple myeloma	0.19 ± 0.03	2.32	0.950 ± 0.03	0.80
	KMS II	Myeloma	4.03 ± 0.67	0.11	0.44 ± 0.030	1.73
	U266	Myeloma	>40	0.01	>20	0.04
	Rec-1	Mantle cell lymphoma	0.107 ± 0.006	4.11	>20	0.04
	MiNo	Mantle cell lymphoma	0.29 ± 0.03	1.52	0.077 ± 0.007	9.87
	Jeko-1	Peripheral blood lymphoma	0.18 ± 0.05	2.44	0.080 ± 0.01	9.50
	JVM-13	B Prolymphocytic Leukemia	0.94 ± 0.017	0.47	0.35 ± 0.0078	2.17

Note: **†** CC_50_ values indicate the cytotoxic concentration at which P3C.1 and/or P3C.2 kill 50% of the cell population. Standard deviations are represented by ±. **‡** Selective cytotoxicity index (SCI) values of adherent and suspension cells were calculated by dividing the MCF10A (for adherent cell lines) and Hs27 (for non-adherent cells) CC_50_ values by the CC_50_ of each cancer cell line.

In addition, to evaluate the compound’s potential cancer-type selectivity, two non-cancerous cell lines, MCF10A (breast epithelial) and Hs27 (foreskin fibroblast), were also assessed for cytotoxicity with the two compounds, and SCI (selective cytotoxicity index) values were calculated. As shown in Table 1, the highest selective cytotoxicity index values were observed for P3C.1 in MDA-MB-231 (SCI = 4.33), MDA-MB-468 (SCI = 4.29), A375 (SCI = 5.12), CEM (SCI = 6.29), and RPMI-8226 (SCI = 6.29). Additionally, for P3C.2, HL-60, MiNo, and Jeko-1 cells, the highest SCI values were observed at 4.00, 9.87, 9.50, respectively. The observed variability in the CC_50_s and SCI values suggests a heterogeneous pattern of cancer type selectivity among the compounds studied. The MDA-MB-231 cell line was chosen for further experimentation due to its sensitivity to P3C.1 and P3C.2, as exhibited above (Table 1), and for comparison purposes with the previously reported P3C anti-breast cancer drug [5].

### 3.2 P3C.1 and P3C.2 Induce Apoptosis via the Intrinsic Pathway

**Figure 3:**
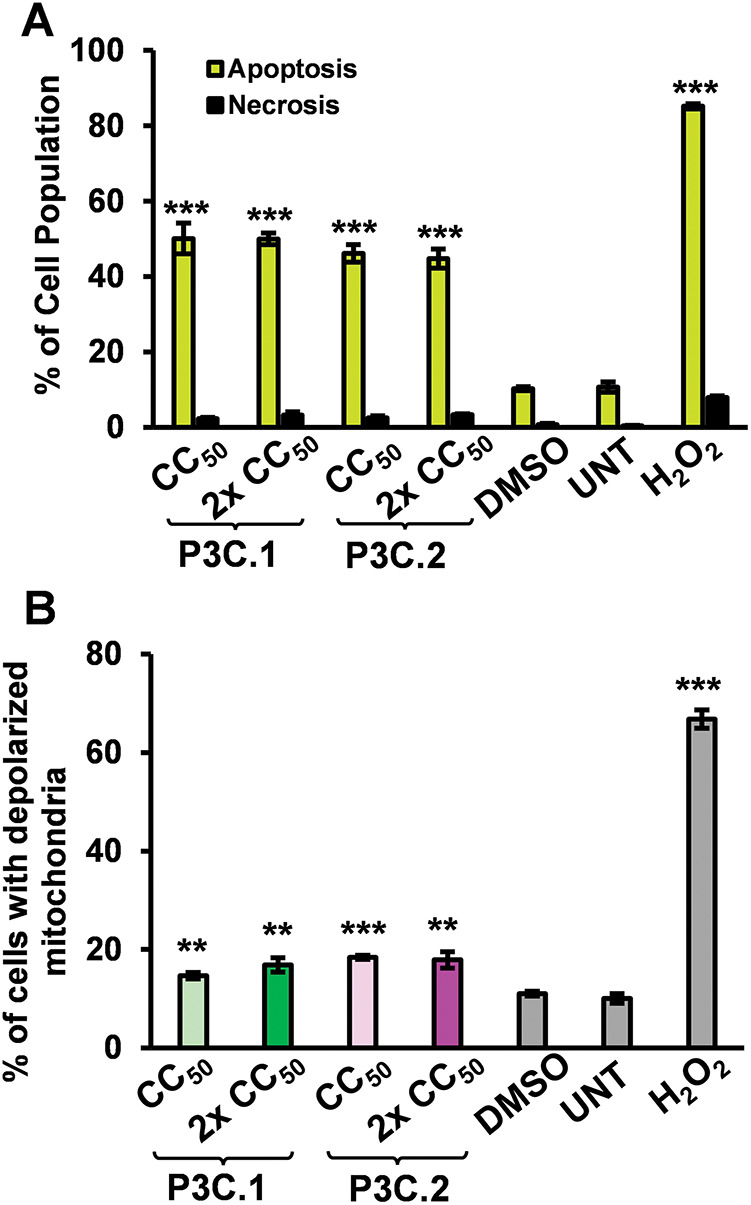

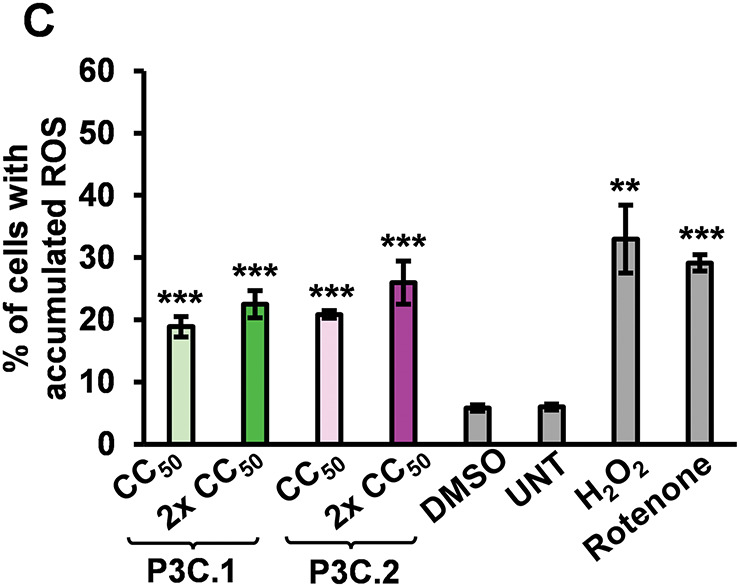
P3C.1 and P3C.2 induce activation of the apoptotic intrinsic pathway and accumulation of reactive oxygen species (ROS) in MDA-MB-231 cells. (**A**) Analysis of phosphatidylserine externalization in cell membranes after 24 h of P3C.1 and P3C.2 treatment was accomplished using the Annexin V-FITC PI kit and flow cytometry. The bar graph indicates the total apoptosis values (early and late apoptosis) for each treatment. (**B**) Evaluation of the mitochondrial membrane potential was performed after 5 h of exposure to P3C.1 and P3C.2 in MDA-MB-231 cells using the MitoProbe JC-1 assay kit and flow cytometry. (**C**) Evaluation of ROS accumulation in TNBC cells exposed for 18 h to P3C.1 and P3C.2 was achieved by using the oxidative stress indicator Carboxy-H_2_DCFDA reagent and flow cytometry. Statistical analyses were performed using a two-tailed paired Student’s *t*-test, and asterisks denote statistical significance of the experimental treatments compared to DMSO control (**p < 0.01, ***p < 0.001).

During the apoptotic process, phosphatidylserine (PS), which is typically located in the inner leaflet of the plasma membrane, is exposed to the cell surface to signal the immune system for a rapid clearance of dying cells. Most anticancer drugs used in clinics today utilize the apoptotic pathway to trigger cancer cell death [21]. To investigate the ability of P3C.1 and P3C.2 to induce apoptosis in TNBC cells, the distribution of PS in cell membranes was evaluated on MDA-MB-231 cells treated with these compounds for 24 h via flow cytometry. Results confirmed that P3C.1 and P3C.2 induce significant PS externalization of greater than 44% apoptotic cells at both CC_50_ concentrations tested (Fig. 3A). These results represent the total apoptosis values resulting from a sum of early and late apoptosis stages (Fig. S1A–E).

The cell-intrinsic apoptosis pathway involves the loss of mitochondrial membrane potential, resulting from mitochondrial damage and permeabilization, which leads to the release of cytochrome c into the cytoplasm and dissipation of the membrane potential [22]. These events are pivotal to initiating the intrinsic apoptotic pathway. To further investigate whether P3C.1 and P3C.2 induce intrinsic apoptosis, the mitochondrial membrane potential of MDA-MB-231 cells treated with the compounds for 5 h was examined by flow cytometry. Our results indicated that the two compounds induce significant mitochondrial depolarization, resulting in 16.9% and 17.8% of cells exhibiting depolarized mitochondria when treated with P3C.1 and P3C.2, respectively (Figs. 3B and S2A–E). Thus, demonstrating the activation of the intrinsic apoptotic pathway.

### 3.3 P3C.1 and 2 Treatment Trigger Oxidative Stress in TNBC Cells

Excessive production of Reactive oxygen species (ROS) disrupts the oxidation-reduction equilibrium within the cell, leading to damage to nucleic acids, lipids, proteins, and other cellular components in a process known as oxidative stress [23]. Many anticancer drugs have been found to trigger intrinsic apoptosis by promoting the generation and buildup of ROS [24]. Here, we evaluated the ability of P3C.1 and P3C.2 to trigger ROS overproduction in MDA-MB-231 cells through flow cytometry. Results showed that both compounds significantly increased the amount of intracellular ROS in TNBC cells, exhibiting an increase in ROS from 5.8% in the solvent control (DMSO) to 22.5% and 25.9% on P3C.1 and P3C.2 2× CC_50_ treatments, respectively (Fig. 3C). As expected, the H_2_O_2_ and Rotenone positive control treatments resulted in 32.2% and 29.1% of cells with ROS accumulation (Figs. 3C and S3A–F). These data showed that the mechanism of cell death induced by P3C.1 and 2 is at least in part mediated by oxidative stress.

### 3.4 P3C.1 and 2 Compounds Slowdown Cell Cycle Progression by Promoting Arrest in the S and G2-M Phases

**Figure 4:**
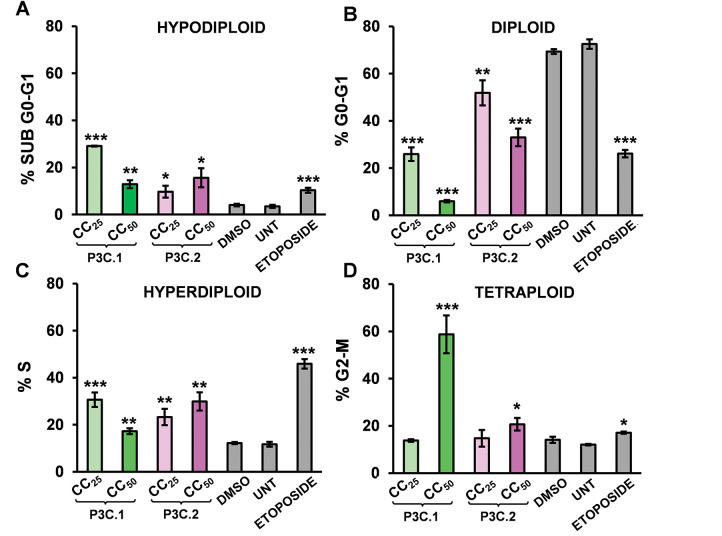

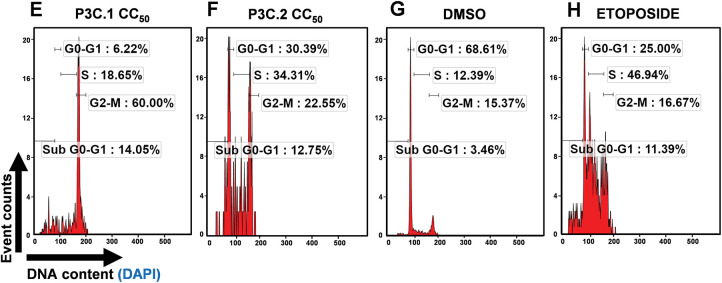
P3C.1 and P3C.2 impaired cell cycle progression by arresting MDA-MB-231 cells in the S and G2-M phases. A significant increase in cells undergoing the S and G2-M cell cycle phases were observed after treatment with P3C.1 and P3C.2. Similarly, an increment in the Sub G0-G1 (DNA fragmentation) and a decrease in the G0-G1 cell cycle phases were also seen when treating cells with both compounds. Analysis of the cell cycle profile was performed by quantifying DNA content using the NIM-DAPI solution and flow cytometry. Cells were exposed to P3C.1 and P3C.2 CC_25_, CC_50_, DMSO, and 100 µM Etoposide for 72 h. Bar graphs represent the percentage of cells in (**A**) Sub G0-G1 (hypodiploid), (**B**) G0-G1 (Diploid), (**C**) S (hyperdiploid), and (**D**) G2-M (tetraploid) phases. The FL9 detector was used to capture DAPI-bound DNA signal using the Gallios flow cytometer. Representative flow cytometry histograms are shown for each treatment (**E**–**H**). The *x* and *y* axis labels are indicated with back arrows (*y* = event counts and *x* = DNA content (DAPI) and apply to all representative single-parameter flow cytometric histograms in panels 4 (**E**–**H**). Experimental samples were compared to the DMSO control for statistical analysis. Statistically significant treatments are denoted with the following: (*) *p* < 0.05, (**) *p* < 0.01, and (***) *p* < 0.001.

Cancer cells frequently exhibit aberrant cell cycle regulation, leading to uncontrolled proliferation. Consequently, targeting cell cycle progression is a crucial goal for the development of anticancer drugs. To further examine the mechanism of cell death induced by P3C.1 and P3C.2 compounds, the effects of the pyrazoles on the cell cycle were assessed in MDA-MB-231 cells treated with the compounds for 72 h. DNA content was quantified on DAPI-stained isolated cell nuclei from each sample via flow cytometry. A significant increase in cells undergoing the S and G2-M cell cycle phases was seen in cells treated with P3C.1 and P3C.2 when compared to the solvent control (DMSO; Fig. 4C–G). As anticipated, an elevated number of cells exhibiting DNA fragmentation, indicative of apoptosis, was detected in the sub-G0-G1 subpopulation (Fig. 4A,E,F). Furthermore, a marked reduction in the G0-G1 phase population was observed, consistent with the proportional increase seen in all the other phases (Fig. 4B,E,F). Untreated and Etoposide treatment were included as controls. An expected increase in cells experiencing the S phase was seen in samples treated with Etoposide, a topoisomerase inhibitor (Fig. 4C,H). As shown in Fig. 4, cells are unable to complete DNA synthesis. Our results indicate that P3C.1 and P3C.2 disrupt the cell cycle profile by blocking progression of the S and G2-M phases in MDA-MB-231 cells, hence suppressing DNA synthesis and mitosis. Similarly, when using Jurkat cells, an S phase cell cycle arrest, as well as an increase in cells with DNA fragmentation (sub-G0-G1) were seen when applying the same experimental treatments as MDA-MB-231 cells (Fig. S4).

### 3.5 RNA-Seq Analysis of MDA-MB-231, Jurkat, and CEM Cells Identified 4 Commonly Upregulated Genes in Response to P3C.1 and P3C.2 Treatments

Gene expression signatures of MDA-MB-231, Jurkat, and CEM cells were analyzed after exposure to P3C.1 and P3C.2 for 6 h, aiming to identify drug targets and expand the understanding of their mechanisms of action.

**Figure 5:**
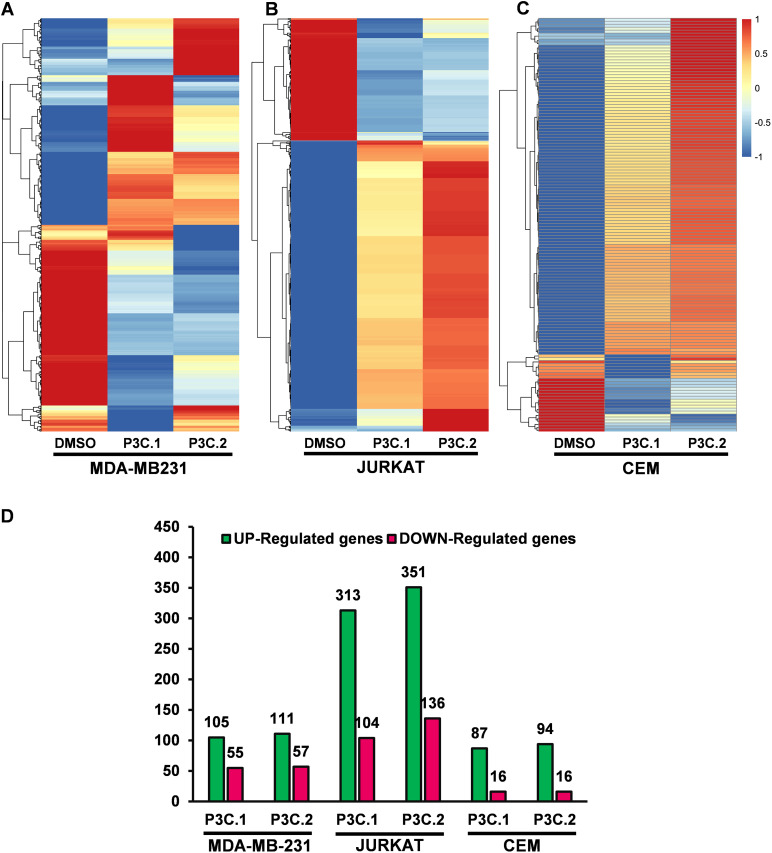
Gene expression analysis induced by P3C.1 and P3C.2 in three different cell lines. (**A**) Heatmap of differentially expressed genes of MDA-MB-231, (**B**) Jurkat and (**C**) CEM cells treated with P3C.1 and P3C.2, or with solvent control (DMSO). Each column represents the expression values of the different treatments performed for 6 h for each corresponding cell line, and each row denotes data from one transcript. The color legend of each expression values (log2 fold change) is displayed on the right. (**D**) Differentially expressed genes; up-regulated (green bars) and down-regulated (red bars) obtained for each cell line and treatments, considering only transcripts with a log2 fold change of >1.0, and a *p*-adjusted value of <0.05.

After comparing the experimental treatments to the DMSO control, the differentially expressed genes were obtained and only those with a log2 fold change of >1.0, and a *p*-adjusted value of <0.05 were considered significant and were used to generate heatmaps of each analyzed cell line (Fig. 5A–C, Tables S1–S6). P3C.1 and P3C.2 induce a similar gene expression pattern on each cell line analyzed (Fig. 5A–C). A total of 160 (105-up, and 55-down) and 168 (111-up and 57-down) DEGs were identified for P3C.1 and P3C.2 in MDA-MB-231 cells, respectively (Fig. 5D, Tables S1 and S2), whereas, in Jurkat cells, a total of 417 (313-up and 104-down) and 487 (351-up and 136-down) DEGs were observed for P3C.1 and P3C.2, respectively (Fig. 5D, Tables S3 and S4). In CEM cells, there were 103 (87-up and 16-down) and 110 (94-up and 16-down) DEGs that were affected by treatment with P3C.1 and P3C.2, respectively (Fig. 5D, Tables S5 and S6). Interestingly, the two compounds induced a relatively similar number of upregulated genes in MDA-MB-231 and CEM cell lines. However, treatment of the Jurkat cell line showed the largest number of DEGs, suggesting a stronger transcriptional response to both drugs (Fig. 5D).

**Figure 6:**
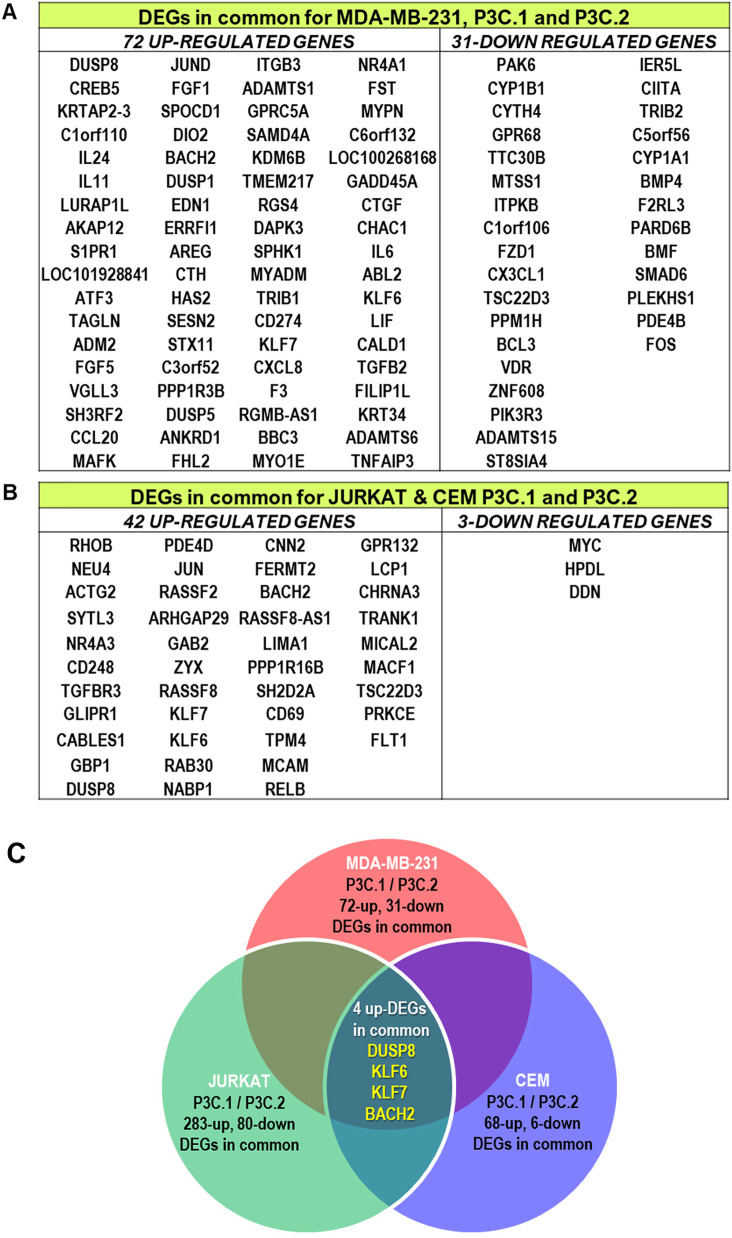
P3C.1 and P3C.2 differentially expressed genes induced in common. (**A**) MDA-MB-231, (**B**) Jurkat, and CEM common DEGs identified for both compounds. (**C**) P3C.1 and P3C.2 induced 4 up-regulated genes in common (DUSP8, KLF6, KLF7 and BACH2) in MDA-MB-231, Jurkat and CEM cell lines.

To explore a potential mechanism of action shared by the two compounds across the analyzed cell lines, DEGs affected by P3C.1 and P3C.2 in common in the TNBC cell line, MDA-MB-231, were identified. Interestingly, 72-up and 31-down DEGs were found in common by both treatments (Fig. 6A). In the two leukemia cell lines, Jurkat and CEM, there were 42 up and 3 down DEGs in common after treatment with the compounds (Fig. 6B). Lastly, when the DEGs of MDA-MB-231, Jurkat, and CEM cells were compared, only 4 genes were upregulated in common for P3C.1 and P3C.2 (Fig. 6C).

### 3.6 IPA Analysis Indicated that P3C.1 and P3C.2 Drive Kinase Signaling and Influence Membrane Dynamics

**Figure 7:**
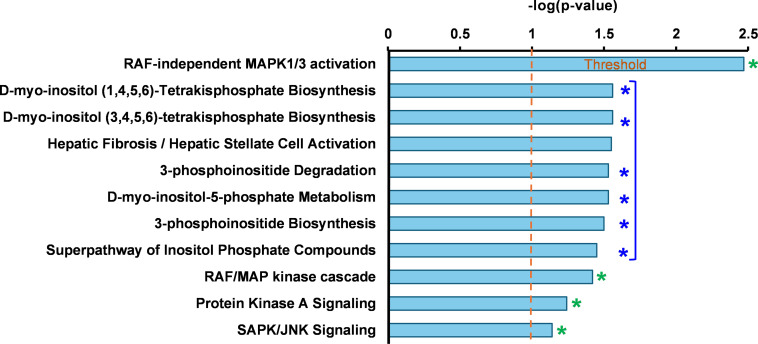
Ingenuity pathways analysis of genes commonly found in P3C.1 and P3C.2-treated MDA-MB231, JURKAT, and CEM cells, identified canonical pathways implicating these genes in kinase-mediated signal transduction (see green asterisks*), including RAF-independent MAPK1/3 activation, RAF/MAP kinase cascade, Protein Kinase A Signaling, and the SAPK/JNK signaling pathway, as well as cell signaling processes (see blue asterisks*), and membrane dynamics (see blue asterisks*). The figure shown was recreated from the original IPA histogram to enlarge features and improve legibility.

To uncover pathways potentially induced by P3C.1 and P3C.2, IPA analysis was performed with the 4 DEGs commonly found across the analyzed cell lines (Fig. 6C). As shown in Fig. 7, the pathways identified were associated with key kinase-mediated signaling cascades, including Mitogen Activated Protein Kinase (MAPK) RAF-independent activation, RAF/MAP kinase cascade, Protein Kinase A Signaling, and SAPK/JNK Signaling (Fig. 7). Pathways primarily involved in inositol phosphate metabolism and signaling were also identified. These pathways are fundamental for cell communication, membrane trafficking, gene expression, and cytoskeletal dynamics. The association of P3C.1 and P3C.2 with kinase signaling resulted from the upregulation of the Dual Specificity Phosphatases (DUSPs) proteins, specifically DUSP8, which was one of the most upregulated genes in all P3C.1 and P3C.2 treated cell lines (Fig. 6C). Additionally, DUSP1, DUSP5, DUSP6, DUSP7, DUSP8, DUSP10, DUSP16 were also found to be upregulated, most of them in P3C.1 and P3C.2 treated MDA-MB-231 and Jurkat, cells (Table 2). This widespread activation of DUSP proteins provides an essential insight into the regulation of MAPK signaling pathways by P3C.1 and P3C.2, potentially controlling the induction of apoptosis. On the other hand, Jun (also known as c-Jun) and Jun-D, which are active components of the JNK signaling pathway, were also found to be induced by P3C.1 and P3C.2 (Table 2). When altered genes shared by P3C.1 and P3C.2 on MDA-MB-231 cells were used for this analysis, these genes were found to be enriched in the S100 Family and ABRA Signaling Pathways. The other top pathways identified were the RAF-Independent MAPK1/3 and RAR activation pathways (Fig. S5A). Moreover, the canonical pathway analysis using differentially expressed genes that are common between P3C.1 and P3C.2-treated leukemia cells (Jurkat and CEM) revealed that the ILK, IL-8, and RAR signaling pathways were activated (Fig. S5B). Notably, Retinoic Acid Receptor (RAR) activation was consistently identified in both independent analyses shown above. In some cell types, such as human pancreatic cancer, the sole activation of RAR is sufficient to trigger apoptosis [25]. In addition, the RAF-independent MAPK1/3 activation pathway was observed consistently when using the genes shared across all cell lines tested (Figs. 7 and S6) and when using only those of MDA-MB-231 with both compounds (Fig. S5A).

**Table 2:** P3C.1 and P3C.2 upregulated DUSP and Jun genes in MDA-MB-231, Jurkat, and CEM cells.

Cell line	P3C.1	P3C.2
**MDA-MB-231**	DUSP1	DUSP1
	DUSP5	DUSP5
	DUSP8	DUSP8
	Jun-D	Jun-D
**JURKAT**	DUSP1	DUSP1
	DUSP6	DUSP6
	DUSP7	DUSP7
	DUSP8	DUSP8
	DUSP10	DUSP10
	DUSP16	DUSP16
	Jun	Jun
	Jun-D	Jun-D
**CEM**	DUSP8	DUSP8
	DUSP10	*N/A
	Jun	Jun

Note: *N/A: Not applicable.

In summary, each IPA analysis identified a few different canonical pathways that could be induced by P3C.1 and P3C.2, depending on the origin of the cell line: TNBC (Fig. S5A) or leukemia (Fig. S5B). Additionally, kinase cellular signaling, as well as membrane dynamics, were identified by focusing on shared genes across all cell lines tested (MDA-MB231, Jurkat, and CEM) with both compounds (Fig. 7). However, further investigation is necessary to clarify the downstream signaling events that trigger apoptosis after exposure to P3C.1 and P3C.2.

### 3.7 CMap Analyses Revealed that P3C Compounds Share Gene Signatures with Tubulin Inhibitors

**Figure 8:**
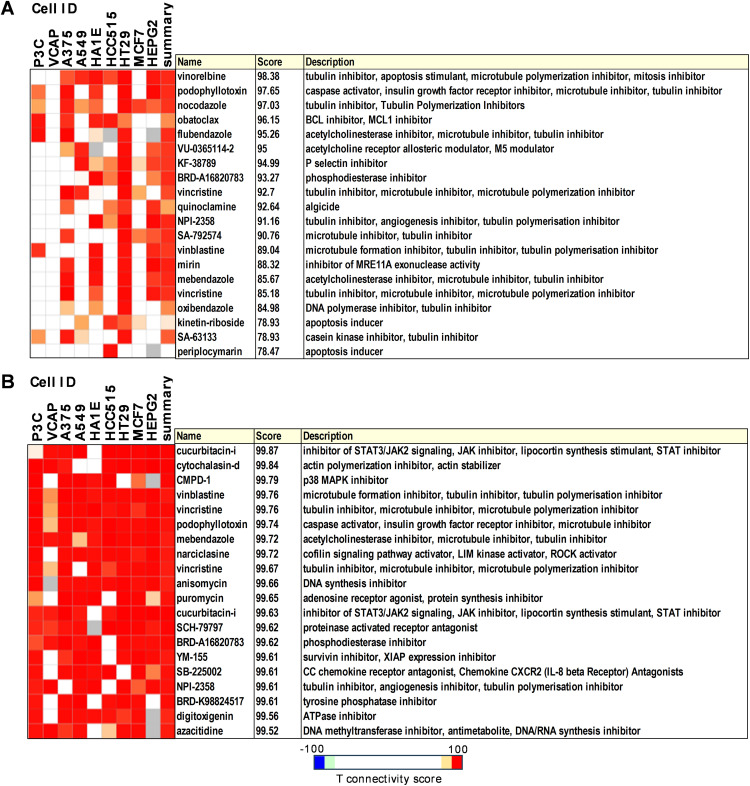
Connectivity map analyses revealed strong connectivity of P3C compounds with tubulin inhibitors. The top 20 perturbagens identified using P3C.1 and P3C.2 genes in common for (**A**) MDA-MB-231, and (**B**) Jurkat, and CEM cell lines. Tau scores of 90 or above have been previously recognized as strong and suitable for further investigation (https://clue.io).

The Connectivity map is a public database comprising of reference collections of gene-expression signatures from cultured human cell lines exposed to biologically active small molecules [26]. These datasets, combined with a pattern recognition algorithm, allow the identification of relationships among small molecules that share similar mechanisms of action. In this study, the gene expression profiles of P3C.1 and P3C.2 triggered in MDA-MB-231, Jurkat, and CEM cell lines were used to query cMAP for connections with other drugs or perturbagens. The expression pattern shared by P3C.1 and P3C.2 in MDA-MB-231 cells demonstrated a resemblance to genes affected by tubulin inhibitors (Fig. 8A). Twelve of the perturbagens identified within the top twenty were microtubule/tubulin inhibitors, such as vinorelbine, podophyllotoxin, nocodazole, flubendazole, vincristine, and vinblastine, among others. Additionally, BCL, MCL1, P-selectin, and phosphodiesterase inhibitors, as well as an acetylcholine modulator, were found to exhibit strong similarities in their gene expression patterns.

In addition, the CMap analysis of P3C.1 and P3C.2 DEGs in common between leukemic cell lines (Jurkat and CEM) identified numerous high-ranked connections (Tau score > 90, Fig. 8B). The top 20 perturbagens were included in Fig. 8B, ranging from 99.52 to 99.87 of median tau score. Interestingly, similarly to MDA-MB-231, six of the twenty strongest connections identified were microtubule inhibitors. Additionally, STAT3/JAK signaling, actin polymerization, p38, and DNA synthesis inhibitors were also identified. Notably, the similarity observed with microtubule/tubulin inhibitors in both sets of analyses emerged as a key element for further experimentation to expand the understanding of the compound’s mode of action.

### 3.8 P3C.1 and P3C.2 Disrupt Microtubule Organization in MDA-MB-231 Cells

**Figure 9:**
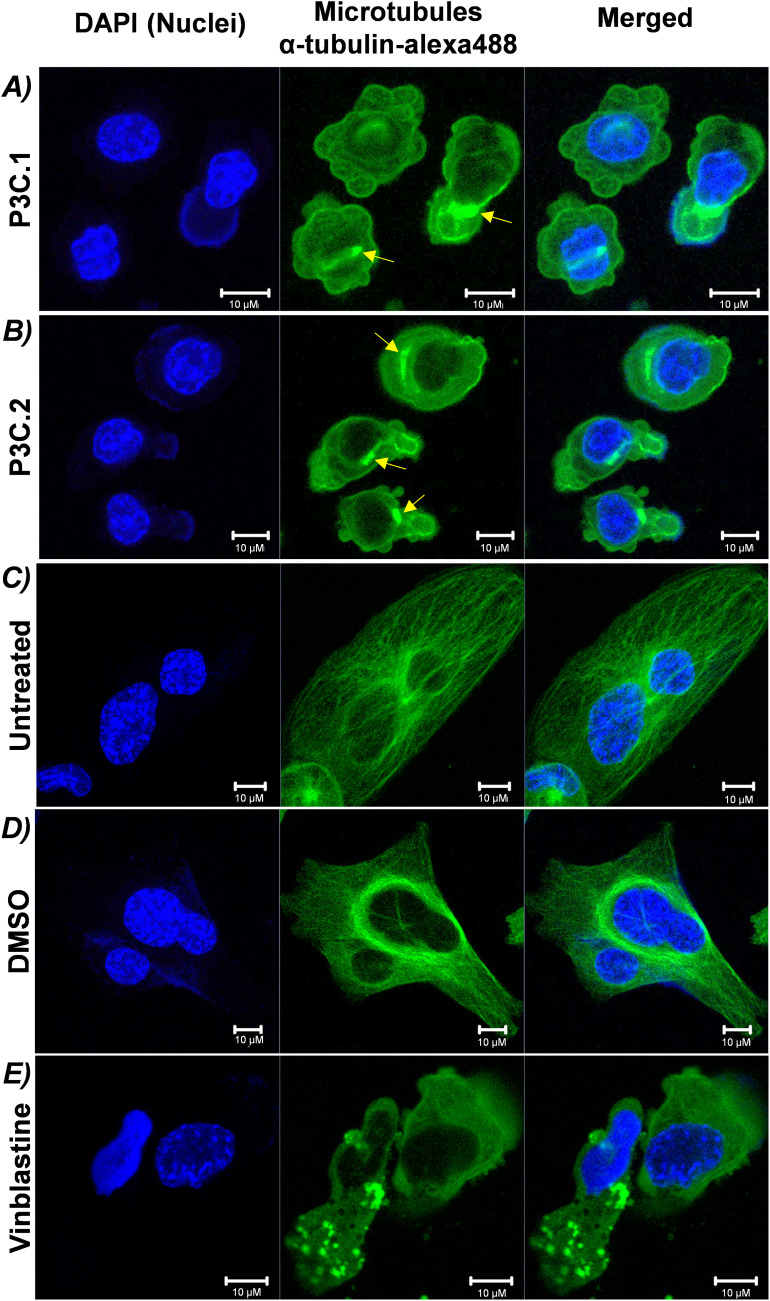
*P3C.1* and *P3C.2* disrupted the microtubule organization in triple-negative breast cancer cells. Evaluation of the cytoskeleton integrity was carried out via immunostaining and confocal microscopy. MDA-MB-231 cells treated for 2 h with the CC_50_ of (**A**) P3C.1 (0.4 µM) and (**B**) P3C.2 (0.61 µM), or (**C**) untreated, (**D**) DMSO (solvent control), and (**E**) Vinblastine (3 µM, known tubulin inhibitor) were stained with DAPI, α-tubulin/Alexa-488, for imaging the cell nucleus and microtubules, respectively. Yellow arrows indicate tubulin aggregates.

To evaluate the ability of P3C.1 and P3C.2 to disrupt tubulin, the microtubule structure and organization were examined by confocal microscopy. MDA-MB-231 cells were exposed to both drugs for 2 h, and cells were subsequently fixed, permeabilized, blocked, and stained with DAPI (Nucleus) and α-Tubulin/Alexa-488 (microtubules or tubulin). Confocal microscopy assessment of microtubules detected a complete disarrangement of microtubules when cells were treated with P3C.1 or P3C.2 (green channel; Fig. 9A,B). No fibers were visible, and tubulin appears to be covering the cytoplasm in a depolymerized form, as well as forming aggregates (see yellow arrows). Consequently, cell shrinkage was observed when compared to untreated and/or DMSO controls (Fig. 9C,D). Moreover, as expected, when cells were treated with vinblastine, a microtubule-disrupting agent used as a positive control, tubulin disorganization and aggregation were detected (Fig. 9E). These findings indicate that P3C.1 and P3C.2 behave as microtubule-targeting agents, disrupting microtubule integrity and organization.

### 3.9 P3C1 and 2 Compounds Prevent Mitotic Spindle Formation in HeLa Cells

**Figure 10:**
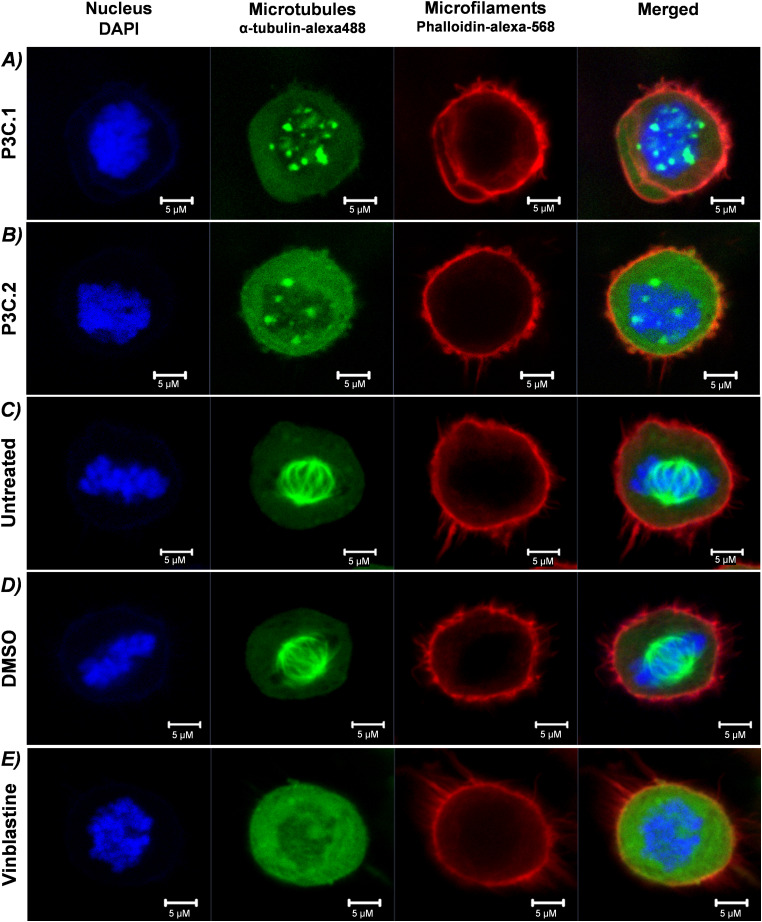
*P3C.1* and *P3C.2* treatment resulted in an impaired mitotic spindle in *HeLa* cells undergoing mitosis. Evaluation of mitotic spindle formation was performed via confocal microscopy after 2 h of exposure to (**A**) P3C.1 (0.4 µM), (**B**) P3C.2 (0.6 µM), (**C**) untreated, (**D**) DMSO (1%) solvent control, and (**E**) Vinblastine (3 µM). Treated and untreated cells were fixed, permeabilized, and stained with DAPI (nucleus), α-tubulin-alexa488 (microtubules), and Phalloidin-alexa-568 (microfilaments).

To further investigate the effects of P3C.1 and P3C.2 in microtubule dynamics, mitotic spindle formation was evaluated in cells experiencing mitosis. To facilitate identification of cells engaged in cell division, HeLa cells were analyzed since the identification of mitotic cells in HeLa cultures is common [5]. Cells were exposed to P3C.1 and P3C.2, DMSO (solvent control), and vinblastine for 2 h and stained with DAPI (nucleus), α-Tubulin/Alexa-488 (microtubules/tubulin), and phalloidin-alexa-568 (microfilaments/actin). Cells were analyzed via confocal microscopy, and mitotic cells were recognized by identifying DNA condensed into chromosomes (blue channel; DAPI), and the mitotic cell rounding shape given by the microfilaments (actin) forming a uniform round cortex (actomyosin cortex, red channel; phalloidin-alexa-568). In P3C.1 and P3C.2-treated cells, a fully impaired mitotic spindle was observed. Instead, tubulin was detected forming aggregates in a spotted pattern colocalizing in the nucleus where chromosomes appear disorganized (Fig. 10A,B). Moreover, as anticipated, untreated and solvent controls demonstrated a functional spindle formation, both experiencing metaphase and displaying aligned chromosomes awaiting segregation (Fig. 10C,D). The vinblastine treatment showed no functional spindle, and tubulin appears to be concentrated in the cytoplasm as with the P3C.1 and P3C.2 treatments (Fig. 10E). These observations confirm that P3C.1 and P3C.2 have tubulin-inhibitory effects and are consistent with the G2-M phase arrest induced by both drugs identified during the cell cycle analysis in MDA-MB-231 cells.

### 3.10 P3C.1 and P3C.2 Act by Inhibiting Tubulin Polymerization

**Figure 11:**
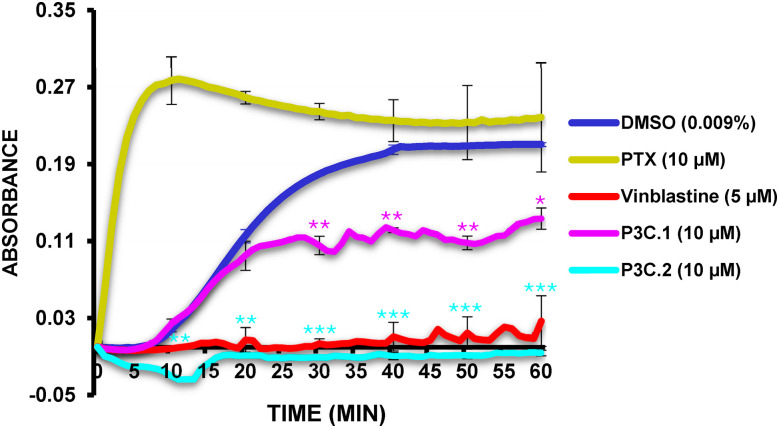
*P3C.1* and *P3C.2* disrupt microtubule polymerization. The ability of P3C compounds to inhibit tubulin polymerization was evaluated in a biochemical assay where the increase in absorbance is proportional to the rate of microtubule polymerization. Both P3C compounds significantly reduced the polymerization of tubulin, with P3C.2 exhibiting complete inhibition, closely resembling the effect of vinblastine. Each curve/line represents the average of two replicates, and asterisks denote statistical significance of P3C.1 (see purple asterisk*) and P3C.2 (see blue asterisk*) compared to the solvent control; DMSO (*p < 0.05, **p < 0.01, ***p < 0.001). To correct background interference, the data were normalized by subtracting the baseline at time zero.

To further characterize the mechanism by which P3C.1 and P3C.2 disrupt tubulin, a tubulin polymerization assay (Cytoskeleton Inc.) was conducted. This kinetic assay consists of measuring the rate of tubulin polymerization over time on a spectrophotometer as previously described [27]. P3C.1 and 2 compounds, DMSO (solvent control), vinblastine (tubulin polymerization inhibition) and paclitaxel (PTX; a microtubule stabilizer agent) were incubated with depolymerized tubulin in the presence of a general tubulin buffer containing GTP at 37°C, and measurements were taken every minute for a period of 1 h (see details in Section 2.15). Tubulin undergoes polymerization into microtubules at 37°C in the presence of GTP. Results showed that P3C.1 and P3C.2 significantly decreased tubulin polymerization when compared to the DMSO solvent control (Fig. 11). P3C.2 completely inhibited polymerization, comparable to the inhibition seen with vinblastine. Moreover, while P3C.1 also inhibited tubulin polymerization, the extent of inhibition was less pronounced compared to that induced by P3C.2. Additionally, consistent with its known mechanism of action, PTX markedly enhanced tubulin polymerization. Collectively, these findings identify P3C.1 and P3C.2 as tubulin polymerization inhibitors, corroborating prior observations in which cells fail to complete mitosis due to the absence of a functional spindle, ultimately resulting in apoptosis (see Figs. 4, 9 and 10).

### 3.11 P3C.1 and P3C.2 Alter MAP Kinase Signaling Pathways

**Figure 12:**
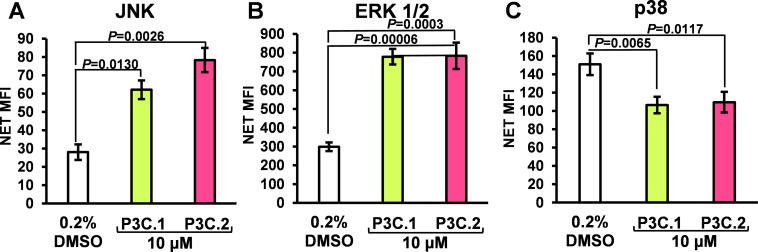
*P3C.1* and *P3C.2* simultaneously increase *JNK* and *ERK1/2* and decrease *p38* phosphorylation. Luminex xMAP technology was utilized to evaluate changes in phosphorylation induced by P3C.1 and P3C.2 in MAPK signaling proteins. MDA-MB-231 cells were exposed to P3C.1, P3C.2, and DMSO for 3 h, and cell lysates were prepared and subsequently analyzed using the Luminex Multiplexing LX100-XPONENT. (**A**–**C**) Bar graphs denote mean fluorescence intensity values (MFI) for each analyte evaluated: (**A**) JNK, (**B**) p38, and (**C**) ERK1/2. Statistical analyses were performed using a two-tailed paired Student’s *t-*test, and experimental samples were compared to the DMSO solvent control.

As suggested by the IPA analysis (Fig. 7), the potential of P3C.1 and P3C.2 to modulate MAP kinase activity was investigated using a Luminex-based multiplex platform. Using this strategy, changes in phosphorylation of the MAP kinases, ERK1/2 (Thr185/Tyr187), JNK (Thr183/Tyr185), and p38 (Thr180/Tyr182) were evaluated. MDA-MB-231 cells were exposed to 10 µM of P3C.1 or P3C.2, and 0.2% of DMSO for 3 h, and protein extracts were prepared and analyzed as mentioned in Section 2. P3C.1 and P3C.2 independently induced a significant hyperphosphorylation of JNK and ERK1/2, when compared to the DMSO control (Fig. 12A,B). Additionally, P3C.1 and P3C.2 suppressed the activity of p38, as indicated by a decrease in phosphorylation levels (Fig. 12C). These findings suggest that P3C.1 and P3C.2 initiate a complex kinase regulatory network that ultimately culminates in programmed cell death. Interestingly, the activation of JNK coincides with the upregulated expression of Jun and Jun-D genes, which are downstream effectors of phosphorylated JNK (Table 2). Likewise, the concomitant upregulation of the Dual Specificity phosphatase-8 (DUSP8) gene, which is involved in inactivating MAPK kinases, correlates with a decrease in p38 phosphorylation.

## 4 Discussion

Despite the evolution of cancer treatments over the past 200 years, the development of drug resistance is one of the main causes of therapy failure in advanced cancers [28–30]. Hence, the discovery and advancement of new anticancer treatments are of high importance. In this study, the anticancer potential of two novel pyrazole-3-carbohydrazide derivatives, P3C.1 and P3C.2, was investigated (Fig. 1). These new compounds presented here were identified as analogues of the previously published precursor P3C compound [5]. Due to structural similarities, the mode of action of the new drugs was examined in comparison to P3C. The structures differ in that P3C has the most extended aromatic system with two fused benzene rings (naphthyl), bearing a hydroxyl group (-OH; Fig. 1A) [5]. P3C.1 is the simplest structure with a benzylidene ring carrying a bromine (Br) substituent (Fig. 1B). In contrast, P3C.2 is the most functionalized and polar structure due to having hydroxyl (-OH) and methoxy groups (CH_3_O-) as substituents on the aromatic (benzylidene) ring (Fig. 1C). These groups enhance polarity, naturally increasing the compound’s aqueous solubility, also enabling hydrogen bonding interactions, which can potentially affect the biological activity of the drug. The ability of P3C.1 and P3C.2 to kill cancer cells was tested in 27 different cancer cell lines with diverse tissue origins and two non-cancerous; breast and fibroblast cell lines. Both drugs were able to efficiently kill all tested cell lines with low cytotoxic concentrations (CC_50_s, Table 1). Non-cancerous cells were included to calculate the selective cytotoxicity index (SCI) values, which represent the selectivity of a drug to a specific cancer cell line without inducing significant damage to non-cancerous cells. In general, P3C.1 and P3C.2 induced similar damage to cancerous and non-cancerous cells, with P3C.1 being slightly more selective than P3C.2 in the adherent breast MDA-MB-231 and melanoma A375 cell lines (Table 1). MDA-MB-231 cells were chosen for further investigation of the mechanism of action of the drugs, not only due to their high sensitivity to both drugs (Fig. 2), but also for comparison purposes with the formerly reported P3C compound [5]. Apoptosis was identified as the mechanism of cell death induced by P3C.1 and P3C.2, evidenced by the significant increase in phosphatidylserine externalization seen on compound-treated cells (Figs. 3A and S1A,B). It was also determined that P3C.1 and P3C.2 induced a loss of mitochondrial membrane potential after 5 h of treatment (Figs. 3B and S2A,B). This event is a universal indicator of apoptosis occurring via the intrinsic mitochondrial-mediated apoptotic pathway and can be triggered by different cellular stressors such as ROS, Ca^+^ accumulation, and as a response to drug exposure [31,32]. Here, we observed a significant buildup of intracellular ROS after P3C.1 and P3C.2 treatment in MDA-MB-231 cells (Figs. 3C and S3A,B), indicating its potential contribution to mitochondrial depolarization and apoptosis. Cell cycle analysis of MDA-MB-231 cells exposed to P3C.1 and P3C.2 revealed an interruption or arrest in the progression of the S and G2/M phases of the cell cycle (Fig. 4C,D). Although similar results were obtained with Jurkat cells, where an S phase arrest was detected, no significant increase was observed in cells progressing through the G2-M cell cycle phase (Fig. S4). These data suggest a complex mode of action of the drugs that involves a cell-dependent blockage of DNA replication and/or mitosis. Pyrazoles have been found to induce cell cycle alterations by disrupting processes crucial to DNA synthesis and cell proliferation. For example, some have been described as DNA-binding agents [33–35], which can generate DNA strand breaks or replication stalling. Additionally, these include ligase [36], topoisomerase [34,37], and microtubule inhibitors [5,12,17,38]. Ligase inhibitors prevent DNA ligase from joining DNA breaks, thereby halting the DNA repair process and DNA synthesis, which leads to genome instability and to incomplete DNA replication [36]. Similarly, topoisomerase inhibitors block the enzyme’s ability to break and rejoin DNA strands, thereby preventing the correct unwinding of DNA during replication and transcription [37]. These various assaults prevent DNA replication progression, resulting in cell cycle arrest in the S phase. On the other hand, microtubule inhibitors commonly induce incomplete G2/M phase progression due to their inability to form a mitotic spindle that can properly segregate chromosomes [5,12,17,38].

To gain additional insight into the action pathways triggered by P3C.1 and P3C.2, a transcriptomic analysis was performed on MDA-MB-231, Jurkat, and CEM cells exposed to these drugs. As expected, the gene expression resulted in similar patterns between the two compounds in each cell line tested (Fig. 5A–C). The purpose of using three cell lines from two distinct breast and lymphocyte tissue types was to identify shared expression patterns across different cell types that would likely be associated with the mode of action of the drugs. In TNBC cells (MDA-MB-231), P3C.1 and P3C.2 commonly induced 72-up and 31-down regulated genes, DUSP8, and CREB5 (cAMP responsive element binding protein 5) being the most up-regulated, and FOS (FOS Proto-Oncogene, AP-1 Transcription Factor Subunit) and PLEKHS1 (Pleckstrin Homology Domain Containing S1) the most down-regulated genes in common (Fig. 6A). DUSP 8 is a dual-specificity phosphatase that can dephosphorylate JNK, ERK, and p38 MAP Kinases, downregulating the MAPK signaling pathways. Its transcription can be rapidly induced by oxidative stress, heat shock, growth factors, and others [39,40]. CREB5 is a transcriptional activator that binds to cAMP-responsive elements in the DNA to modulate genes involved in proliferation, differentiation, and cell cycle regulation [41]. Interestingly, both genes are activated as a response to stress to regulate cellular fate, while CREB5 functions as the executor of stress response genes, DUSP8 controls or silences the amount of stress response, promoting cell adaptation. In leukemic cells (Jurkat and CEM), 43 genes were found up-regulated and 3 down-regulated in common for P3C.1 and 2. DUSP8 was one of the most up-regulated genes found, along with JUN (Jun Proto-Oncogene, AP-1 Transcription Factor Subunit), GLIPR1 (GLI Pathogenesis Related 1), and SYTL3 (Synaptotagmin Like 3), and others (Fig. 6B). MYC (MYC Proto-Oncogene, BHLH Transcription Factor), HPDL (4-Hydroxyphenylpyruvate Dioxygenase Like), and DDN (Dendrin) were among the down-regulated common genes. Remarkably, MYC is a transcription factor that controls cell growth and proliferation in mammalian cells [42], and its downregulation has been associated with cell cycle arrest, specifically preventing S-phase progression [43]. MYC promotes the expression of genes that command nucleotide biosynthesis, and its attenuation significantly reduces nucleotide availability, directly affecting cell proliferation [44] via DNA replication stalling and consequently activating the S-phase checkpoint during the cell cycle. Therefore, the downregulation of MYC identified in leukemic cells can explain the phenotype of DNA synthesis blockage or inhibition seen (S-phase arrest) in Jurkat cells (Fig. S4).

When MDA-MB231, Jurkat, and CEM DEGs shared by P3C.1 and P3C.2 were compared, only 4 up-regulated common genes were found: DUSP8, KLF6 (KLF Transcription Factor 6), KLF7 (KLF Transcription Factor 7), and BACH2 (BTB Domain And CNC Homolog 2) (Fig. 6C). KLF6 and KLF7 are transcription factors and tumor suppressors. KLF6 can be activated by stress and induce apoptosis via ATF3 (Activating Transcription Factor 3) activation in prostate cancer [45]. BACH2 (BTB Domain And CNC Homolog 2) is also a transcription factor and an apoptosis inducer in response to oxidative stress, and it is important for sustaining immune homeostasis [46,47]. The consistent activation of these 4 genes by P3C.1 and P3C.2 across all three cell lines suggests their potential role in an important evolutionarily conserved transcriptional response to cellular stress, which, in this context, may trigger apoptosis.

Ingenuity pathway analysis of shared genes for MDA-MB-231, CEM, and Jurkat cells for P3C.1 and 2 (Fig. 6C) revealed the RAF-independent MAPK1/3 activation as the most significant pathway involved (Figs. 7 and S6). Activation of MAPK1/3 refers to ERK 1/2 phosphorylation, since MAPK 1 and 3 are the genes that encode ERK 2 and ERK 1, respectively. ERK 1/2 activation is commonly triggered by the RAS–RAF–MEK–ERK pathway; nevertheless, in a RAF-independent MAPK 1/3 activation, ERK 1/2 is phosphorylated without the activity of the RAF kinase [48]. Interestingly, out of the four genes in common, DUSP8 is the one associated with this pathway. Some studies have reported that DUSP8 preferentially inactivates MAPK JNK and p38 in certain cell lines [49,50], while others have also reported inactivating ERK 1/2 [51]. On the other hand, connectivity map analyses of genes commonly found for P3C.1 and P3C.2 in MDA-MB231 cells revealed that these drugs trigger gene expression signatures that resemble tubulin inhibitors. Out of the top 20 hits discovered, 12 were identified as tubulin inhibitors (Fig. 8A). Similarly, when common genes for P3C.1 and 2 found in Jurkat and CEM were used as input, six out of the top 20 hits were tubulin polymerization inhibitors. Also, a JAK/STAT signaling, p38 MAP kinase, and DNA synthesis inhibitors were found, among others (Fig. 8B). These results align with the mechanism of action of the previously reported P3C analogue as a tubulin inhibitor [5]. Microtubules are tubulin polymers that are part of the cell cytoskeleton vital for sustaining cell structure, shape, migration, trafficking, and division [4,52]. To explore the potential tubulin inhibitory activity of P3C.1 and 2, analysis of microtubule organization and integrity was performed via confocal microscopy. MDA-MB-231 cells exposed to P3C.1 and 2 displayed a complete disarrangement of microtubule fibers, and as a result, a contracted cell morphology was observed (Fig. 9A,B). Moreover, to complement and further support the phenotype detected, microtubule spindle formation was investigated in cells experiencing cell division by confocal microscopy analysis. For this approach, the HeLa cell line was utilized to facilitate the detection of cells in active mitosis, as previously described [5]. Nuclei with condensed DNA (chromosomes), and the actomyosin cortex observed in cells using the blue (DAPI) and red (phalloidin-alexa-568) channels, aid in identifying cells in mitosis. Results demonstrated that P3C.1 and 2 treatments fully abolished microtubule spindle formation in HeLa cells. This phenotype observed is evidenced by an absent spindle and a disorganized punctuated tubulin pattern in cells treated with the two compounds independently (Fig. 10A,B). These observations support that P3C.1 and P3C.2 are tubulin disrupting agents’ able to hinder mitotic progression by impeding a proper spindle assembly, also detected as a G2-M phase arrest on the cell cycle progression analysis in MDA-MB-231 cells (Fig. 4D). To examine more closely if the tubulin inhibitory activity of P3C.1 and 2 involved tubulin polymerization suppression, the rate of tubulin polymerization was measured over a period of one hour in a kinetic assay when incubating isolated tubulin and P3C.1, and/or P3C.2 independently. Data observed proved that P3C.1 and P3C.2 are tubulin polymerization inhibitors. While P3C.2 entirely inhibited microtubule polymerization, similarly to vinblastine (positive control), P3C.1 partially repressed microtubule polymerization, both at the 10 mM dose used (Fig. 11). These findings are consistent with the microtubule disruption seen by confocal microscopy and indicate that P3C.1 and P3C.2 exert their cytotoxicity by preventing microtubule polymerization, restricting cell division, and eventually triggering apoptosis. Previous reports have suggested that disruption of microtubules prompts the phosphorylation/activation of Mitogen-Activated Protein Kinases (MAP) family members [53–56]. In this study, as suggested by the transcriptomic signature seen on cells treated with P3C.1 and P3C.2, the potential regulation of MAP kinase signaling pathways by the compounds was measured. Phosphorylation levels of JNK, ERK1/2, and p38 MAP kinases were examined after 3 h of compound exposure. And interestingly, an activation of JNK and ERK1/2 signaling pathways, as well as the inactivation of the p38 pathway were detected. Coincidentally, it has been previously reported that tubulin-disrupting agents, such as vinblastine and paclitaxel, instigate JNK activation, induction of c-Jun protein, and JUN mRNA levels as a mode of apoptosis induction in breast cancer cells [57]. Moreover, inhibition of JNK rescued cells from vinblastine- and paclitaxel-induced cell death, supporting the role of JNK in apoptosis induction by these tubulin-disrupting agents. In the case of P3C.1 and P3C.2, the activation of the Stress Activated Protein Kinase (SAPK)/JNK pathway could have been induced as a response to the oxidative stress produced by the accumulation of ROS (Fig. 3C) and, due to the DNA-replication and mitotic stress also induced (Figs. 4 and 10). Here, we also noted the transcriptional upregulation of the JUN gene, encoding the c-Jun protein, in the leukemic cell lines assessed, Jurkat and CEM (Table 2), which resembles the mechanism previously discussed for vinblastine and paclitaxel in the induction of cell death. Moreover, transcriptional upregulation of the JUND gene was also identified in the TNBC cells MDA-MB-231 as well as in Jurkat (Table 2). Both JUN and JUND genes are downstream targets of activated JNK, and function as transcription factors that, depending on the cell type and stimulus, can promote survival or trigger apoptosis. This evidence suggests that P3C.1 and 2 initiate JNK activation, and consequently JUN and JUND gene overexpression (cell-type dependent) to regulate apoptosis. On the other hand, the observed activation of ERK1/2 induced by P3C.1 and 2 (Fig. 12) has also been reported to be induced by other microtubule-disruptive agents. Similarly, as in P3C.1 and P3C.2, Guo et al. 2012, reported that Nocodazole induced the activation of ERK1/2 to increase MKP-1 protein levels, which in turn inhibited p38 activity [53]. MKP-1 protein is encoded by the DUSP1 gene and is part of the family of Dual Specificity Phosphatases that negatively regulate MAPK activity, by dephosphorylating p38, JNK, and ERK1/2 [58]. In relation to P3C.1 and P3C.2, the activation of ERK1/2 could have promoted the feedback transcriptional activation of DUSP8, which resulted in the deactivation of p38. However, additional studies are needed to verify this interaction. Moreover, although the role of activated ERK1/2 is typically associated with cell survival, the anticancer drug cisplatin and other polyphenolic compounds such as honokiol and resveratrol have been reported to induce intrinsic mitochondrial-mediated apoptosis in an ERK1/2 activation-dependent manner [59–61]. Although the previously characterized P3C compound [5] demonstrated, similar to P3C.1 and P3C.2, an activation of the JNK and deactivation of the p38 pathways, it downregulated the ERK1/2 signaling pathway. Thus, the changes induced by the compounds in the regulation of MAPK signaling pathways demonstrate the complexity of the regulatory mechanisms involved in the induction of early apoptosis.

The main limitation of this research is the absence of *in-vivo* experiments to evaluate systemic toxicity and antitumor efficacy of P3C.1 and P3C.2. Further studies, including *in-vivo* functional assays, need to be performed to fully characterize the anticancer activity of these compounds. In addition, the potential synergistic effects of P3C.1 and P3C.2 in combination with other known chemotherapeutic agents were not explored. Finally, the four common DEGs identified as potential mediators of the cellular response P3C.1 and P3C.2 in MDA-MB-231, Jurkat, and CEM cells were not experimentally validated.

## 5 Conclusion

Collectively, the results presented here identify P3C.1 and P3C.2 as new pyrazole-containing compounds with potent anticancer activity, mainly against TNBC, which can inhibit cell growth by disrupting tubulin polymerization and thus cell division. P3C.1 and P3C.2 also induce oxidative stress and hinder DNA replication during the cell cycle. Additionally, the results suggest that these new drugs modulate MAP kinase signaling pathways to control cell death.

## Supplementary Materials



## Data Availability

The data that support the findings of this study are available from the corresponding authors [Renato J. Aguilera and Denisse A. Gutierrez] upon request.

## Supplementary Materials

The supplementary material is available online at https://www.techscience.com/doi/10.32604/or.2026.074945/s1.
