# Supplementary material for: Discovery of Two Novel Pyrazole Derivatives as Anticancer Agents Targeting Tubulin Polymerization and MAPK Signaling Pathways
Source: Oncol Res. 2026 Mar 23;34(4):14. doi: 10.32604/or.2026.074945 (PMC13040279; doi:10.32604/or.2026.074945)
Supplement: Supplementary file 1 [file OncolRes-34-74945-s001.docx]

**SUPPLEMENTARY INFORMATION.**

**Discovery of two Novel Pyrazole derivatives as anticancer agents targeting tubulin polymerization and MAPK/JNK/SAPK and ERK1/2 signaling pathways activation.**

Denisse A. Gutierrez^1^*, Elisa Robles-Escajeda^1^, Soumya Nair^1^, Jose A. Lopez-Saenz^1^, Edgar A. Borrego^1^, Ana P. Betancourt^1^, Sourav Roy^1^, Robert A. Kirken^1^, Armando Varela-Ramirez^1^ and Renato J. Aguilera^1^*.

^1^Cellular Characterization and Biorepository Core Facility, Border Biomedical Research Center, Department of Biological Sciences, College of Science, The University of Texas at El Paso, 500 West University Avenue, El Paso, TX 79968-0519, USA.

*Corresponding authors: DAG, [dagutierrez18@miners.utep.edu](mailto:dagutierrez18@miners.utep.edu) and RJA, [raguilera@utep.edu](mailto:raguilera@utep.edu).

***Table of Contents***

**Supplementary figure S1. Descriptive flow cytometry density dot plots used to calculate early and late apoptotic cell populations on MDA-MB231 cells exposed to P3C.1 and P3C.2.**

**Supplementary figure S2. Descriptive flow cytometry density dot plots utilized to identify percentages of cells with depolarized mitochondria.**

**Supplementary figure S3.** **Representative flow cytometry density dot plots utilized to quantify percentages of MDA-MB-231 cells with accumulated ROS.**

**Supplementary figure S4. P3C.1 and P3C.2 treatments induce S-phase cell cycle arrest in Jurkat cells.**

**Supplementary figure S5. Top 20 ingenuity canonical pathways determined for A) MDA-MB-231 and B) Jurkat/CEM P3C.1 and P3C.2 shared genes.**

**Supplementary figure S6. RAF-independent MAPK1/3 activation pathway (non-canonical ERK1/2 activation) identified by IPA on DEGs shared by MDA-MB231, Jurkat and CEM, with P3C.1 and P3C.2**

**Supplementary table S1. Differentially Expressed Genes (DEG’s) identified in MDA-MB-231 cells treated with P3C.1.**

**Supplementary table S2. Differentially Expressed Genes (DEG’s) identified in MDA-MB-231 cells treated with P3C.2.**

**Supplementary table S3. Differentially Expressed Genes (DEG’s) identified in Jurkat cells treated with P3C.1.**

**Supplementary table S4. Differentially Expressed Genes (DEG’s) identified in Jurkat cells treated with P3C.2.**

**Supplementary table S5. Differentially Expressed Genes (DEG’s) identified in CEM cells treated with P3C.1.**

**Supplementary table S6. Differentially Expressed Genes (DEG’s) identified in CEM cells treated with P3C.2.**

**Supplementary figure S1. Descriptive flow cytometry density dot plots used to calculate early and late apoptotic cell populations on MDA-MB231 cells exposed to P3C.1 and P3C.2.** The following treatments were included: **(A)** P3C.1 2x CC_50_, **(B)** P3C.2 2x CC_50_, **(C)** DMSO (vehicle control), **(D)** Untreated (UNT), and **(E)** H_2_O_2_ (apoptosis inducer). In each plot, dots represent individual cells, and color a density gradient, grey being a low-density area and red a high-density area. The FL1 detector was used to identify green-fluorescent cells (annexin-FITC positive), denoting the early apoptotic subpopulation, as indicated at the bottom right quadrant in the dot plots. Moreover, the FL1 and FL2 detectors were used for identifying the late apoptotic population emitting a green and red fluorescent signal, respectively, located in the top right quadrant. PI-positive cells were denoted as necrotic, found in the top left quadrant of each dot plot.

**Supplementary figure S2. Descriptive flow cytometry density dot plots utilized to identify percentages of cells with depolarized mitochondria.** MDA-MB-231 cells were exposed to: **(A)** P3C.1 2x CC_50_, **(B)** P3C.2 2x CC_50_, **(C)** DMSO (vehicle control), **(D)** Untreated (UNT), and **(E)** H_2_O_2_ (oxidative stress inducer) for 5h. Percentages shown denote cells with depolarized mitochondria, identified by a shift from red/green to only green-fluorescent signal, as shown at the bottom left of the flow cytometry dot plots (A-E; oval gate).

**Supplementary figure S3.** **Representative flow cytometry density dot plots utilized to quantify percentages of MDA-MB-231 cells with accumulated ROS.** Cells were exposed to the following treatments for 18h: **(A)** P3C.1 2x CC_50_, **(B)** P3C.2 2x CC_50_, **(C)** DMSO, **(D)** Untreated (UNT), **(E)** 1 mM H_2_O_2_, and **(F)** 10 µM Rotenone (ROS inducer). Cells with accumulated ROS emitted a green-fluorescent signal, seen on the right side of the flow cytometric dot plots.

**Supplementary figure S4. P3C.1 and P3C.2 treatments induce S-phase cell cycle arrest in Jurkat cells.** Quantification of DNA content was accomplished by flow cytometry after 72 h of exposure to compounds. Each bar graph displays the percentage of cells in the different cell cycle phases; (A) Sub G0-G1, (B) G0-G1, (C) S, and (D) G2-M phases. Flow cytometry histograms are shown for each treatment: (E) P3C.1 CC_12.5_, (F) P3C.2 CC_12.5_, (G) DMSO, and (H) ETOPOSIDE. Treatments that were statistically significant when compared to DMSO are indicated with the following asterisk: (*) p<0.05, (**) p<0.01, and (***) p<0.001.

**Supplementary figure S5. Top 20 ingenuity canonical pathways determined for A) MDA-MB-231 and B) Jurkat/CEM P3C.1 and P3C.2 shared genes.**(*) p<0.05

**Supplementary figure S6. RAF-independent MAPK1/3 activation pathway (non-canonical ERK1/2 activation) identified by IPA on DEGs shared by MDA-MB231, Jurkat and CEM, with P3C.1 and P3C.2.** Diagrams display how this pathway is regulated by DUSP phosphatases, either cytosolic (A) or nuclear (B), which can be transcriptionally induced by ERK1/2 phosphorylation to control ERK1/2 transient or sustained activity.

**Supplementary table S1. Differentially Expressed Genes (DEG’s) identified in MDA-MB-231 cells treated with P3C.1.**

| **DEG’s identified in MDA-MB-231 cells exposed to P3C.1** | | | |
| --- | --- | --- | --- |
| **Gene** | **log2FoldChange** | **pvalue** | **padj** |
| KRTAP2-3 | 3.305460106 | 1.02466E-47 | 2.55461E-45 |
| DUSP8 | 2.999064421 | 5.30653E-64 | 2.49898E-61 |
| C1orf110 | 2.632054 | 2.89867E-52 | 8.19035E-50 |
| NR4A3 | 2.425040559 | 3.6906E-26 | 4.64614E-24 |
| CREB5 | 2.383928502 | 2.67996E-51 | 7.40775E-49 |
| DIO2 | 2.112281276 | 4.86655E-30 | 7.03161E-28 |
| HAS2 | 2.044781875 | 4.55505E-92 | 4.13696E-89 |
| FILIP1L | 1.968596047 | 8.23674E-25 | 9.52092E-23 |
| ERRFI1 | 1.917881225 | 4.535E-210 | 2.8831E-206 |
| ADAMTS6 | 1.917546141 | 3.88064E-58 | 1.37062E-55 |
| SH3RF2 | 1.916584753 | 3.36258E-53 | 9.71708E-51 |
| CCL20 | 1.91019541 | 8.09135E-08 | 2.10392E-06 |
| CRISPLD2 | 1.864965575 | 2.47747E-64 | 1.26004E-61 |
| FST | 1.853554509 | 2.34564E-77 | 1.56973E-74 |
| CD274 | 1.830493003 | 6.4549E-134 | 1.1725E-130 |
| CXCL8 | 1.827689485 | 3.294E-159 | 1.0471E-155 |
| LOC101928841 | 1.821796437 | 6.69454E-30 | 9.56417E-28 |
| DUSP5 | 1.763871896 | 3.123E-136 | 6.6181E-133 |
| ARID5B | 1.736885964 | 1.16118E-49 | 3.07591E-47 |
| LURAP1L | 1.702587337 | 0.00019315 | 0.0021326 |
| TM4SF1 | 1.694036826 | 7.6726E-104 | 8.1298E-101 |
| KRT34 | 1.67400889 | 6.10948E-05 | 0.000808346 |
| IL6 | 1.663109919 | 2.4634E-105 | 2.8475E-102 |
| PLCXD2 | 1.620212929 | 1.36851E-05 | 0.0002211 |
| TNFAIP3 | 1.61757092 | 1.51563E-78 | 1.07062E-75 |
| MAFK | 1.61119086 | 7.61554E-72 | 4.21007E-69 |
| IL11 | 1.60907525 | 2.4145E-166 | 1.0234E-162 |
| ADAMTS1 | 1.583028958 | 2.40676E-55 | 7.84667E-53 |
| RGS4 | 1.568872578 | 5.32098E-34 | 9.14274E-32 |
| TRIB1 | 1.533441901 | 4.89617E-35 | 8.6465E-33 |
| S1PR1 | 1.508343603 | 6.0974E-09 | 1.91902E-07 |
| CXCL2 | 1.492919201 | 5.55111E-74 | 3.36106E-71 |
| EDN1 | 1.475451787 | 2.85613E-59 | 1.10047E-56 |
| MYPN | 1.471074137 | 4.61054E-08 | 1.24201E-06 |
| TPPP | 1.460506033 | 3.69015E-08 | 1.02004E-06 |
| HBEGF | 1.443891019 | 1.7671E-138 | 4.4938E-135 |
| EPHA5 | 1.44058794 | 0.001080716 | 0.008859639 |
| JUND | 1.437787937 | 8.87408E-12 | 3.90429E-10 |
| F3 | 1.419571872 | 3.1198E-123 | 4.4076E-120 |
| TRAF1 | 1.412128918 | 4.97004E-55 | 1.57985E-52 |
| ADM2 | 1.409967997 | 2.56974E-06 | 5.03454E-05 |
| CDKN1A | 1.381316926 | 3.2082E-106 | 4.0793E-103 |
| GADD45A | 1.379443274 | 1.18142E-80 | 8.8363E-78 |
| KLF7 | 1.375992052 | 1.65294E-18 | 1.32183E-16 |
| CSGALNACT1 | 1.37391557 | 1.41855E-18 | 1.14884E-16 |
| CHAC1 | 1.365800195 | 1.50938E-18 | 1.21467E-16 |
| CTH | 1.345283836 | 3.02005E-47 | 7.38461E-45 |
| IL24 | 1.341567946 | 7.47129E-54 | 2.31701E-51 |
| IL12A | 1.336915617 | 1.63875E-07 | 4.04596E-06 |
| SPOCD1 | 1.325328088 | 2.28558E-19 | 1.96359E-17 |
| NR4A1 | 1.323012838 | 1.61799E-09 | 5.42816E-08 |
| TGFB2 | 1.317182879 | 1.89061E-76 | 1.20196E-73 |
| AREG | 1.312243305 | 1.36886E-53 | 4.14406E-51 |
| BACH2 | 1.311713579 | 1.88628E-09 | 6.27853E-08 |
| RGMB-AS1 | 1.300200189 | 0.001593517 | 0.012103683 |
| GPRC5A | 1.290923253 | 8.8435E-63 | 3.74817E-60 |
| HAS2-AS1 | 1.290291666 | 7.55539E-05 | 0.000970371 |
| SESN2 | 1.284928277 | 1.64926E-30 | 2.49647E-28 |
| AKAP12 | 1.268639788 | 2.96762E-38 | 5.80513E-36 |
| NHS | 1.265208157 | 1.21461E-25 | 1.47083E-23 |
| DAPK3 | 1.235557439 | 6.22454E-48 | 1.5829E-45 |
| INHBA | 1.234647866 | 1.52374E-11 | 6.63506E-10 |
| FHL2 | 1.232082081 | 9.90747E-61 | 3.93667E-58 |
| RNASEK-C17orf49 | 1.229290258 | 0.000406007 | 0.003931748 |
| SOCS2 | 1.229154593 | 6.05616E-11 | 2.47602E-09 |
| VGLL3 | 1.212324232 | 1.21317E-12 | 5.84093E-11 |
| KLF6 | 1.212057932 | 8.183E-63 | 3.58782E-60 |
| IL1A | 1.20735869 | 2.19114E-27 | 2.84289E-25 |
| BBC3 | 1.204956246 | 5.85685E-14 | 3.22289E-12 |
| CTGF | 1.19909085 | 5.96164E-87 | 5.05348E-84 |
| ANKRD1 | 1.192061656 | 1.6088E-53 | 4.7572E-51 |
| STX11 | 1.188023122 | 1.38588E-07 | 3.47219E-06 |
| SAMD4A | 1.18710873 | 2.58998E-50 | 7.00673E-48 |
| C6orf132 | 1.183983911 | 2.80316E-38 | 5.56909E-36 |
| ITGB3 | 1.175162146 | 8.36518E-06 | 0.00014354 |
| ETS1 | 1.168683488 | 6.21014E-64 | 2.82007E-61 |
| KDM6B | 1.161094651 | 6.24951E-16 | 3.99309E-14 |
| FGF5 | 1.154794588 | 1.45253E-09 | 4.92505E-08 |
| NEXN | 1.152764307 | 3.68022E-27 | 4.6794E-25 |
| LIF | 1.151095919 | 1.15816E-45 | 2.77849E-43 |
| PPP1R13L | 1.14219249 | 1.8009E-23 | 1.95713E-21 |
| CLDN1 | 1.140611683 | 2.6424E-14 | 1.51343E-12 |
| MYADM | 1.136188005 | 3.9105E-64 | 1.91239E-61 |
| LOC100268168 | 1.134583414 | 0.004384201 | 0.027047605 |
| TAGLN | 1.121849397 | 1.4219E-19 | 1.24686E-17 |
| SRF | 1.121723136 | 1.75718E-55 | 5.87962E-53 |
| CALD1 | 1.121327935 | 1.28602E-58 | 4.80933E-56 |
| SPHK1 | 1.110360812 | 8.07804E-17 | 5.55202E-15 |
| DUSP1 | 1.105980098 | 8.80041E-44 | 1.99816E-41 |
| ATF3 | 1.098553076 | 3.00977E-15 | 1.85773E-13 |
| GBP1 | 1.097924317 | 8.15837E-39 | 1.64657E-36 |
| CSRP1 | 1.08825478 | 7.41454E-66 | 3.92816E-63 |
| CSRNP1 | 1.087457582 | 4.5468E-39 | 9.32461E-37 |
| FGF1 | 1.084470405 | 2.6139E-06 | 5.09825E-05 |
| PPP1R3B | 1.07959943 | 1.73402E-27 | 2.27299E-25 |
| C3orf52 | 1.067939874 | 3.5436E-19 | 3.00379E-17 |
| RND3 | 1.061987279 | 2.83378E-40 | 6.10703E-38 |
| PDCD1LG2 | 1.058948164 | 1.4817E-13 | 7.81737E-12 |
| TMEM217 | 1.055218856 | 1.03311E-05 | 0.000172841 |
| UBALD1 | 1.049029942 | 8.13441E-11 | 3.24229E-09 |
| MYO1E | 1.046849644 | 2.16884E-25 | 2.60158E-23 |
| PPP1R15A | 1.044612157 | 3.53941E-62 | 1.45173E-59 |
| TANC2 | 1.030125954 | 2.54684E-27 | 3.27102E-25 |
| SLC20A2 | 1.028155233 | 4.46504E-33 | 7.47013E-31 |
| ABL2 | 1.012031615 | 8.7207E-32 | 1.40359E-29 |
| CRISPLD1 | -1.002564569 | 4.50614E-05 | 0.000625498 |
| HES1 | -1.010480286 | 0.000625868 | 0.005657073 |
| KGFLP1 | -1.011707202 | 0.005984111 | 0.034273863 |
| PLLP | -1.016200175 | 0.001107834 | 0.009052768 |
| TTC30B | -1.022648849 | 4.38932E-05 | 0.000612626 |
| FZD1 | -1.023012045 | 8.04065E-31 | 1.24679E-28 |
| VDR | -1.0303062 | 9.77936E-18 | 7.27161E-16 |
| GBP4 | -1.033246564 | 2.02107E-09 | 6.64028E-08 |
| ZFP14 | -1.039294356 | 2.42794E-05 | 0.000364908 |
| CX3CL1 | -1.05239173 | 1.1837E-05 | 0.000194455 |
| CYP39A1 | -1.059745205 | 0.008235467 | 0.043831715 |
| HEXIM2 | -1.06302763 | 9.23489E-05 | 0.00114894 |
| PIK3R3 | -1.065729069 | 5.38624E-16 | 3.45889E-14 |
| NOL4L | -1.072632463 | 7.36169E-06 | 0.000127874 |
| TSC22D3 | -1.07750126 | 3.24347E-13 | 1.63654E-11 |
| ZNF436-AS1 | -1.079816758 | 0.009261709 | 0.047542442 |
| XAF1 | -1.102984342 | 2.73784E-06 | 5.31476E-05 |
| KCNN3 | -1.108852216 | 0.000679505 | 0.006029245 |
| NEURL1B | -1.118499289 | 2.03165E-18 | 1.61453E-16 |
| MTSS1 | -1.131000413 | 0.005551919 | 0.032486266 |
| PAK6 | -1.131623122 | 1.81996E-07 | 4.46734E-06 |
| ZNF608 | -1.147234428 | 3.33163E-12 | 1.54605E-10 |
| TRAM1L1 | -1.16079035 | 0.001701208 | 0.012799322 |
| FEZF1 | -1.189117763 | 2.50766E-06 | 4.95108E-05 |
| ATOH8 | -1.208850163 | 0.002820805 | 0.019083704 |
| C5orf56 | -1.215102979 | 0.006641431 | 0.036988962 |
| DHRS3 | -1.239483978 | 0.001650398 | 0.012490961 |
| TRIB2 | -1.271505402 | 3.14362E-56 | 1.0803E-53 |
| METTL7A | -1.287383658 | 2.61655E-13 | 1.35242E-11 |
| ST8SIA4 | -1.29054188 | 1.41712E-09 | 4.81782E-08 |
| C1orf106 | -1.323971651 | 1.84836E-08 | 5.41519E-07 |
| FAM13C | -1.327375465 | 0.001504514 | 0.011593878 |
| PIK3C2B | -1.336321104 | 3.0565E-06 | 5.85291E-05 |
| CYTH4 | -1.36417453 | 0.002112919 | 0.015204171 |
| GPR68 | -1.37737614 | 2.99854E-58 | 1.08933E-55 |
| CIITA | -1.394678384 | 3.24741E-23 | 3.4409E-21 |
| RGPD8 | -1.406196632 | 0.004252951 | 0.026340124 |
| PPM1H | -1.455778727 | 0.000462376 | 0.004384122 |
| ITPKB | -1.47459155 | 4.27857E-07 | 9.87333E-06 |
| BMP4 | -1.48112009 | 2.77444E-93 | 2.71362E-90 |
| LFNG | -1.573162749 | 4.02478E-13 | 1.98353E-11 |
| CYP1A1 | -1.601026338 | 4.31443E-23 | 4.46E-21 |
| PARD6B | -1.610208008 | 1.48976E-48 | 3.86578E-46 |
| BCL3 | -1.678138748 | 4.72962E-30 | 6.91231E-28 |
| ADAMTS15 | -1.722783622 | 7.05168E-85 | 5.60389E-82 |
| IER5L | -1.73998849 | 4.46377E-14 | 2.46769E-12 |
| CYP1B1 | -1.771581347 | 5.6785E-130 | 9.0252E-127 |
| LVCAT1 | -1.983978734 | 5.62836E-05 | 0.000750153 |
| PDE4B | -2.105275763 | 4.3593E-26 | 5.43417E-24 |
| F2RL3 | -2.233333236 | 1.40438E-13 | 7.4403E-12 |
| BMF | -2.42723511 | 4.37672E-18 | 3.37273E-16 |
| PLEKHS1 | -2.435397379 | 1.38066E-09 | 4.74462E-08 |
| SMAD6 | -2.571394905 | 3.05808E-13 | 1.55534E-11 |
| FOS | -3.624300688 | 4.0983E-250 | 5.211E-246 |
| RAB4B-EGLN2 | -8.429716342 | 0.000188988 | 0.002095012 |

**Supplementary table S2. Differentially Expressed Genes (DEG’s) identified in MDA-MB-231 cells treated with P3C.2.**

| **DEG’s identified in MDA-MB-231 cells exposed to P3C.2** | | | |
| --- | --- | --- | --- |
| **Gene** | **log2FoldChange** | **pvalue** | **padj** |
| DUSP8 | 4.202458743 | 9.04171E-30 | 1.06265E-27 |
| CREB5 | 3.262954505 | 4.39258E-69 | 2.17636E-66 |
| KRTAP2-3 | 2.941594227 | 1.00189E-80 | 8.06649E-78 |
| C1orf110 | 2.639844739 | 7.28368E-43 | 1.48362E-40 |
| KRTAP2-1 | 2.507384734 | 2.99575E-08 | 6.57432E-07 |
| IL24 | 2.419541267 | 1.1473E-185 | 4.9265E-182 |
| IL11 | 2.384837094 | 0 | 0 |
| LURAP1L | 2.384435465 | 3.86066E-08 | 8.37256E-07 |
| AKAP12 | 2.29453632 | 2.4354E-193 | 1.5687E-189 |
| SERPINB2 | 2.243294878 | 3.01556E-09 | 7.72295E-08 |
| S1PR1 | 2.191343507 | 4.19046E-12 | 1.4749E-10 |
| LOC101928841 | 2.149319897 | 1.38717E-19 | 8.59111E-18 |
| ATF3 | 2.129043305 | 2.2822E-20 | 1.46997E-18 |
| TAGLN | 2.014034717 | 1.0099E-17 | 5.55962E-16 |
| VCAN | 1.996957742 | 0.000643686 | 0.005484099 |
| ADM2 | 1.976179817 | 2.028E-05 | 0.000265226 |
| FILIP1L | 1.919782757 | 1.01694E-13 | 4.09381E-12 |
| HSPB8 | 1.876333945 | 0.00015803 | 0.001606747 |
| KRT34 | 1.819929618 | 9.40381E-06 | 0.000134004 |
| KIF17 | 1.785898943 | 0.002081898 | 0.014735722 |
| FGF5 | 1.784600694 | 2.7016E-39 | 4.70297E-37 |
| VGLL3 | 1.772663429 | 1.68088E-24 | 1.45323E-22 |
| SH3RF2 | 1.706773127 | 3.59864E-57 | 1.15894E-54 |
| CCL20 | 1.705453115 | 0.000966441 | 0.007737537 |
| RELN | 1.693200729 | 1.11117E-16 | 5.65774E-15 |
| MAFK | 1.685627568 | 1.57676E-52 | 4.14527E-50 |
| HES4 | 1.670249716 | 0.000147365 | 0.001516259 |
| JUND | 1.655474064 | 3.76184E-45 | 8.07668E-43 |
| FGF1 | 1.641046644 | 2.01938E-09 | 5.30892E-08 |
| SPOCD1 | 1.633019308 | 3.54573E-23 | 2.87271E-21 |
| DIO2 | 1.614813543 | 2.42649E-05 | 0.000311646 |
| BACH2 | 1.605725172 | 9.29166E-07 | 1.6175E-05 |
| DUSP1 | 1.527894479 | 4.05314E-59 | 1.41115E-56 |
| TCP11L2 | 1.518672652 | 2.0158E-05 | 0.000264435 |
| EDN1 | 1.506398664 | 2.6531E-54 | 7.59493E-52 |
| BLOC1S5 | 1.506349712 | 3.90873E-05 | 0.000471905 |
| ERRFI1 | 1.504890283 | 6.373E-131 | 1.6419E-127 |
| AREG | 1.488911589 | 3.58828E-54 | 9.83495E-52 |
| IL6R | 1.447614981 | 0.000112799 | 0.001204871 |
| CTH | 1.446595839 | 9.0629E-25 | 8.10752E-23 |
| HAS2 | 1.414764946 | 1.9268E-21 | 1.35634E-19 |
| ADM | 1.410502378 | 3.30714E-54 | 9.26142E-52 |
| SESN2 | 1.396812689 | 6.50836E-27 | 6.49928E-25 |
| STX11 | 1.390230679 | 0.000139505 | 0.001443461 |
| ADAMTS6 | 1.386171763 | 5.19692E-40 | 9.70243E-38 |
| TNFAIP3 | 1.380084806 | 2.8064E-89 | 3.28655E-86 |
| C3orf52 | 1.369621531 | 4.26816E-24 | 3.61727E-22 |
| PPP1R3B | 1.363271893 | 6.63906E-38 | 1.09647E-35 |
| DUSP5 | 1.354930525 | 6.01532E-73 | 3.22872E-70 |
| ANKRD1 | 1.352054312 | 2.93683E-61 | 1.14643E-58 |
| FHL2 | 1.349901497 | 4.69617E-78 | 3.36089E-75 |
| LOC100507140 | 1.330721356 | 0.006741742 | 0.038157785 |
| GCSAM | 1.318528074 | 0.002529332 | 0.017276166 |
| ITGB3 | 1.313911461 | 9.72958E-08 | 1.98631E-06 |
| ADAMTS1 | 1.306838742 | 1.24349E-25 | 1.16077E-23 |
| GPRC5A | 1.298537102 | 3.87334E-80 | 2.93508E-77 |
| LAMA1 | 1.290321496 | 1.24044E-05 | 0.000172192 |
| LZTS3 | 1.285954836 | 2.68597E-10 | 7.67198E-09 |
| RHOB | 1.277665599 | 3.23732E-20 | 2.07478E-18 |
| LCP1 | 1.271079269 | 9.54584E-30 | 1.10783E-27 |
| SAMD4A | 1.270025779 | 1.2221E-113 | 2.6238E-110 |
| KDM6B | 1.268295037 | 2.80399E-22 | 2.15006E-20 |
| TMEM217 | 1.259377509 | 3.33107E-05 | 0.000411812 |
| METRNL | 1.255499882 | 5.53448E-05 | 0.000646961 |
| RGS4 | 1.252224672 | 1.05292E-28 | 1.15929E-26 |
| DAPK3 | 1.238508612 | 8.3673E-56 | 2.56637E-53 |
| SPHK1 | 1.21464722 | 3.33859E-21 | 2.29988E-19 |
| MYADM | 1.196193006 | 2.18808E-85 | 2.16822E-82 |
| TRIB1 | 1.190207282 | 7.33018E-22 | 5.39585E-20 |
| CD274 | 1.187171323 | 1.75836E-60 | 6.47177E-58 |
| KLF7 | 1.182982868 | 1.36573E-20 | 8.93066E-19 |
| CALD1 | 1.181633294 | 2.70936E-96 | 3.4902E-93 |
| TGFB2 | 1.178758832 | 5.83302E-66 | 2.783E-63 |
| CXCL8 | 1.174734736 | 3.22677E-72 | 1.66269E-69 |
| F3 | 1.167071525 | 2.87134E-86 | 3.08238E-83 |
| RGMB-AS1 | 1.164188564 | 0.00019445 | 0.001926845 |
| SH3BP5L | 1.159004276 | 3.51231E-51 | 8.87168E-49 |
| GPSM1 | 1.136580922 | 8.25053E-19 | 4.78754E-17 |
| BBC3 | 1.13396595 | 3.36599E-05 | 0.000415332 |
| SLC4A4 | 1.127242319 | 7.46765E-10 | 2.03379E-08 |
| MYO1E | 1.127143987 | 9.52279E-37 | 1.49601E-34 |
| DGKD | 1.123779871 | 4.9286E-52 | 1.2698E-49 |
| TMCC3 | 1.117939004 | 2.82129E-25 | 2.54152E-23 |
| NR4A1 | 1.10525393 | 0.001053472 | 0.008308088 |
| FST | 1.102451185 | 7.09807E-29 | 7.88253E-27 |
| MYPN | 1.091735947 | 0.007754758 | 0.042599912 |
| MIR210HG | 1.083845221 | 2.55115E-06 | 4.13903E-05 |
| NTN4 | 1.082035036 | 0.000593782 | 0.00510621 |
| C6orf132 | 1.081075447 | 4.06753E-31 | 5.13705E-29 |
| PTPRR | 1.080434884 | 8.88047E-05 | 0.000979437 |
| FAM131A | 1.079733798 | 1.81814E-16 | 9.04299E-15 |
| APBB1IP | 1.077147965 | 1.94382E-06 | 3.21441E-05 |
| GLIPR1 | 1.073694695 | 8.79993E-58 | 2.90668E-55 |
| CREBRF | 1.072460574 | 9.62214E-15 | 4.3189E-13 |
| JDP2 | 1.071341019 | 2.02776E-05 | 0.000265226 |
| TSSK3 | 1.061085634 | 0.000434834 | 0.003876497 |
| ARID3A | 1.060361134 | 0.000635385 | 0.005427735 |
| LOC100268168 | 1.057179457 | 0.005889686 | 0.034330741 |
| GADD45A | 1.055228235 | 2.41706E-36 | 3.66313E-34 |
| PERM1 | 1.054832243 | 0.005868953 | 0.034240876 |
| DAW1 | 1.048972744 | 0.001444889 | 0.010840451 |
| OSBP2 | 1.042873192 | 1.35489E-06 | 2.29956E-05 |
| CTGF | 1.042639889 | 1.00841E-83 | 9.27884E-81 |
| CHAC1 | 1.032336651 | 6.71805E-05 | 0.000771319 |
| IL6 | 1.027778073 | 7.53992E-74 | 4.6252E-71 |
| ABL2 | 1.022421023 | 7.11761E-82 | 6.1126E-79 |
| KLF6 | 1.020866217 | 7.22401E-78 | 4.89788E-75 |
| PPP2R5B | 1.018221718 | 7.32791E-22 | 5.39585E-20 |
| GJB3 | 1.015971757 | 0.000487697 | 0.004303091 |
| LIF | 1.007444518 | 1.70992E-73 | 9.57706E-71 |
| MAST4 | 1.005631604 | 5.76709E-22 | 4.31928E-20 |
| PSMB8-AS1 | -1.001871928 | 1.32202E-07 | 2.63627E-06 |
| TUBB | -1.015143518 | 2.7164E-138 | 8.7483E-135 |
| MAP3K8 | -1.017047541 | 7.22062E-07 | 1.27945E-05 |
| PAK6 | -1.017547157 | 8.31606E-05 | 0.000923513 |
| E2F2 | -1.018897788 | 9.603E-14 | 3.91474E-12 |
| CYP1B1 | -1.061374079 | 2.9215E-108 | 4.7044E-105 |
| VIPR1 | -1.078782897 | 1.26639E-19 | 7.88099E-18 |
| CYTH4 | -1.082215526 | 0.00139568 | 0.010507978 |
| NFIA | -1.083001497 | 4.62674E-08 | 9.87969E-07 |
| APOBEC3G | -1.093089694 | 0.006674966 | 0.037896389 |
| SPRY1 | -1.101481398 | 3.74136E-05 | 0.000456403 |
| BAHCC1 | -1.108491256 | 1.64144E-16 | 8.22766E-15 |
| ARMCX5-GPRASP2 | -1.114201018 | 0.003586607 | 0.023043724 |
| GPR68 | -1.114797397 | 3.9987E-41 | 7.57519E-39 |
| TTC30B | -1.134029035 | 7.74208E-05 | 0.000868758 |
| MTSS1 | -1.134867704 | 0.00117296 | 0.009107936 |
| C10orf55 | -1.138968031 | 0.000235764 | 0.002273285 |
| ITPKB | -1.155325709 | 0.000162906 | 0.001645986 |
| IL7 | -1.157177059 | 0.00454217 | 0.027823218 |
| WT1 | -1.169313144 | 1.2591E-12 | 4.68778E-11 |
| SHROOM2 | -1.169632838 | 0.002194482 | 0.015422428 |
| C1orf106 | -1.173357952 | 3.22303E-07 | 6.03475E-06 |
| FZD1 | -1.179248529 | 2.19103E-28 | 2.2762E-26 |
| CX3CL1 | -1.188682181 | 0.000279416 | 0.002627325 |
| TSC22D3 | -1.191663329 | 1.0674E-07 | 2.1688E-06 |
| ARRDC3 | -1.234759357 | 9.7485E-35 | 1.33596E-32 |
| CSF1 | -1.242219832 | 1.34664E-76 | 8.67369E-74 |
| PPM1H | -1.249515528 | 0.004738517 | 0.028835024 |
| BCL3 | -1.275910145 | 6.85825E-12 | 2.34345E-10 |
| IRF1 | -1.297841081 | 4.28496E-36 | 6.27259E-34 |
| VDR | -1.309109311 | 1.45518E-28 | 1.57526E-26 |
| BATF | -1.321811992 | 0.002540868 | 0.017327402 |
| CLDN2 | -1.347989765 | 9.42611E-12 | 3.17872E-10 |
| FAM46C | -1.356373973 | 7.88518E-11 | 2.40703E-09 |
| ZNF608 | -1.358247868 | 8.42045E-15 | 3.81945E-13 |
| PIK3R3 | -1.378168473 | 3.72083E-11 | 1.18058E-09 |
| ADAMTS15 | -1.437411678 | 5.22008E-51 | 1.29317E-48 |
| ST8SIA4 | -1.459856844 | 1.55597E-05 | 0.000210989 |
| IER5L | -1.471917981 | 2.78969E-05 | 0.000351288 |
| TMEM229B | -1.486427673 | 0.000781751 | 0.006488736 |
| CIITA | -1.500720121 | 4.58297E-21 | 3.10725E-19 |
| DACH1 | -1.504646082 | 0.003377177 | 0.022027744 |
| TRIB2 | -1.528860751 | 1.83087E-65 | 8.4233E-63 |
| C5orf56 | -1.584923605 | 0.00852973 | 0.045821511 |
| CYP1A1 | -1.737453253 | 1.45274E-25 | 1.34635E-23 |
| BMP4 | -1.765764748 | 1.6836E-101 | 2.40981E-98 |
| SLC16A9 | -1.78582724 | 0.000563102 | 0.004865108 |
| F2RL3 | -1.80369 | 1.33695E-05 | 0.000184199 |
| PARD6B | -1.844481084 | 7.61269E-40 | 1.36204E-37 |
| BMF | -1.96965722 | 5.26733E-06 | 7.88997E-05 |
| SMAD6 | -2.025022124 | 6.66064E-05 | 0.000765976 |
| PLEKHS1 | -2.382812119 | 2.77821E-09 | 7.14349E-08 |
| SLITRK5 | -2.457383282 | 0.000109522 | 0.001175718 |
| TNF | -2.591508249 | 1.84696E-05 | 0.000244988 |
| PDE4B | -2.917277973 | 2.43653E-17 | 1.29166E-15 |
| FOS | -4.09963998 | 7.4465E-109 | 1.3704E-105 |
| ZFP91-CNTF | -6.904638646 | 0.001533246 | 0.011389578 |

**Supplementary table S3. Differentially Expressed Genes (DEG’s) identified in Jurkat cells treated with P3C.1.**

| **DEG’s identified in Jurkat cells exposed to P3C.1** | | | |
| --- | --- | --- | --- |
| **Gene** | **log2FoldChange** | **pvalue** | **padj** |
| DUSP8 | 10.30221139 | 5.01592E-18 | 1.63049E-16 |
| EPPK1 | 7.325918239 | 1.18904E-08 | 1.38991E-07 |
| RGPD6 | 7.075250756 | 0.000282077 | 0.001416419 |
| CGA | 6.967173661 | 2.0111E-14 | 4.6847E-13 |
| CD69 | 6.919524301 | 8.9819E-112 | 3.6222E-109 |
| ACTG2 | 5.791714821 | 7.53005E-73 | 1.61959E-70 |
| GBP2 | 5.506227353 | 1.9144E-19 | 7.05867E-18 |
| SPRY4 | 5.208778704 | 8.61386E-15 | 2.07005E-13 |
| JUN | 4.992718235 | 6.3827E-304 | 2.7456E-300 |
| SIRPB2 | 4.949278077 | 1.41272E-16 | 4.05136E-15 |
| GLIPR1 | 4.153842613 | 7.2823E-27 | 4.0334E-25 |
| NEU4 | 3.624058268 | 1.10892E-12 | 2.17156E-11 |
| SYTL3 | 3.608783867 | 8.67634E-12 | 1.53171E-10 |
| NR4A3 | 3.492828586 | 2.06641E-54 | 3.1373E-52 |
| CD248 | 3.387801416 | 1.36117E-47 | 1.61155E-45 |
| CYSLTR2 | 3.235563392 | 8.89817E-06 | 6.31286E-05 |
| EVI2A | 3.234689063 | 3.6853E-17 | 1.0933E-15 |
| ZYX | 3.179013809 | 4.9766E-245 | 1.0704E-241 |
| GBP1 | 3.006598011 | 3.86002E-49 | 4.88369E-47 |
| PTPN14 | 3.003550466 | 3.36022E-83 | 8.84972E-81 |
| PDE4D | 2.960588958 | 4.0239E-222 | 5.7699E-219 |
| DUSP6 | 2.95234677 | 6.90859E-98 | 2.4096E-95 |
| DUSP10 | 2.846616455 | 7.8185E-121 | 3.4792E-118 |
| PRKCE | 2.846007472 | 6.0343E-136 | 3.5397E-133 |
| TPM4 | 2.815159711 | 0 | 0 |
| LONRF2 | 2.710427942 | 1.68177E-05 | 0.000112335 |
| ATF3 | 2.702153742 | 1.05605E-91 | 3.58641E-89 |
| ETV5 | 2.701183845 | 3.2932E-189 | 3.8635E-186 |
| JUND | 2.690124018 | 1.22268E-88 | 3.94468E-86 |
| TDRD9 | 2.674805021 | 1.40917E-06 | 1.17324E-05 |
| BMP10 | 2.659559549 | 9.95939E-19 | 3.49255E-17 |
| TGFBR3 | 2.647954033 | 5.03344E-05 | 0.000303111 |
| DUSP16 | 2.639465752 | 8.7252E-183 | 9.3832E-180 |
| PLAU | 2.581873724 | 1.32585E-35 | 9.94774E-34 |
| BBC3 | 2.573504633 | 2.27967E-11 | 3.82066E-10 |
| HELZ2 | 2.483106531 | 7.34667E-72 | 1.55424E-69 |
| HRH1 | 2.482413381 | 1.09834E-18 | 3.80004E-17 |
| BACH2 | 2.450426557 | 6.0433E-239 | 1.1141E-235 |
| ANGPTL2 | 2.386255165 | 3.7036E-270 | 9.5591E-267 |
| C3AR1 | 2.381294143 | 0.000345178 | 0.001688597 |
| TMEM71 | 2.353988504 | 5.67412E-10 | 7.95055E-09 |
| LIMA1 | 2.34955374 | 8.6453E-237 | 1.3946E-233 |
| GPR132 | 2.322249158 | 2.95377E-43 | 2.86605E-41 |
| RASSF8 | 2.294638723 | 1.3693E-171 | 1.2622E-168 |
| KRT2 | 2.288858674 | 2.34324E-05 | 0.000152417 |
| KLF7 | 2.286199631 | 1.14836E-50 | 1.57655E-48 |
| PPP1R16B | 2.260516973 | 0 | 0 |
| PRDM8 | 2.225433734 | 3.14087E-40 | 2.6843E-38 |
| CREM | 2.175033768 | 1.9841E-132 | 1.1132E-129 |
| RASSF2 | 2.174563907 | 1.2701E-292 | 4.0977E-289 |
| ABTB2 | 2.162494687 | 6.40651E-11 | 1.01693E-09 |
| CCNG2 | 2.15020932 | 3.1502E-104 | 1.1615E-101 |
| RTEL1-TNFRSF6B | 2.137550205 | 6.93229E-12 | 1.23395E-10 |
| RHOB | 2.096720466 | 6.47447E-10 | 9.01327E-09 |
| YPEL4 | 2.086217615 | 0.000773302 | 0.003451905 |
| BCHE | 2.082725933 | 8.32857E-81 | 2.1496E-78 |
| FLT1 | 2.065908963 | 2.28019E-17 | 7.00615E-16 |
| ANTXR2 | 2.050057518 | 5.3005E-117 | 2.2801E-114 |
| PPP1R15A | 2.038650724 | 8.10323E-60 | 1.3943E-57 |
| TP53INP1 | 2.038150314 | 6.69954E-16 | 1.80874E-14 |
| SERPINB8 | 2.03400442 | 2.59577E-24 | 1.23611E-22 |
| GLRX | 2.026677686 | 2.3449E-215 | 3.0261E-212 |
| ZNF93 | 2.009640707 | 2.0065E-111 | 7.8466E-109 |
| FERMT2 | 1.992174931 | 1.146E-110 | 4.3496E-108 |
| TP53INP2 | 1.980059995 | 2.03506E-59 | 3.41071E-57 |
| RELL1 | 1.967907976 | 2.30059E-47 | 2.65082E-45 |
| EVI2B | 1.953985849 | 8.58347E-22 | 3.66787E-20 |
| RAB30 | 1.953463496 | 1.55217E-73 | 3.51416E-71 |
| IL23A | 1.932606106 | 1.29504E-05 | 8.9086E-05 |
| TNF | 1.93207816 | 4.36716E-48 | 5.26712E-46 |
| GBP3 | 1.931749079 | 4.59805E-06 | 3.46397E-05 |
| ECM1 | 1.929156827 | 0.004399743 | 0.015568601 |
| KIAA1462 | 1.92596055 | 0.004531898 | 0.015940076 |
| MIR155HG | 1.911433041 | 4.30146E-10 | 6.12696E-09 |
| PALLD | 1.904458682 | 3.1406E-163 | 2.5331E-160 |
| MYH9 | 1.895948745 | 4.5524E-166 | 3.9166E-163 |
| SLC16A6 | 1.880919485 | 2.7702E-138 | 1.7024E-135 |
| RASSF8-AS1 | 1.867785358 | 5.26384E-05 | 0.000315953 |
| TDGF1 | 1.856949135 | 0.003058763 | 0.011346174 |
| TREML2 | 1.852802308 | 5.1545E-49 | 6.39603E-47 |
| NDRG4 | 1.844789085 | 1.3243E-40 | 1.14698E-38 |
| ENPP1 | 1.833118735 | 0.002939152 | 0.010971871 |
| LMNTD1 | 1.830076799 | 0.000263253 | 0.001333311 |
| CNN2 | 1.824577196 | 3.6879E-131 | 1.9037E-128 |
| SH2D2A | 1.823423962 | 3.21373E-35 | 2.35643E-33 |
| MIR4435-2HG | 1.79985007 | 2.76081E-35 | 2.0359E-33 |
| TRIB1 | 1.766402458 | 7.75771E-15 | 1.88893E-13 |
| SLC2A3 | 1.766293622 | 0.000411947 | 0.00197701 |
| VWA7 | 1.726623598 | 0.003164823 | 0.01169924 |
| CHRM3-AS2 | 1.70935997 | 2.96526E-05 | 0.000189158 |
| LUCAT1 | 1.707316803 | 4.77875E-05 | 0.000289122 |
| TRANK1 | 1.692419312 | 1.02043E-51 | 1.43137E-49 |
| LPP | 1.684554707 | 1.79769E-86 | 5.27256E-84 |
| CSRNP2 | 1.674869827 | 4.5167E-152 | 3.2382E-149 |
| PTPRE | 1.674036717 | 1.99751E-19 | 7.32876E-18 |
| VAC14-AS1 | 1.673576197 | 9.64877E-48 | 1.15294E-45 |
| KLF6 | 1.671791059 | 6.71174E-56 | 1.06932E-53 |
| LZTS3 | 1.654219378 | 3.58587E-10 | 5.15319E-09 |
| FOXB1 | 1.644481283 | 3.85948E-20 | 1.47794E-18 |
| MBNL1-AS1 | 1.633184979 | 0.005308091 | 0.018325553 |
| KIF21B | 1.632199329 | 3.6E-125 | 1.7206E-122 |
| KLF3 | 1.619306628 | 2.33522E-16 | 6.62329E-15 |
| ARHGEF4 | 1.618350176 | 0.007880014 | 0.02541654 |
| RNF125 | 1.61786082 | 1.44707E-45 | 1.54335E-43 |
| CABLES1 | 1.615826743 | 4.4783E-39 | 3.70465E-37 |
| MICALCL | 1.615596252 | 4.89201E-16 | 1.34322E-14 |
| PPP2R5B | 1.610293155 | 4.24149E-17 | 1.24969E-15 |
| B4GALT1 | 1.608258266 | 9.7418E-150 | 6.6168E-147 |
| LINC00152 | 1.600685549 | 2.96113E-21 | 1.20928E-19 |
| STX11 | 1.597692693 | 0.001048292 | 0.004491435 |
| ARHGAP29 | 1.593661238 | 8.82575E-06 | 6.26837E-05 |
| SH3RF2 | 1.593221395 | 0.000848528 | 0.003733991 |
| CRIM1 | 1.580548148 | 0.001298934 | 0.005412575 |
| WT1-AS | 1.579946102 | 0.003465829 | 0.012673993 |
| CSRNP1 | 1.57225623 | 7.16566E-30 | 4.53299E-28 |
| FBXO32 | 1.566406665 | 0.0020428 | 0.008005569 |
| DYRK1B | 1.564253406 | 4.09424E-11 | 6.65442E-10 |
| TRPS1 | 1.558845405 | 7.68564E-09 | 9.26474E-08 |
| PPP1R32 | 1.550897142 | 0.012300713 | 0.037097617 |
| DUSP1 | 1.548855755 | 4.88103E-35 | 3.53875E-33 |
| MCAM | 1.548158486 | 1.3055E-123 | 6.017E-121 |
| GPR3 | 1.545481878 | 4.0697E-11 | 6.62288E-10 |
| CDC42EP4 | 1.542376021 | 2.68358E-06 | 2.1104E-05 |
| STK17B | 1.524960163 | 8.49536E-87 | 2.5496E-84 |
| PLCL1 | 1.524917242 | 9.62592E-29 | 5.85955E-27 |
| PLCH1 | 1.520383508 | 2.2359E-113 | 9.308E-111 |
| CCR4 | 1.517733443 | 8.97625E-59 | 1.48511E-56 |
| CCRL2 | 1.517353979 | 2.3243E-33 | 1.64808E-31 |
| SLC14A1 | 1.516962842 | 3.42757E-11 | 5.65637E-10 |
| BMF | 1.511645309 | 5.74806E-05 | 0.000342311 |
| LBH | 1.504616139 | 1.8953E-29 | 1.18159E-27 |
| PDLIM7 | 1.497357092 | 6.01827E-12 | 1.07719E-10 |
| ULBP2 | 1.482987089 | 2.0523E-42 | 1.93321E-40 |
| DDAH2 | 1.47893477 | 3.04225E-27 | 1.72953E-25 |
| PTPRC | 1.478298517 | 3.4831E-148 | 2.2474E-145 |
| KRT77 | 1.478169147 | 0.015760649 | 0.045651016 |
| NABP1 | 1.473627017 | 5.71979E-44 | 5.59196E-42 |
| LOXL3 | 1.468492041 | 4.25667E-09 | 5.3126E-08 |
| EIF4E3 | 1.467320145 | 0.001165214 | 0.004925349 |
| LOC101927070 | 1.466617769 | 1.30632E-15 | 3.44747E-14 |
| IL21R | 1.46272348 | 2.25767E-08 | 2.5335E-07 |
| SLC14A2 | 1.458410583 | 8.16648E-15 | 1.97357E-13 |
| SRF | 1.456402867 | 3.48792E-75 | 8.18392E-73 |
| SLFN5 | 1.454252061 | 7.47262E-62 | 1.41815E-59 |
| KLHL24 | 1.446721839 | 3.31229E-42 | 3.07518E-40 |
| BSDC1 | 1.445778767 | 3.13931E-73 | 6.86657E-71 |
| SNPH | 1.445422657 | 1.34287E-26 | 7.3431E-25 |
| STYK1 | 1.43956142 | 9.88013E-07 | 8.48324E-06 |
| GPR65 | 1.431749007 | 8.38757E-22 | 3.59606E-20 |
| ARID5B | 1.426482869 | 6.1938E-47 | 7.07354E-45 |
| HERPUD1 | 1.425536464 | 2.43298E-83 | 6.68033E-81 |
| PMAIP1 | 1.406489939 | 2.21205E-84 | 6.20576E-82 |
| ARID5A | 1.405410563 | 1.85324E-31 | 1.23279E-29 |
| KCNJ10 | 1.405226288 | 0.000560927 | 0.002595468 |
| TMEM2 | 1.395848318 | 8.13459E-41 | 7.09303E-39 |
| EXT1 | 1.385610467 | 1.03477E-49 | 1.36638E-47 |
| SPAG4 | 1.382059499 | 0.000319586 | 0.001578359 |
| TM4SF19 | 1.378696372 | 0.001783256 | 0.007133239 |
| ITPRIP | 1.376302434 | 2.02825E-40 | 1.74497E-38 |
| MTMR11 | 1.374932912 | 0.001356993 | 0.00562544 |
| ITK | 1.371212447 | 2.5697E-132 | 1.3818E-129 |
| RAB43 | 1.360033672 | 4.73288E-10 | 6.71184E-09 |
| FAT1 | 1.359318563 | 1.472E-125 | 7.3062E-123 |
| ALOXE3 | 1.358503333 | 0.000463401 | 0.002191549 |
| PNRC1 | 1.351321391 | 7.02815E-06 | 5.10401E-05 |
| RHEBL1 | 1.350513415 | 4.42331E-05 | 0.000269372 |
| ACTB | 1.348661449 | 1.07219E-89 | 3.54784E-87 |
| TSSK3 | 1.347877892 | 1.86223E-08 | 2.11178E-07 |
| IQGAP3 | 1.342810775 | 5.17518E-36 | 3.92857E-34 |
| SLC16A13 | 1.340517244 | 1.54908E-05 | 0.000104445 |
| LCP1 | 1.330794798 | 3.3377E-156 | 2.5337E-153 |
| RELB | 1.324774538 | 2.6534E-08 | 2.94176E-07 |
| METRNL | 1.324084232 | 0.000465875 | 0.002202239 |
| CHRNA3 | 1.318814549 | 1.16432E-80 | 2.94618E-78 |
| CD93 | 1.313461891 | 3.60984E-05 | 0.000224182 |
| LINC01554 | 1.309398308 | 0.012414693 | 0.037406402 |
| LOC100130476 | 1.305145872 | 0.002105522 | 0.008216438 |
| BTG2 | 1.303026433 | 3.7459E-13 | 7.63679E-12 |
| VPS37B | 1.302504716 | 3.46245E-54 | 5.19569E-52 |
| TRAF1 | 1.295468495 | 8.52665E-07 | 7.39989E-06 |
| PTGES3L | 1.291182256 | 2.22632E-09 | 2.89624E-08 |
| STX12 | 1.288260317 | 3.83213E-49 | 4.88369E-47 |
| UBE2H | 1.284392949 | 2.09993E-61 | 3.81684E-59 |
| SLC7A11 | 1.284167611 | 4.8484E-49 | 6.07462E-47 |
| ST8SIA4 | 1.276890886 | 3.19015E-60 | 5.56336E-58 |
| PLAUR | 1.276723439 | 2.96102E-13 | 6.13353E-12 |
| CALCRL | 1.274879079 | 2.31184E-05 | 0.000150602 |
| RAB11FIP1 | 1.267344773 | 1.39991E-74 | 3.22604E-72 |
| ARMCX3 | 1.258260917 | 5.85842E-51 | 8.12935E-49 |
| MAFF | 1.255252697 | 6.38492E-08 | 6.64495E-07 |
| UBALD2 | 1.255168803 | 1.18586E-22 | 5.33222E-21 |
| LINC00963 | 1.254799849 | 0.000816397 | 0.003611796 |
| EIF2AK3 | 1.254534899 | 7.76907E-31 | 5.03818E-29 |
| NR4A2 | 1.251977187 | 4.53381E-05 | 0.000275465 |
| LIME1 | 1.250891453 | 0.000148452 | 0.000802849 |
| PRR4 | 1.248349627 | 0.007241654 | 0.023713154 |
| NDRG1 | 1.241424921 | 7.85944E-06 | 5.64733E-05 |
| C11orf96 | 1.240508595 | 0.000668331 | 0.003035837 |
| AKNA | 1.237872478 | 1.59191E-53 | 2.3345E-51 |
| RNF43 | 1.229243951 | 0.009414557 | 0.029625667 |
| STX1A | 1.228689703 | 1.22557E-07 | 1.22132E-06 |
| DEAF1 | 1.228563534 | 4.50544E-28 | 2.61904E-26 |
| TMOD1 | 1.22549289 | 0.001238223 | 0.005186389 |
| RBMS1 | 1.225400012 | 9.85937E-47 | 1.10639E-44 |
| PLEKHG2 | 1.218617865 | 1.58399E-46 | 1.76219E-44 |
| SORBS1 | 1.216009048 | 0.01493283 | 0.043758473 |
| LPIN1 | 1.214562152 | 2.02572E-75 | 4.84109E-73 |
| MCTP1 | 1.211528625 | 6.74309E-11 | 1.06511E-09 |
| SPI1 | 1.208245588 | 3.04498E-07 | 2.84544E-06 |
| FPR2 | 1.208113152 | 0.001450987 | 0.005963372 |
| RASGEF1B | 1.20326771 | 2.2398E-06 | 1.78776E-05 |
| EDEM1 | 1.202583416 | 1.88296E-78 | 4.67299E-76 |
| TES | 1.199655492 | 1.23198E-59 | 2.09194E-57 |
| GPR137C | 1.195877762 | 0.006734238 | 0.02227771 |
| ATXN1 | 1.195496424 | 4.3444E-25 | 2.13173E-23 |
| GAB2 | 1.194436287 | 2.03902E-48 | 2.4824E-46 |
| NPPA-AS1 | 1.193878083 | 0.009648715 | 0.030222491 |
| FOSL2 | 1.192755779 | 1.7192E-28 | 1.03192E-26 |
| ITPR1 | 1.192243649 | 1.27029E-61 | 2.3758E-59 |
| CORO1A | 1.18399106 | 1.45234E-08 | 1.67643E-07 |
| CLIC4 | 1.183768185 | 3.21553E-83 | 8.6451E-81 |
| MEF2D | 1.182009707 | 1.2488E-50 | 1.6964E-48 |
| ZBTB21 | 1.176390769 | 1.62118E-34 | 1.1623E-32 |
| LOC606724 | 1.16867299 | 0.00015579 | 0.000835608 |
| DAAM1 | 1.16859773 | 4.4306E-54 | 6.57206E-52 |
| RAET1E | 1.165274551 | 0.001145286 | 0.004847465 |
| TSC22D3 | 1.161654356 | 4.18619E-08 | 4.50941E-07 |
| BTN2A2 | 1.161131631 | 1.74856E-37 | 1.38437E-35 |
| RAPGEF2 | 1.158644244 | 1.38561E-49 | 1.8062E-47 |
| EMP3 | 1.155698561 | 4.41262E-27 | 2.46654E-25 |
| ARPC5 | 1.1556602 | 1.32738E-85 | 3.80664E-83 |
| BCL2 | 1.151788221 | 4.25126E-11 | 6.88611E-10 |
| HOMER1 | 1.150645653 | 1.37559E-41 | 1.25901E-39 |
| TLR9 | 1.150167622 | 6.29293E-22 | 2.72518E-20 |
| SPOCK2 | 1.148706808 | 9.61051E-47 | 1.08793E-44 |
| BRSK1 | 1.148009937 | 8.66037E-06 | 6.16108E-05 |
| IRGQ | 1.142268321 | 1.98567E-45 | 2.10042E-43 |
| PLEKHG1 | 1.139848076 | 3.19004E-44 | 3.19128E-42 |
| MTSS1L | 1.139474578 | 4.71861E-46 | 5.16048E-44 |
| SPRED2 | 1.139116592 | 1.01072E-56 | 1.63042E-54 |
| MACF1 | 1.137985612 | 1.85013E-63 | 3.61757E-61 |
| MXD1 | 1.136942695 | 4.02188E-17 | 1.1877E-15 |
| ACTG1 | 1.134296046 | 5.65177E-61 | 1.013E-58 |
| PNPLA8 | 1.133433876 | 4.88256E-30 | 3.10391E-28 |
| ADAMTS4 | 1.133103163 | 3.67185E-18 | 1.2119E-16 |
| MBNL2 | 1.131290464 | 9.81035E-10 | 1.33547E-08 |
| SRGAP2D | 1.121744807 | 0.005132245 | 0.017808988 |
| ACRC | 1.119907442 | 0.000536051 | 0.002488397 |
| SPATA2 | 1.119417297 | 6.34301E-36 | 4.78693E-34 |
| MICAL2 | 1.118231785 | 3.00465E-76 | 7.31604E-74 |
| MAST4 | 1.11787137 | 4.31488E-08 | 4.63643E-07 |
| FZD2 | 1.111866553 | 0.003528763 | 0.01286767 |
| GRK5 | 1.109557993 | 3.5441E-09 | 4.47083E-08 |
| MIR22HG | 1.108717339 | 2.01534E-07 | 1.93368E-06 |
| RPTOR | 1.107016267 | 2.29943E-32 | 1.58685E-30 |
| SLC43A2 | 1.105603217 | 5.07703E-31 | 3.30905E-29 |
| SNX33 | 1.105228332 | 0.000909124 | 0.003964949 |
| SPRY3 | 1.105044173 | 0.009806755 | 0.030620898 |
| ELL2 | 1.104087251 | 1.56415E-39 | 1.30228E-37 |
| PVRL2 | 1.102901289 | 2.71221E-13 | 5.64533E-12 |
| GEM | 1.102520252 | 2.87707E-08 | 3.16797E-07 |
| ARVCF | 1.102321834 | 0.006052912 | 0.020437685 |
| TRIM38 | 1.100899509 | 1.50439E-29 | 9.42432E-28 |
| SPTBN1 | 1.100468629 | 2.8198E-102 | 1.0108E-99 |
| ISG20 | 1.100373131 | 0.000421293 | 0.002015866 |
| PLAC8 | 1.098466314 | 2.05945E-09 | 2.69546E-08 |
| CACNB2 | 1.095900419 | 2.28581E-10 | 3.36356E-09 |
| ERP27 | 1.095026929 | 0.000472926 | 0.002228228 |
| MPZ | 1.090399229 | 3.57907E-11 | 5.87633E-10 |
| FLI1 | 1.090061836 | 1.4857E-44 | 1.5462E-42 |
| SLFN12 | 1.089964437 | 0.00013489 | 0.000737197 |
| TTC24 | 1.085731932 | 1.08011E-16 | 3.12528E-15 |
| GJC2 | 1.085131989 | 0.013663499 | 0.040581692 |
| POC1B-GALNT4 | 1.083179327 | 0.002027441 | 0.007961421 |
| S1PR1 | 1.082911683 | 9.79357E-08 | 9.93601E-07 |
| NLRP1 | 1.080789903 | 1.18779E-20 | 4.68761E-19 |
| BZRAP1-AS1 | 1.080172326 | 1.57548E-18 | 5.37871E-17 |
| C1orf186 | 1.079210709 | 2.03937E-13 | 4.29333E-12 |
| DNAJB5 | 1.075491472 | 6.81317E-21 | 2.72211E-19 |
| ACTN4 | 1.075448331 | 1.47871E-41 | 1.34386E-39 |
| SMPD1 | 1.072369929 | 0.00115651 | 0.004893366 |
| CCDC168 | 1.068809726 | 6.13972E-05 | 0.000363122 |
| DLGAP4 | 1.068154397 | 1.74696E-35 | 1.29567E-33 |
| MAPKBP1 | 1.067960069 | 1.88931E-37 | 1.48668E-35 |
| VASP | 1.067506788 | 7.46504E-41 | 6.55349E-39 |
| JMJD1C | 1.063347805 | 2.70249E-50 | 3.63288E-48 |
| FLNA | 1.061935642 | 1.70425E-37 | 1.35761E-35 |
| PTPN22 | 1.058783527 | 1.39963E-31 | 9.35867E-30 |
| ZNF821 | 1.058704634 | 5.43446E-06 | 4.03985E-05 |
| RAB27A | 1.055602283 | 8.79414E-26 | 4.59467E-24 |
| MZF1-AS1 | 1.052314644 | 0.015010233 | 0.043934465 |
| C22orf24 | 1.051054556 | 0.001018365 | 0.004379374 |
| DNAJC28 | 1.049975406 | 0.006683132 | 0.022148388 |
| TCP11L2 | 1.046490039 | 0.001474717 | 0.006047418 |
| FBXO48 | 1.046305891 | 9.73747E-07 | 8.37431E-06 |
| CARD8-AS1 | 1.043603722 | 4.72283E-06 | 3.54762E-05 |
| CTTN | 1.036225003 | 6.67481E-07 | 5.91204E-06 |
| MGAT4A | 1.034734536 | 4.65388E-52 | 6.59982E-50 |
| MUS81 | 1.031238381 | 1.50567E-24 | 7.25023E-23 |
| CCDC150 | 1.030792151 | 2.04688E-07 | 1.95957E-06 |
| YPEL1 | 1.0293498 | 4.10396E-15 | 1.03038E-13 |
| FBXO46 | 1.028232312 | 1.02163E-11 | 1.79377E-10 |
| MTCL1 | 1.026593053 | 1.00683E-18 | 3.52116E-17 |
| ARHGAP25 | 1.025437735 | 1.15321E-25 | 5.9768E-24 |
| PTCH1 | 1.01885455 | 2.91789E-25 | 1.44828E-23 |
| IRF7 | 1.017620753 | 0.000118984 | 0.000656754 |
| LRMP | 1.007506449 | 2.27371E-42 | 2.12625E-40 |
| CHPF2 | 1.005570126 | 7.14471E-37 | 5.55436E-35 |
| PBXIP1 | 1.005415609 | 1.89172E-30 | 1.22063E-28 |
| DOK2 | 1.004650995 | 1.7988E-15 | 4.69854E-14 |
| SRGN | 1.004438832 | 5.67446E-07 | 5.07126E-06 |
| TUBA1A | 1.002746526 | 1.11878E-39 | 9.49855E-38 |
| KLHL4 | 1.00051114 | 6.17731E-08 | 6.43927E-07 |
| ZFYVE21 | -1.000144754 | 8.77281E-27 | 4.83817E-25 |
| LOC100130238 | -1.001528789 | 6.41318E-07 | 5.70773E-06 |
| RGPD2 | -1.00206406 | 0.005824779 | 0.019802099 |
| ESPNL | -1.009453338 | 0.007399611 | 0.024101962 |
| TERT | -1.011133991 | 9.28989E-08 | 9.4622E-07 |
| CAMKV | -1.017385122 | 7.68457E-05 | 0.000445505 |
| SEPT5 | -1.018022874 | 5.49725E-09 | 6.74354E-08 |
| TNRC6C-AS1 | -1.01839152 | 9.4445E-07 | 8.14715E-06 |
| LYL1 | -1.02182153 | 0.00027659 | 0.00139375 |
| LINC00891 | -1.022586798 | 2.56203E-09 | 3.28985E-08 |
| LOC100132741 | -1.022586798 | 2.56203E-09 | 3.28985E-08 |
| TTYH2 | -1.024634595 | 1.38302E-05 | 9.43336E-05 |
| ZNF485 | -1.032855399 | 5.24808E-07 | 4.72003E-06 |
| CDHR1 | -1.032882814 | 0.00149665 | 0.006127622 |
| PTGR2 | -1.035394377 | 0.000485913 | 0.002279427 |
| DHODH | -1.035519097 | 2.14943E-23 | 9.94209E-22 |
| DCAF4 | -1.03830718 | 5.49427E-23 | 2.48784E-21 |
| GAS6-AS1 | -1.043626877 | 0.000468384 | 0.00221167 |
| CCDC116 | -1.047141359 | 0.000369232 | 0.001794029 |
| MRM1 | -1.047667498 | 7.89548E-15 | 1.917E-13 |
| HK2 | -1.051702769 | 1.09722E-32 | 7.65382E-31 |
| JAG2 | -1.059141397 | 3.28525E-05 | 0.000207519 |
| URB2 | -1.061978186 | 4.61177E-43 | 4.40851E-41 |
| POLR1B | -1.062785231 | 1.61101E-44 | 1.66321E-42 |
| NOP16 | -1.065850469 | 3.35055E-28 | 1.95651E-26 |
| DUSP7 | -1.068017937 | 1.98007E-30 | 1.27128E-28 |
| C10orf2 | -1.068516659 | 4.51134E-35 | 3.2892E-33 |
| GADD45B | -1.070264065 | 6.05716E-07 | 5.40578E-06 |
| LINC01550 | -1.070578011 | 3.74107E-05 | 0.000231662 |
| SLC29A2 | -1.07263172 | 1.29663E-23 | 6.0627E-22 |
| NOCT | -1.075163459 | 5.1789E-11 | 8.29202E-10 |
| FAM78B | -1.075575505 | 0.005677134 | 0.019376731 |
| FAM129C | -1.076689792 | 9.6582E-07 | 8.32036E-06 |
| TTC23 | -1.077613094 | 0.000198441 | 0.001037216 |
| GALNT9 | -1.077871837 | 3.57938E-15 | 9.0395E-14 |
| RRS1 | -1.08825223 | 2.97167E-26 | 1.58469E-24 |
| CHFR | -1.090224612 | 0.001344728 | 0.005579973 |
| MAT2A | -1.110039747 | 4.19918E-87 | 1.29025E-84 |
| SLC29A1 | -1.111836717 | 1.45036E-38 | 1.17716E-36 |
| TFAP4 | -1.112611253 | 1.49864E-17 | 4.68279E-16 |
| LOC102606465 | -1.125419674 | 0.007200549 | 0.023584539 |
| LRFN1 | -1.126042199 | 4.4533E-08 | 4.77324E-07 |
| SLC2A4 | -1.129655858 | 8.63112E-08 | 8.84005E-07 |
| MAP6D1 | -1.149105882 | 4.95867E-17 | 1.45767E-15 |
| C2orf88 | -1.173292272 | 5.20345E-05 | 0.000312619 |
| LINC01226 | -1.177808443 | 2.35016E-11 | 3.91845E-10 |
| CCDC78 | -1.185735298 | 1.43287E-23 | 6.67554E-22 |
| SNHG19 | -1.189412513 | 0.001533167 | 0.006241491 |
| SNHG12 | -1.194767262 | 1.91049E-11 | 3.26123E-10 |
| SLC29A4 | -1.200544271 | 3.49526E-05 | 0.000217905 |
| PPARGC1B | -1.219010893 | 3.80139E-44 | 3.77361E-42 |
| NRIP2 | -1.226682706 | 0.00802743 | 0.02582747 |
| MIR17HG | -1.232558119 | 1.38879E-05 | 9.4677E-05 |
| ASH1L-AS1 | -1.235790462 | 0.003676891 | 0.013302572 |
| ACKR3 | -1.236292527 | 0.003439957 | 0.012603552 |
| RAG1 | -1.243285238 | 3.04298E-88 | 9.57795E-86 |
| SNHG4 | -1.246834227 | 3.24909E-16 | 9.11511E-15 |
| STAB1 | -1.261024461 | 0.000387466 | 0.001868555 |
| RAB3A | -1.263702336 | 1.58049E-08 | 1.81461E-07 |
| MARS2 | -1.268949535 | 2.21872E-47 | 2.57952E-45 |
| LOC100996324 | -1.270891889 | 1.89552E-08 | 2.14576E-07 |
| ABCA3 | -1.27133752 | 0.0160512 | 0.046358921 |
| PMEPA1 | -1.273853877 | 7.08927E-16 | 1.90598E-14 |
| NR1D1 | -1.276561079 | 1.61418E-18 | 5.4963E-17 |
| BAHCC1 | -1.295657354 | 3.54273E-66 | 7.25698E-64 |
| HAGHL | -1.301201347 | 7.03986E-09 | 8.56262E-08 |
| OVGP1 | -1.307228645 | 6.12709E-06 | 4.52346E-05 |
| NR2F6 | -1.320672987 | 2.24802E-07 | 2.14417E-06 |
| CASKIN1 | -1.324604693 | 0.005947967 | 0.020153419 |
| CCDC26 | -1.351594572 | 3.97239E-10 | 5.68334E-09 |
| PKP1 | -1.356547086 | 2.78389E-21 | 1.14414E-19 |
| LHX9 | -1.362790937 | 0.004991858 | 0.017391612 |
| CBFA2T3 | -1.362890061 | 3.80553E-06 | 2.91976E-05 |
| LINC01260 | -1.368113238 | 0.004996226 | 0.017397813 |
| WNT8B | -1.383512436 | 0.00962126 | 0.030173113 |
| DLK1 | -1.386693406 | 0.000396422 | 0.001908179 |
| LRFN4 | -1.387819153 | 4.90166E-08 | 5.22345E-07 |
| LRRC38 | -1.391128062 | 2.93021E-06 | 2.28624E-05 |
| TINAGL1 | -1.428236808 | 0.009333785 | 0.029443288 |
| LINC00977 | -1.452331464 | 1.03762E-49 | 1.36638E-47 |
| KLF16 | -1.490978534 | 7.15192E-29 | 4.3742E-27 |
| FBXL16 | -1.496406651 | 4.54439E-07 | 4.12705E-06 |
| SLC16A9 | -1.507220503 | 8.36429E-07 | 7.26877E-06 |
| DDN | -1.513569315 | 2.30137E-49 | 2.96992E-47 |
| PWWP2B | -1.524857431 | 1.68799E-06 | 1.3866E-05 |
| LINC01225 | -1.525833797 | 1.11083E-06 | 9.41866E-06 |
| APOBEC2 | -1.529297648 | 0.002868775 | 0.01074022 |
| SSTR3 | -1.543324677 | 1.2756E-05 | 8.7969E-05 |
| EPB41L4A | -1.561926171 | 1.31635E-05 | 9.03111E-05 |
| ZNF296 | -1.643526536 | 1.31393E-05 | 9.01928E-05 |
| HPDL | -1.68107803 | 4.78592E-32 | 3.26784E-30 |
| KISS1R | -1.707030968 | 0.000689139 | 0.003113915 |
| SCO2 | -1.734491356 | 4.32167E-05 | 0.000264443 |
| CD3EAP | -1.745016061 | 2.59266E-41 | 2.33974E-39 |
| PLD6 | -1.750134668 | 1.22065E-71 | 2.54072E-69 |
| LINC01311 | -1.753168055 | 0.012490795 | 0.037600585 |
| TLCD1 | -1.774466212 | 1.89916E-09 | 2.49326E-08 |
| FAM163B | -1.833581245 | 9.90838E-08 | 1.00367E-06 |
| HLA-DOB | -1.840850379 | 0.000136109 | 0.000743013 |
| RPRML | -2.149817942 | 0.002504579 | 0.00955412 |
| MYC | -2.253845411 | 8.2347E-173 | 8.1745E-170 |
| OPRL1 | -2.469778813 | 8.12268E-55 | 1.24789E-52 |
| FJX1 | -2.62635574 | 9.94628E-07 | 8.52869E-06 |
| ZFP91-CNTF | -8.176252892 | 1.57198E-07 | 1.53918E-06 |

**Supplementary table S4. Differentially Expressed Genes (DEG’s) identified in Jurkat cells treated with P3C.2.**

| **DEG’s identified in Jurkat cells exposed to P3C.2** | | | |
| --- | --- | --- | --- |
| **Gene** | **log2FoldChange** | **pvalue** | **padj** |
| DUSP8 | 10.31476333 | 3.04786E-18 | 5.32497E-17 |
| EPPK1 | 7.329657218 | 8.09336E-09 | 6.2122E-08 |
| CGA | 7.255120506 | 6.50513E-17 | 1.04113E-15 |
| CD69 | 7.140533803 | 9.3194E-131 | 2.3036E-128 |
| ACTG2 | 6.05878486 | 2.40599E-84 | 3.41651E-82 |
| GBP2 | 5.850700597 | 2.22143E-23 | 5.50123E-22 |
| SIRPB2 | 5.397176575 | 6.68491E-22 | 1.5201E-20 |
| JUN | 5.162195473 | 0 | 0 |
| SPRY4 | 4.987215567 | 6.82156E-14 | 8.65534E-13 |
| KCNMB1 | 4.578131262 | 2.35806E-06 | 1.30333E-05 |
| C10orf32-ASMT | 4.473648526 | 0.008694492 | 0.023369731 |
| GLIPR1 | 4.366057837 | 7.95348E-34 | 3.19768E-32 |
| SYTL3 | 3.691777983 | 9.835E-14 | 1.2315E-12 |
| NR4A3 | 3.667259209 | 2.71386E-70 | 2.78651E-68 |
| EVI2A | 3.428123428 | 5.4529E-22 | 1.24846E-20 |
| NEU4 | 3.40194807 | 1.40162E-11 | 1.41949E-10 |
| CD248 | 3.383735197 | 2.4526E-103 | 4.3649E-101 |
| ZYX | 3.158056851 | 0 | 0 |
| PTPN14 | 3.130353844 | 4.1056E-109 | 8.059E-107 |
| GBP1 | 3.014930021 | 9.46385E-57 | 7.30194E-55 |
| DUSP6 | 2.998395111 | 4.3531E-117 | 9.5254E-115 |
| DUSP10 | 2.98811587 | 1.5996E-172 | 5.4746E-170 |
| PDE4D | 2.982314357 | 0 | 0 |
| CYSLTR2 | 2.967627177 | 3.95024E-05 | 0.000179285 |
| PRKCE | 2.950090691 | 2.2741E-191 | 1.0118E-188 |
| ATF3 | 2.886188269 | 3.4683E-144 | 9.8499E-142 |
| PLAU | 2.869383382 | 1.2486E-53 | 9.00881E-52 |
| C3AR1 | 2.857125208 | 9.0236E-07 | 5.27813E-06 |
| TPM4 | 2.827444541 | 0 | 0 |
| LONRF2 | 2.809394266 | 9.48081E-07 | 5.52861E-06 |
| ETV5 | 2.781207487 | 6.9105E-283 | 6.1494E-280 |
| DUSP16 | 2.773359231 | 1.9893E-258 | 1.4752E-255 |
| YPEL4 | 2.743289033 | 9.14036E-08 | 6.14019E-07 |
| BBC3 | 2.715362559 | 4.3549E-16 | 6.53871E-15 |
| HRH1 | 2.595824707 | 7.47215E-23 | 1.80685E-21 |
| RTEL1-TNFRSF6B | 2.582023217 | 1.38751E-29 | 4.66775E-28 |
| JUND | 2.567963603 | 7.3654E-109 | 1.4248E-106 |
| HELZ2 | 2.564935427 | 1.0959E-103 | 1.9768E-101 |
| ANGPTL2 | 2.510730321 | 0 | 0 |
| BMP10 | 2.505696184 | 9.45709E-20 | 1.8646E-18 |
| MGC16025 | 2.499375807 | 0.00193467 | 0.006173555 |
| TMEM71 | 2.479934518 | 5.29776E-13 | 6.20302E-12 |
| KLF7 | 2.467217124 | 4.72414E-73 | 5.43602E-71 |
| BACH2 | 2.445008016 | 0 | 0 |
| NBL1 | 2.380020692 | 8.53814E-07 | 5.01395E-06 |
| TSNAX-DISC1 | 2.362304411 | 6.44601E-06 | 3.3272E-05 |
| LIMA1 | 2.333068701 | 0 | 0 |
| TGFBR3 | 2.315665861 | 0.000441388 | 0.001615477 |
| GPR132 | 2.305016547 | 8.30105E-54 | 6.05478E-52 |
| RASSF8 | 2.301777517 | 6.0052E-268 | 5.0098E-265 |
| PPP1R16B | 2.295840557 | 0 | 0 |
| CREM | 2.292632955 | 1.4306E-201 | 6.5848E-199 |
| PRDM8 | 2.280326666 | 2.98888E-53 | 2.14492E-51 |
| PRL | 2.251497674 | 0.003389182 | 0.010169075 |
| CCNG2 | 2.249632645 | 8.2965E-188 | 3.2571E-185 |
| BCHE | 2.239411479 | 1.2023E-123 | 2.8657E-121 |
| ANTXR2 | 2.215864863 | 9.143E-189 | 3.6982E-186 |
| RASSF2 | 2.21253817 | 0 | 0 |
| SLC2A3 | 2.20978025 | 1.17013E-08 | 8.78949E-08 |
| KIAA0226L | 2.205613016 | 0.00200495 | 0.006359807 |
| ABTB2 | 2.203895852 | 1.14081E-12 | 1.29156E-11 |
| ECM1 | 2.183751424 | 0.000309988 | 0.001167857 |
| TMEM88 | 2.17484051 | 0.005065549 | 0.014522719 |
| TP53INP1 | 2.173747829 | 9.43566E-22 | 2.1239E-20 |
| GLRX | 2.155854969 | 0 | 0 |
| RASSF8-AS1 | 2.110551442 | 2.24543E-07 | 1.43201E-06 |
| FERMT2 | 2.095700374 | 1.5678E-206 | 7.751E-204 |
| KIAA1462 | 2.092786259 | 0.000597479 | 0.002135248 |
| SERPINB8 | 2.091071309 | 7.61281E-30 | 2.60553E-28 |
| SHC3 | 2.081617364 | 0.003899276 | 0.011525142 |
| EVI2B | 2.065438128 | 5.98324E-32 | 2.22463E-30 |
| PPP1R15A | 2.058614709 | 6.49821E-87 | 9.42806E-85 |
| FLT1 | 2.048455553 | 1.53651E-22 | 3.6364E-21 |
| OLFM2 | 2.030100029 | 0.006796962 | 0.018776045 |
| ZNF93 | 2.027584613 | 8.7248E-139 | 2.3767E-136 |
| RELL1 | 2.024847278 | 3.13091E-72 | 3.51188E-70 |
| RAB30 | 2.021199969 | 1.4727E-112 | 3.0243E-110 |
| COL1A1 | 2.006011539 | 0.00327438 | 0.009859334 |
| PALLD | 1.999905927 | 2.4627E-266 | 1.9337E-263 |
| TDRD9 | 1.999111649 | 0.000513256 | 0.001858637 |
| GBP3 | 1.987962661 | 2.53104E-07 | 1.60343E-06 |
| ABCB1 | 1.986498305 | 0.001954127 | 0.006226711 |
| IL23A | 1.979500038 | 9.79648E-07 | 5.70272E-06 |
| TP53INP2 | 1.975350859 | 3.05179E-70 | 3.10956E-68 |
| LOC101928100 | 1.968189664 | 0.002175026 | 0.006837027 |
| KRT2 | 1.966616946 | 0.000195563 | 0.000769568 |
| NDRG4 | 1.966213456 | 9.44023E-59 | 7.59086E-57 |
| P2RY10 | 1.933580369 | 0.002443592 | 0.007581839 |
| PPP2R5B | 1.90456294 | 9.6158E-33 | 3.68827E-31 |
| MIR4435-2HG | 1.891403523 | 2.46274E-49 | 1.58042E-47 |
| TNF | 1.889726685 | 8.43152E-54 | 6.11652E-52 |
| CSRNP1 | 1.886276808 | 1.31208E-55 | 9.83914E-54 |
| LMNTD1 | 1.883356604 | 4.82736E-05 | 0.000215937 |
| LUCAT1 | 1.872715968 | 2.18399E-07 | 1.39483E-06 |
| RHOB | 1.871296212 | 1.00487E-08 | 7.59941E-08 |
| MYH9 | 1.866111686 | 0 | 0 |
| L1CAM | 1.842769445 | 0.009041353 | 0.024180321 |
| KLK1 | 1.810137805 | 0.007240732 | 0.019862166 |
| SH2D2A | 1.806033657 | 2.74981E-39 | 1.36448E-37 |
| ENPP1 | 1.7972265 | 0.00182086 | 0.005841105 |
| TREML2 | 1.794021711 | 3.47613E-58 | 2.7784E-56 |
| SLC16A6 | 1.793453545 | 8.8948E-173 | 3.1244E-170 |
| GPR183 | 1.792728892 | 0.003642716 | 0.010833996 |
| KRCC1 | 1.790313624 | 0.011434616 | 0.029642505 |
| TRAF1 | 1.777830752 | 6.52062E-16 | 9.64935E-15 |
| ALG1L2 | 1.777664445 | 0.002206217 | 0.006920935 |
| TRIB1 | 1.770292922 | 1.36029E-17 | 2.25834E-16 |
| LOC101927070 | 1.758034643 | 4.00537E-30 | 1.38149E-28 |
| VAC14-AS1 | 1.749407584 | 8.11932E-82 | 1.08377E-79 |
| TRANK1 | 1.749027387 | 3.16539E-78 | 4.02396E-76 |
| TM4SF19 | 1.744648193 | 4.51106E-06 | 2.39203E-05 |
| ARHGAP29 | 1.731089054 | 4.93004E-08 | 3.42028E-07 |
| MBNL1-AS1 | 1.730748451 | 0.001073608 | 0.003633499 |
| SH3RF2 | 1.727955285 | 6.72475E-05 | 0.00029153 |
| CNN2 | 1.725664963 | 7.4286E-214 | 3.9663E-211 |
| LINC00152 | 1.721190936 | 5.94292E-31 | 2.10414E-29 |
| MIR155HG | 1.71427283 | 4.8367E-08 | 3.36251E-07 |
| VWA7 | 1.708741085 | 0.001761525 | 0.005669842 |
| TRPS1 | 1.695952429 | 4.85277E-12 | 5.16134E-11 |
| LBH | 1.688780329 | 1.1502E-50 | 7.63824E-49 |
| KLF6 | 1.687763551 | 2.41126E-71 | 2.6167E-69 |
| ITPKA | 1.667145316 | 0.008758791 | 0.02350942 |
| CABLES1 | 1.664720021 | 2.9172E-52 | 2.02806E-50 |
| LOXL3 | 1.663857988 | 2.49896E-15 | 3.57515E-14 |
| LPP | 1.652343784 | 1.1413E-146 | 3.3117E-144 |
| CCR4 | 1.649794768 | 1.10011E-93 | 1.74812E-91 |
| LZTS3 | 1.645301715 | 3.37242E-11 | 3.30022E-10 |
| NDRG1 | 1.644635345 | 3.05631E-12 | 3.31942E-11 |
| SLC14A2 | 1.644604586 | 3.60023E-19 | 6.79715E-18 |
| STK17B | 1.63857524 | 1.3569E-189 | 5.8427E-187 |
| CCRL2 | 1.637862359 | 2.96449E-61 | 2.50443E-59 |
| B4GALT1 | 1.63530904 | 3.1483E-219 | 1.751E-216 |
| PTPRE | 1.634813824 | 5.48692E-22 | 1.2541E-20 |
| CDC42EP4 | 1.627248317 | 4.60446E-08 | 3.21782E-07 |
| WT1-AS | 1.625146901 | 0.00116343 | 0.003906785 |
| ISG20 | 1.620679408 | 6.57664E-11 | 6.2436E-10 |
| KIF21B | 1.606574871 | 1.2554E-252 | 7.6166E-250 |
| MICALCL | 1.599791907 | 2.56874E-19 | 4.93345E-18 |
| KLHL24 | 1.59901431 | 8.59948E-75 | 1.03411E-72 |
| EIF4E3 | 1.598231829 | 8.61584E-05 | 0.000365209 |
| CSRNP2 | 1.597550752 | 1.4193E-227 | 8.2366E-225 |
| RNF125 | 1.596045728 | 7.41654E-73 | 8.46119E-71 |
| MTMR11 | 1.595293535 | 1.71883E-05 | 8.28265E-05 |
| TDGF1 | 1.591485144 | 0.009571426 | 0.025384341 |
| NABP1 | 1.59127651 | 9.79996E-83 | 1.33479E-80 |
| MCAM | 1.589033495 | 9.2659E-253 | 5.8896E-250 |
| KLF3 | 1.584379868 | 5.67472E-18 | 9.74854E-17 |
| ARHGEF4 | 1.580565318 | 0.006969259 | 0.019204308 |
| DDAH2 | 1.567185606 | 1.31876E-38 | 6.37782E-37 |
| PTPRC | 1.534419633 | 8.097E-285 | 7.7199E-282 |
| PLCH1 | 1.533280394 | 4.2084E-189 | 1.7554E-186 |
| PNRC1 | 1.531148083 | 9.56318E-33 | 3.67865E-31 |
| PMAIP1 | 1.530982438 | 8.8313E-152 | 2.7414E-149 |
| ARID5A | 1.520504412 | 4.80297E-51 | 3.23788E-49 |
| CALCRL | 1.514830188 | 5.47328E-09 | 4.28992E-08 |
| DUSP1 | 1.511650179 | 2.86118E-44 | 1.6321E-42 |
| LOC100130476 | 1.511076349 | 3.86132E-05 | 0.000175649 |
| GPR3 | 1.510150118 | 6.00997E-13 | 6.99399E-12 |
| SLFN5 | 1.496096113 | 2.74569E-94 | 4.4156E-92 |
| METRNL | 1.483558306 | 8.38176E-06 | 4.24752E-05 |
| RAB43 | 1.46973894 | 0.000241918 | 0.000932464 |
| EXT1 | 1.464764033 | 2.56926E-76 | 3.2051E-74 |
| BSDC1 | 1.462063217 | 4.3105E-120 | 9.752E-118 |
| BTG2 | 1.461506086 | 5.66704E-21 | 1.22401E-19 |
| BMF | 1.461217087 | 2.93818E-05 | 0.000136841 |
| DYRK1B | 1.459543586 | 1.88361E-13 | 2.29611E-12 |
| ULBP2 | 1.459101402 | 7.35237E-57 | 5.73915E-55 |
| GPR65 | 1.454718854 | 4.79297E-32 | 1.78706E-30 |
| APOBR | 1.453841269 | 0.014055798 | 0.035533482 |
| SNPH | 1.439286463 | 4.31044E-32 | 1.61165E-30 |
| PDLIM7 | 1.432070464 | 7.52133E-14 | 9.51608E-13 |
| CHRM3-AS2 | 1.423425168 | 0.000273993 | 0.001044931 |
| SLC14A1 | 1.417422367 | 1.84372E-12 | 2.04402E-11 |
| RHEBL1 | 1.414263415 | 2.35611E-06 | 1.30279E-05 |
| ARID5B | 1.411215715 | 4.72741E-61 | 3.94384E-59 |
| LCP1 | 1.410190351 | 0 | 0 |
| FPR2 | 1.409009661 | 6.87235E-06 | 3.52952E-05 |
| ITPRIP | 1.405301044 | 2.39675E-60 | 1.96269E-58 |
| TMEM2 | 1.404284036 | 9.49675E-52 | 6.56801E-50 |
| LINC00963 | 1.399159523 | 3.04077E-05 | 0.000141324 |
| UBE2H | 1.397046461 | 3.896E-123 | 9.1234E-121 |
| FOXB1 | 1.392776323 | 2.2959E-17 | 3.76482E-16 |
| PLCL1 | 1.392226878 | 1.92083E-27 | 5.89407E-26 |
| ARMCX3 | 1.392087383 | 3.51645E-93 | 5.52207E-91 |
| FBXO32 | 1.391967409 | 0.003437379 | 0.010294399 |
| SRF | 1.384506624 | 1.86E-184 | 7.0937E-182 |
| LPAR6 | 1.379459239 | 0.003277951 | 0.009867858 |
| EIF2AK3 | 1.379053061 | 3.22252E-60 | 2.62281E-58 |
| CD93 | 1.378740107 | 1.09753E-06 | 6.33915E-06 |
| HERPUD1 | 1.371321274 | 1.20685E-90 | 1.85161E-88 |
| RNF43 | 1.36446325 | 0.001592893 | 0.005178259 |
| GUSBP9 | 1.363500001 | 5.9074E-05 | 0.000259467 |
| IQGAP3 | 1.36201464 | 2.05272E-60 | 1.69134E-58 |
| MXD1 | 1.356000315 | 3.79523E-29 | 1.24469E-27 |
| ITK | 1.351412769 | 1.101E-207 | 5.6523E-205 |
| FAT1 | 1.351114537 | 3.9389E-255 | 2.6288E-252 |
| GPER1 | 1.350275825 | 0.006585293 | 0.01824829 |
| MCTP1 | 1.347022493 | 3.77137E-20 | 7.67382E-19 |
| ST8SIA4 | 1.344183675 | 3.7138E-99 | 6.27499E-97 |
| PTGES3L | 1.342922627 | 6.24796E-12 | 6.59792E-11 |
| ALOXE3 | 1.337480475 | 9.40561E-05 | 0.00039567 |
| GJC2 | 1.335001029 | 0.0006216 | 0.002215519 |
| TSSK3 | 1.334055559 | 1.53051E-09 | 1.26811E-08 |
| RAB11FIP1 | 1.333770318 | 1.1118E-132 | 2.8539E-130 |
| FOSL2 | 1.331839283 | 8.85637E-47 | 5.47291E-45 |
| STYK1 | 1.329328514 | 1.97454E-06 | 1.10047E-05 |
| LINC01554 | 1.31959777 | 0.005232506 | 0.014942982 |
| C11orf96 | 1.305975023 | 7.63884E-05 | 0.000327099 |
| ACTB | 1.298776861 | 3.1442E-170 | 1.0236E-167 |
| EMP3 | 1.298084896 | 5.00878E-44 | 2.83293E-42 |
| BTN2A2 | 1.296319043 | 1.14888E-63 | 1.02921E-61 |
| IL21R | 1.295053037 | 3.2697E-07 | 2.0423E-06 |
| KLF9 | 1.294383723 | 0.020795381 | 0.049950826 |
| STX12 | 1.294131996 | 1.41398E-66 | 1.33857E-64 |
| EDEM1 | 1.287383495 | 8.3071E-167 | 2.6401E-164 |
| CHRNA3 | 1.284599486 | 8.3204E-108 | 1.5866E-105 |
| ZBTB21 | 1.283885172 | 1.67668E-64 | 1.51219E-62 |
| SRGAP2D | 1.280029894 | 5.50946E-05 | 0.000243419 |
| CCDC168 | 1.2772254 | 2.01631E-08 | 1.46989E-07 |
| CLIC4 | 1.275580985 | 3.4214E-178 | 1.2686E-175 |
| RBMS1 | 1.275561894 | 4.11912E-71 | 4.36365E-69 |
| LINC01096 | 1.272767556 | 0.016061292 | 0.039826515 |
| VPS37B | 1.26772437 | 4.19538E-70 | 4.24242E-68 |
| SPAG4 | 1.257014834 | 0.000284128 | 0.001079879 |
| PCDHGC3 | 1.250874789 | 0.020269448 | 0.048881046 |
| MAFF | 1.250130028 | 6.10898E-09 | 4.76022E-08 |
| MCF2L | 1.247206998 | 0.000213947 | 0.000835263 |
| TES | 1.23976081 | 1.38675E-89 | 2.10345E-87 |
| DEAF1 | 1.239464909 | 1.84594E-36 | 8.18592E-35 |
| GEM | 1.234215402 | 7.85523E-12 | 8.22366E-11 |
| RAPGEF2 | 1.232676174 | 1.4557E-75 | 1.79914E-73 |
| LPIN1 | 1.231056355 | 4.6383E-117 | 9.9858E-115 |
| RASGEF1B | 1.227566184 | 1.11672E-07 | 7.39382E-07 |
| PVRL2 | 1.226127048 | 1.37177E-19 | 2.67696E-18 |
| PLAUR | 1.225688826 | 3.52091E-15 | 4.94187E-14 |
| IRGQ | 1.223707274 | 8.90937E-72 | 9.82828E-70 |
| DNAJB5 | 1.219601969 | 1.11694E-35 | 4.74807E-34 |
| SLC7A11 | 1.218873249 | 3.67747E-79 | 4.81244E-77 |
| KCNN4 | 1.218415659 | 0.000206496 | 0.000808425 |
| UBALD2 | 1.21776809 | 2.94358E-42 | 1.56538E-40 |
| GRK5 | 1.215076403 | 4.32853E-13 | 5.11303E-12 |
| PNPLA8 | 1.212662005 | 1.26254E-45 | 7.66019E-44 |
| C1orf186 | 1.212453476 | 4.46489E-20 | 9.01624E-19 |
| ARPC5 | 1.211301288 | 1.8277E-151 | 5.5445E-149 |
| SPOCK2 | 1.208440164 | 4.04572E-74 | 4.73704E-72 |
| MACF1 | 1.208101427 | 1.8395E-170 | 6.1385E-168 |
| ACTG1 | 1.207706064 | 2.3638E-127 | 5.7367E-125 |
| CORO1A | 1.205129558 | 5.78906E-18 | 9.91944E-17 |
| ITPR1 | 1.203388576 | 9.10161E-79 | 1.16816E-76 |
| LIME1 | 1.201988886 | 8.94501E-18 | 1.49823E-16 |
| AKNA | 1.201798457 | 6.7122E-121 | 1.5447E-118 |
| ATXN1 | 1.200623704 | 2.68043E-33 | 1.05541E-31 |
| CPPED1 | 1.194345387 | 1.60561E-16 | 2.48917E-15 |
| PLEKHG2 | 1.193835235 | 1.58893E-56 | 1.21195E-54 |
| NR4A2 | 1.193676727 | 2.01065E-05 | 9.58848E-05 |
| RAB27A | 1.193617096 | 7.37461E-45 | 4.29853E-43 |
| HOMER1 | 1.192757896 | 1.28413E-83 | 1.78548E-81 |
| PLAC8 | 1.192390533 | 1.96611E-15 | 2.84022E-14 |
| TLR6 | 1.190631757 | 0.004003115 | 0.011787685 |
| ARVCF | 1.186982745 | 0.001439061 | 0.004731178 |
| MPZ | 1.185108248 | 1.65795E-15 | 2.40025E-14 |
| PTPN22 | 1.180507362 | 1.73456E-57 | 1.36193E-55 |
| F3 | 1.172184977 | 0.003335141 | 0.010030975 |
| PLEKHG1 | 1.165783867 | 3.0542E-67 | 2.95416E-65 |
| DAAM1 | 1.162285108 | 2.36727E-74 | 2.79631E-72 |
| MBNL2 | 1.161701159 | 3.96079E-12 | 4.24307E-11 |
| MEF2D | 1.153608472 | 2.14072E-70 | 2.21506E-68 |
| ELL2 | 1.14272365 | 2.86746E-55 | 2.13826E-53 |
| RPTOR | 1.14199925 | 1.9299E-21 | 4.24388E-20 |
| FKBP9P1 | 1.141149257 | 0.00262446 | 0.008090366 |
| CARD8-AS1 | 1.135061715 | 2.82743E-08 | 2.03015E-07 |
| TTC24 | 1.128859121 | 2.68878E-22 | 6.2526E-21 |
| GPR85 | 1.128627834 | 1.07275E-35 | 4.57478E-34 |
| ANXA3 | 1.128277336 | 9.46584E-37 | 4.32705E-35 |
| RELB | 1.127549473 | 1.61704E-06 | 9.11114E-06 |
| TSC22D3 | 1.123925778 | 1.84152E-08 | 1.34984E-07 |
| CTTN | 1.121385553 | 2.21063E-09 | 1.79923E-08 |
| ACRC | 1.11721069 | 0.000149849 | 0.000605018 |
| GPR174 | 1.116090756 | 5.8241E-18 | 9.96668E-17 |
| SPRED2 | 1.114197125 | 3.98102E-79 | 5.15909E-77 |
| SPATA2 | 1.113639354 | 1.1402E-48 | 7.28202E-47 |
| SCARF1 | 1.111434165 | 6.20367E-07 | 3.71997E-06 |
| BRSK1 | 1.109954037 | 1.75331E-06 | 9.82914E-06 |
| GPR137C | 1.108703134 | 0.004889819 | 0.014060599 |
| TRIM38 | 1.107779089 | 2.22248E-36 | 9.82306E-35 |
| SPTBN1 | 1.105569753 | 6.4057E-202 | 3.0537E-199 |
| TMOD1 | 1.103263529 | 0.002412944 | 0.00749576 |
| PCAT18 | 1.100884027 | 1.17816E-05 | 5.83747E-05 |
| FLI1 | 1.100176148 | 4.60664E-82 | 6.21105E-80 |
| HIST2H2BE | 1.099087069 | 0.014844936 | 0.037253282 |
| RAET1E | 1.09561631 | 0.000971375 | 0.003322888 |
| MYL12A | 1.095502038 | 3.577E-137 | 9.5491E-135 |
| JMJD1C | 1.095154667 | 2.06603E-96 | 3.3631E-94 |
| PTCH1 | 1.092634749 | 5.75526E-39 | 2.82431E-37 |
| HLA-DMB | 1.089380707 | 0.018028197 | 0.044097558 |
| MGAT4A | 1.083752428 | 1.49152E-98 | 2.48861E-96 |
| RORA | 1.083378944 | 0.000531314 | 0.001915717 |
| CACNB2 | 1.082396582 | 5.12114E-11 | 4.92485E-10 |
| C22orf24 | 1.08027352 | 0.000166281 | 0.000664329 |
| SLC43A2 | 1.079983876 | 1.07543E-46 | 6.61515E-45 |
| NLRP1 | 1.079435808 | 3.65918E-29 | 1.20302E-27 |
| FZD2 | 1.079017026 | 0.001577411 | 0.00513293 |
| GAB2 | 1.07523711 | 7.13188E-54 | 5.23057E-52 |
| KLHL4 | 1.074255869 | 8.2633E-12 | 8.63056E-11 |
| MICAL2 | 1.073819703 | 5.5886E-112 | 1.1134E-109 |
| LETM2 | 1.073209099 | 1.96395E-10 | 1.78697E-09 |
| ARMCX2 | 1.072312296 | 0.000820805 | 0.002857618 |
| HECA | 1.071044334 | 3.36466E-56 | 2.55179E-54 |
| ARHGAP25 | 1.070606494 | 1.76137E-36 | 7.86313E-35 |
| RGCC | 1.06839818 | 1.19889E-07 | 7.90654E-07 |
| ATP2C1 | 1.066088267 | 2.3043E-101 | 4.047E-99 |
| SLC16A13 | 1.06572897 | 0.000304496 | 0.00115009 |
| KCNJ10 | 1.064136445 | 0.007658868 | 0.020867641 |
| TLR9 | 1.061208857 | 3.62277E-29 | 1.19399E-27 |
| PTGES3L-AARSD1 | 1.060075099 | 0.002380757 | 0.007414452 |
| FBXO48 | 1.057856985 | 1.31288E-08 | 9.80108E-08 |
| LOC606724 | 1.056248547 | 0.000298039 | 0.001127936 |
| DLGAP4 | 1.055904467 | 2.48893E-40 | 1.2632E-38 |
| TFPI | 1.052075019 | 8.67117E-24 | 2.21305E-22 |
| PBXIP1 | 1.046695078 | 1.72503E-45 | 1.03719E-43 |
| ID1 | 1.04641177 | 0.007125665 | 0.019594845 |
| ZNF250 | 1.043513954 | 7.36087E-28 | 2.28495E-26 |
| RGS12 | 1.040704666 | 2.27442E-06 | 1.26075E-05 |
| ADAMTS4 | 1.038634114 | 5.35481E-19 | 9.95487E-18 |
| MTSS1L | 1.036753839 | 3.94379E-89 | 5.91479E-87 |
| PLXNB3 | 1.028547032 | 6.56884E-05 | 0.0002852 |
| ZSCAN16 | 1.0285071 | 0.000763389 | 0.002679389 |
| BTN1A1 | 1.027966745 | 0.006191213 | 0.017306873 |
| ERP27 | 1.027185572 | 0.000399874 | 0.00147649 |
| RORB | 1.025613642 | 3.11274E-42 | 1.64876E-40 |
| CDKN1B | 1.025559938 | 4.08657E-36 | 1.77679E-34 |
| SLFN12 | 1.021077898 | 0.000173003 | 0.000689531 |
| ACTN4 | 1.019831128 | 2.20401E-61 | 1.87383E-59 |
| FLNA | 1.019827829 | 2.29261E-26 | 6.65256E-25 |
| LGALS1 | 1.018433475 | 8.65445E-36 | 3.70255E-34 |
| LRMP | 1.017667107 | 2.55567E-67 | 2.50831E-65 |
| APLF | 1.016311386 | 0.001081496 | 0.003656488 |
| FAM107B | 1.012104143 | 5.24255E-74 | 6.08501E-72 |
| LINC00540 | 1.011734367 | 4.35282E-07 | 2.66092E-06 |
| INHBE | 1.011595204 | 1.23024E-20 | 2.5983E-19 |
| PHLDA1 | 1.011129728 | 0.000230828 | 0.000894627 |
| SPI1 | 1.010787768 | 6.46699E-07 | 3.86053E-06 |
| MIR22HG | 1.010742669 | 2.11378E-07 | 1.35323E-06 |
| UBE2B | 1.006203573 | 1.09773E-46 | 6.72133E-45 |
| MAPKBP1 | 1.004942469 | 1.27178E-56 | 9.75615E-55 |
| PLP2 | 1.004433214 | 1.11537E-30 | 3.90761E-29 |
| ARG2 | 1.002425725 | 4.9241E-35 | 2.0604E-33 |
| STX1A | 1.001980599 | 6.76647E-07 | 4.02491E-06 |
| TUBA1A | 1.001291095 | 2.04985E-52 | 1.44007E-50 |
| SMIM14 | 1.00039144 | 9.81446E-10 | 8.29135E-09 |
| GALNT3 | 1.000079497 | 0.01758229 | 0.043154659 |
| PUS7 | -1.001781587 | 3.4211E-51 | 2.32984E-49 |
| UNC93B1 | -1.004012786 | 1.14318E-15 | 1.67499E-14 |
| FSD2 | -1.006563775 | 0.002888806 | 0.008817697 |
| RNASEH1-AS1 | -1.00664384 | 1.60375E-05 | 7.77018E-05 |
| VPS9D1-AS1 | -1.010341459 | 4.88175E-13 | 5.74618E-12 |
| PRR5 | -1.013379727 | 9.21143E-08 | 6.1786E-07 |
| RXRA | -1.024523212 | 4.43909E-10 | 3.89566E-09 |
| LOC100996324 | -1.024986436 | 1.77615E-07 | 1.14587E-06 |
| FAM155B | -1.029607593 | 1.36067E-08 | 1.01352E-07 |
| RPUSD1 | -1.030977364 | 5.28297E-22 | 1.21372E-20 |
| AMER1 | -1.035567261 | 1.01054E-42 | 5.57385E-41 |
| TTYH2 | -1.036279647 | 1.6585E-06 | 9.32504E-06 |
| ZFYVE21 | -1.037082574 | 1.00123E-36 | 4.54571E-35 |
| DCAF4 | -1.038456121 | 1.5081E-33 | 5.99109E-32 |
| FAM195A | -1.046729384 | 1.20842E-12 | 1.36233E-11 |
| PUS1 | -1.04772752 | 2.51032E-37 | 1.16752E-35 |
| B4GALT2 | -1.049557797 | 3.80046E-17 | 6.14147E-16 |
| ANKRD13B | -1.050548943 | 6.40472E-24 | 1.65358E-22 |
| LINC01226 | -1.05554791 | 8.60254E-11 | 8.09208E-10 |
| FAM173A | -1.056945924 | 0.000102356 | 0.000427219 |
| SNHG12 | -1.059029954 | 9.46139E-12 | 9.76725E-11 |
| MAFG-AS1 | -1.060164382 | 5.82007E-05 | 0.000255968 |
| JAG2 | -1.061135062 | 1.28597E-19 | 2.5132E-18 |
| CCDC85B | -1.063301816 | 6.85815E-07 | 4.07581E-06 |
| GTPBP3 | -1.063515062 | 2.67582E-36 | 1.17489E-34 |
| NEU3 | -1.063551606 | 1.35212E-42 | 7.30692E-41 |
| FYB | -1.066838678 | 0.001983286 | 0.006298572 |
| GRASP | -1.072041916 | 3.05527E-13 | 3.66413E-12 |
| CLEC18A | -1.075621256 | 0.008276443 | 0.022381272 |
| TRMT61A | -1.075694311 | 8.96851E-27 | 2.64264E-25 |
| LINC00957 | -1.075774962 | 9.53956E-05 | 0.0004008 |
| ATP6AP1L | -1.075963258 | 0.006536958 | 0.01813286 |
| CCDC26 | -1.076003072 | 6.31283E-09 | 4.90476E-08 |
| MAN1B1-AS1 | -1.076829684 | 0.003104264 | 0.009395855 |
| CTRL | -1.07903384 | 0.002346157 | 0.00731865 |
| WNT8B | -1.081534437 | 0.013840804 | 0.035076333 |
| DUSP7 | -1.081595633 | 1.9616E-43 | 1.10015E-41 |
| IDI2-AS1 | -1.082050268 | 0.017520856 | 0.043030061 |
| MRM1 | -1.08358531 | 1.53014E-19 | 2.97296E-18 |
| PYCRL | -1.084404888 | 2.02848E-10 | 1.84066E-09 |
| TMEM229B | -1.084858918 | 3.74184E-12 | 4.02791E-11 |
| RABL2A | -1.086908279 | 0.000828721 | 0.002879919 |
| DHODH | -1.091341575 | 2.92602E-34 | 1.19805E-32 |
| BAMBI | -1.095120596 | 6.10165E-09 | 4.75729E-08 |
| TGFB3 | -1.095578822 | 2.22111E-24 | 5.92948E-23 |
| NOP16 | -1.104161442 | 1.82972E-50 | 1.20311E-48 |
| HAGHL | -1.105871583 | 1.71506E-11 | 1.72125E-10 |
| AACSP1 | -1.110707469 | 5.48525E-07 | 3.3085E-06 |
| LINC01550 | -1.110973758 | 4.94704E-06 | 2.60273E-05 |
| LTBP3 | -1.115493554 | 1.65079E-05 | 7.97207E-05 |
| ESPNL | -1.116215414 | 0.001323735 | 0.004393141 |
| DBP | -1.118828967 | 6.26026E-09 | 4.86958E-08 |
| LHX9 | -1.122831302 | 0.01061667 | 0.027764755 |
| HSPA1B | -1.124904413 | 5.93704E-38 | 2.8202E-36 |
| LINC00891 | -1.126007101 | 3.26067E-13 | 3.89646E-12 |
| LOC100132741 | -1.126007101 | 3.26067E-13 | 3.89646E-12 |
| SSTR3 | -1.126062364 | 0.00018921 | 0.000746993 |
| ZBTB42 | -1.129053374 | 2.76158E-14 | 3.61034E-13 |
| LOC728485 | -1.129546826 | 2.91209E-08 | 2.08533E-07 |
| TUBB | -1.134404308 | 1.1213E-256 | 7.8777E-254 |
| FAM78B | -1.134539302 | 0.002431617 | 0.007546581 |
| NOCT | -1.144209956 | 1.74225E-14 | 2.3186E-13 |
| C10orf2 | -1.151304273 | 1.21746E-61 | 1.04843E-59 |
| MRVI1 | -1.153101815 | 0.005840105 | 0.016452874 |
| NRARP | -1.154294025 | 0.002935393 | 0.008947618 |
| URB2 | -1.154593861 | 2.78719E-71 | 2.97628E-69 |
| NRXN2 | -1.157526265 | 0.00241906 | 0.007510958 |
| OVGP1 | -1.16681482 | 4.8768E-06 | 2.5699E-05 |
| ADGRB2 | -1.16723078 | 0.007937482 | 0.021556359 |
| RHPN1-AS1 | -1.170189134 | 2.16265E-09 | 1.76341E-08 |
| POLR1B | -1.174091745 | 1.80712E-75 | 2.21297E-73 |
| CRACR2B | -1.177361171 | 1.76556E-06 | 9.89368E-06 |
| TERT | -1.183172737 | 2.10589E-11 | 2.09147E-10 |
| SLC29A1 | -1.189275957 | 7.00866E-72 | 7.79597E-70 |
| MAT2A | -1.194883341 | 5.3251E-177 | 1.9211E-174 |
| GPSM1 | -1.202321596 | 0.002406555 | 0.007482576 |
| LRFN1 | -1.205358899 | 3.60111E-12 | 3.88268E-11 |
| SLC29A2 | -1.208416898 | 5.26669E-45 | 3.09691E-43 |
| MAP6D1 | -1.208449289 | 1.41227E-23 | 3.5501E-22 |
| RRS1 | -1.209744934 | 3.48513E-53 | 2.48767E-51 |
| STAB1 | -1.21102238 | 0.000132349 | 0.000541736 |
| NOTCH1 | -1.213189163 | 9.82988E-58 | 7.76386E-56 |
| SEPT5 | -1.230181209 | 2.0399E-14 | 2.68791E-13 |
| GALNT9 | -1.232950857 | 6.51067E-25 | 1.79184E-23 |
| CASKIN1 | -1.250223682 | 0.002090566 | 0.006598456 |
| FBXL16 | -1.257823968 | 2.81974E-06 | 1.53374E-05 |
| CCDC78 | -1.258743516 | 7.58543E-36 | 3.25564E-34 |
| PTPRVP | -1.263906889 | 0.004604704 | 0.013341346 |
| SNHG4 | -1.269601826 | 1.274E-23 | 3.22072E-22 |
| TFAP4 | -1.27078884 | 1.07197E-31 | 3.93093E-30 |
| RAG1 | -1.314237985 | 4.308E-149 | 1.2778E-146 |
| MARS2 | -1.315875798 | 8.16048E-66 | 7.51214E-64 |
| ASH1L-AS1 | -1.316155999 | 0.000731189 | 0.002579257 |
| PWWP2B | -1.32357664 | 1.697E-06 | 9.52547E-06 |
| CCDC116 | -1.333813412 | 2.79007E-07 | 1.75669E-06 |
| LYL1 | -1.335270459 | 1.36972E-06 | 7.79992E-06 |
| LINC01225 | -1.341461634 | 1.04803E-06 | 6.07959E-06 |
| CBFA2T3 | -1.354209111 | 3.73388E-07 | 2.30847E-06 |
| PMEPA1 | -1.371055119 | 1.23419E-20 | 2.60252E-19 |
| PPARGC1B | -1.371503843 | 2.24217E-71 | 2.45315E-69 |
| BAHCC1 | -1.378606245 | 1.2038E-141 | 3.3474E-139 |
| PKP1 | -1.419390698 | 1.118E-31 | 4.08852E-30 |
| FAM163B | -1.437278133 | 1.56682E-06 | 8.85059E-06 |
| SNHG19 | -1.438109925 | 7.21108E-05 | 0.000311198 |
| KLF16 | -1.463024979 | 7.23E-42 | 3.78455E-40 |
| DDN | -1.480802739 | 1.31823E-66 | 1.25684E-64 |
| GAS6-AS1 | -1.487755493 | 8.4163E-07 | 4.95112E-06 |
| LINC00977 | -1.521662146 | 1.46622E-66 | 1.37825E-64 |
| NR1D1 | -1.526340259 | 2.61382E-29 | 8.65738E-28 |
| LINC01260 | -1.570997586 | 0.000527095 | 0.001904103 |
| RAB3A | -1.574059733 | 8.2739E-16 | 1.22033E-14 |
| SLC16A9 | -1.586003897 | 1.31875E-08 | 9.83391E-08 |
| ACKR3 | -1.604821656 | 0.000104113 | 0.000434282 |
| NAT8L | -1.61256791 | 0.004821289 | 0.013907288 |
| LRFN4 | -1.632153073 | 2.87071E-18 | 5.02205E-17 |
| KISS1R | -1.634754433 | 0.000278215 | 0.001059215 |
| DLK1 | -1.655084796 | 9.43927E-06 | 4.74559E-05 |
| EPB41L4A | -1.656318074 | 9.35983E-07 | 5.46522E-06 |
| CAND2 | -1.757015159 | 0.000990734 | 0.003384775 |
| HPDL | -1.759247951 | 9.80486E-45 | 5.69023E-43 |
| FJX1 | -1.806533638 | 2.46128E-05 | 0.000115966 |
| LINC01311 | -1.811224749 | 0.003946302 | 0.011641904 |
| MIR17HG | -1.812009709 | 1.15591E-12 | 1.30644E-11 |
| SCO2 | -1.818968411 | 4.57068E-07 | 2.78455E-06 |
| TLCD1 | -1.87319895 | 1.35996E-11 | 1.37834E-10 |
| PLD6 | -1.924156803 | 2.6359E-116 | 5.5848E-114 |
| RPRML | -2.079145474 | 0.001192926 | 0.00399678 |
| CD3EAP | -2.111269259 | 2.82897E-63 | 2.50073E-61 |
| PGBD5 | -2.136164793 | 0.004647924 | 0.01345197 |
| ZNF296 | -2.138183527 | 2.31893E-08 | 1.67767E-07 |
| LINC01604 | -2.152342722 | 0.006726931 | 0.018601839 |
| FAM46B | -2.309648532 | 0.001699461 | 0.005489935 |
| EVPLL | -2.487839095 | 0.000896052 | 0.003088175 |
| MYC | -2.497308647 | 0 | 0 |
| OPRL1 | -2.500754007 | 7.78229E-71 | 8.11547E-69 |
| HSD11B2 | -3.793637158 | 0.000270849 | 0.001034716 |

**Supplementary table S5. Differentially Expressed Genes (DEG’s) identified in CEM cells treated with P3C.1.**

| **DEG’s identified in CEM cells exposed to P3C.1** | | | |
| --- | --- | --- | --- |
| **Gene** | **log2FoldChange** | **pvalue** | **padj** |
| RHOB | 5.183175644 | 4.28529E-08 | 5.28846E-06 |
| GBP2 | 5.147491824 | 8.22727E-06 | 0.000507662 |
| NEU4 | 4.588979174 | 9.86306E-06 | 0.000597222 |
| ACTG2 | 4.377869999 | 8.68672E-15 | 3.04223E-12 |
| SYTL3 | 4.342022205 | 3.66909E-07 | 3.66364E-05 |
| NPAS4 | 4.283535898 | 1.5185E-09 | 2.38756E-07 |
| STAT4 | 4.044482856 | 6.3763E-07 | 5.90172E-05 |
| CD248 | 3.650558793 | 4.14078E-57 | 1.78854E-53 |
| NR4A3 | 3.612312615 | 9.52277E-10 | 1.62363E-07 |
| LGALS12 | 3.392861343 | 2.98585E-15 | 1.07474E-12 |
| MYRF | 3.392790918 | 1.03443E-57 | 6.7021E-54 |
| DUSP8 | 2.858145856 | 4.02168E-19 | 1.93011E-16 |
| GLIPR1 | 2.755151873 | 5.21765E-29 | 4.50735E-26 |
| CABLES1 | 2.559244789 | 7.26434E-79 | 9.41314E-75 |
| TGFBR3 | 2.462888895 | 6.13741E-09 | 8.73941E-07 |
| RGS1 | 2.358194335 | 1.09326E-21 | 5.90269E-19 |
| B3GNT7 | 2.345240401 | 4.33883E-05 | 0.002170758 |
| FHL2 | 2.293461179 | 1.80711E-20 | 9.36661E-18 |
| GBP1 | 2.229576886 | 1.19453E-39 | 1.71985E-36 |
| LINC00473 | 2.191906037 | 7.82262E-10 | 1.35154E-07 |
| BCL3 | 2.150247019 | 2.26081E-10 | 4.30817E-08 |
| LINC00602 | 2.087529914 | 3.13772E-05 | 0.001646098 |
| JUN | 2.059806361 | 5.13619E-30 | 5.11959E-27 |
| LRP4 | 1.973524563 | 1.87941E-44 | 4.05889E-41 |
| PLAU | 1.956848601 | 0.002033621 | 0.044152523 |
| BMP10 | 1.942872232 | 0.002414071 | 0.049653235 |
| DMBT1 | 1.912242176 | 9.2818E-08 | 1.0934E-05 |
| DNAH10 | 1.907910575 | 7.30593E-06 | 0.000461806 |
| RASSF2 | 1.841170409 | 5.09417E-55 | 1.65025E-51 |
| RTP5 | 1.733578133 | 1.42053E-06 | 0.00011433 |
| GFRA4 | 1.704226574 | 0.000234686 | 0.008308926 |
| ZYX | 1.695410285 | 2.60586E-26 | 1.87593E-23 |
| PDE4D | 1.650813954 | 1.73415E-13 | 5.22584E-11 |
| FMN1 | 1.646709555 | 2.46131E-32 | 2.89942E-29 |
| TSC22D3 | 1.566948596 | 2.65786E-09 | 4.00471E-07 |
| RASSF8-AS1 | 1.549357483 | 2.48828E-06 | 0.0001832 |
| RAB30 | 1.519777706 | 2.26205E-09 | 3.44842E-07 |
| GAB2 | 1.513661145 | 4.63523E-31 | 5.00527E-28 |
| FERMT2 | 1.498173044 | 6.13557E-13 | 1.72836E-10 |
| KLF7 | 1.482015674 | 4.15677E-13 | 1.22417E-10 |
| SH3PXD2A | 1.458818446 | 9.67077E-05 | 0.004108652 |
| ARHGAP29 | 1.454758013 | 2.09599E-12 | 5.43196E-10 |
| CNN2 | 1.447241796 | 1.19326E-24 | 7.73114E-22 |
| KRT72 | 1.391029977 | 1.57004E-10 | 3.0365E-08 |
| BACH2 | 1.389741583 | 1.00144E-47 | 2.59534E-44 |
| MCAM | 1.344856663 | 1.70006E-24 | 1.00133E-21 |
| LIMA1 | 1.343539632 | 6.58681E-27 | 5.0207E-24 |
| KLF6 | 1.33548878 | 5.907E-28 | 4.78394E-25 |
| MAP1B | 1.331444593 | 0.000936138 | 0.024605418 |
| ADAMTS6 | 1.326901425 | 7.61466E-05 | 0.003379138 |
| RASSF8 | 1.324745004 | 1.1906E-12 | 3.21413E-10 |
| PPP1R16B | 1.305238442 | 3.07452E-41 | 5.69138E-38 |
| CD69 | 1.293126008 | 1.91018E-15 | 7.50064E-13 |
| PTGES3L | 1.266466924 | 2.5113E-06 | 0.00018385 |
| KIAA1549 | 1.266163263 | 4.25132E-07 | 4.142E-05 |
| ABCA4 | 1.265991676 | 0.0005583 | 0.016404659 |
| GPR132 | 1.25742585 | 0.000361104 | 0.011668802 |
| PRKCE | 1.253855773 | 4.1797E-22 | 2.35481E-19 |
| KRT73 | 1.24459857 | 1.43627E-07 | 1.60174E-05 |
| CCNJL | 1.232835322 | 1.36459E-06 | 0.00011121 |
| CDKN1A | 1.223555941 | 0.000231551 | 0.008265669 |
| LOC101927153 | 1.203034419 | 2.59711E-05 | 0.001390635 |
| COTL1 | 1.201470074 | 1.33709E-17 | 5.9745E-15 |
| PPP2R5B | 1.18288163 | 1.69197E-07 | 1.8424E-05 |
| TNF | 1.172232041 | 1.02673E-14 | 3.50116E-12 |
| SPTB | 1.171790635 | 1.10283E-07 | 1.27593E-05 |
| TPM4 | 1.162785875 | 1.82138E-36 | 2.36014E-33 |
| NABP1 | 1.137879639 | 2.22002E-07 | 2.31992E-05 |
| TRANK1 | 1.134867824 | 2.09375E-11 | 4.59845E-09 |
| SNPH | 1.123498129 | 1.26341E-06 | 0.000104276 |
| MYH15 | 1.120479594 | 0.000877219 | 0.023210604 |
| RBM38 | 1.110383728 | 5.24662E-09 | 7.55397E-07 |
| GVINP1 | 1.099692754 | 2.72506E-10 | 5.11758E-08 |
| LCP1 | 1.089116067 | 1.50919E-29 | 1.39686E-26 |
| GNAO1 | 1.088567514 | 0.001359407 | 0.033015188 |
| CHRNA3 | 1.087245558 | 1.2756E-24 | 7.87103E-22 |
| LZTS3 | 1.085698193 | 5.83219E-05 | 0.002736232 |
| FLT1 | 1.079062847 | 1.45061E-09 | 2.32062E-07 |
| MACF1 | 1.063686163 | 3.7639E-40 | 6.09658E-37 |
| FOXB1 | 1.049779246 | 4.04938E-06 | 0.000277629 |
| FOSL2 | 1.048956544 | 1.15942E-14 | 3.85224E-12 |
| MICAL2 | 1.048944159 | 7.77189E-26 | 5.30043E-23 |
| P2RY10 | 1.038781628 | 0.000258931 | 0.009019417 |
| DUSP10 | 1.037367163 | 1.88657E-06 | 0.000147103 |
| SYT11 | 1.024379895 | 0.000147883 | 0.005720937 |
| SH2D2A | 1.023704583 | 9.85668E-07 | 8.572E-05 |
| RELB | 1.023636081 | 0.001597091 | 0.037088001 |
| LOC441081 | -1.019211175 | 2.00852E-07 | 2.11597E-05 |
| HPDL | -1.034753899 | 5.05706E-05 | 0.002460989 |
| POLR3G | -1.049749027 | 5.14856E-05 | 0.002480115 |
| LINC00957 | -1.089841977 | 0.000494334 | 0.014759387 |
| DERL3 | -1.101188025 | 2.48859E-05 | 0.001343629 |
| MYC | -1.118264878 | 2.05353E-17 | 8.86989E-15 |
| RPL17-C18orf32 | -1.266387309 | 0.000521556 | 0.015465268 |
| SP2-AS1 | -1.279101756 | 0.00222465 | 0.046873192 |
| LOC100289580 | -1.292088156 | 2.58832E-06 | 0.000188424 |
| DDN | -1.303033978 | 0.001229053 | 0.030451377 |
| LOC339059 | -1.392165145 | 0.002282203 | 0.047532198 |
| RGPD8 | -1.578231275 | 1.32429E-08 | 1.82555E-06 |
| ZNF750 | -1.913135101 | 0.001550905 | 0.036275494 |
| GMCL1P1 | -1.947169867 | 0.002097468 | 0.044998333 |
| CCDC39 | -1.957342617 | 0.001330377 | 0.032649674 |
| NBL1 | -3.026086746 | 0.000710442 | 0.019622343 |

**Supplementary table S6. Differentially Expressed Genes (DEG’s) identified in CEM cells treated with P3C.2.**

| **DEG’s identified in CEM cells exposed to P3C.2** | | | |
| --- | --- | --- | --- |
| **Gene** | **log2FoldChange** | **pvalue** | **padj** |
| RHOB | 4.905895557 | 1.51816E-07 | 1.17017E-05 |
| NEU4 | 4.892596666 | 1.59381E-07 | 1.22113E-05 |
| STAT4 | 4.609899224 | 3.66833E-10 | 4.55692E-08 |
| ACTG2 | 4.441247653 | 6.0628E-16 | 1.84699E-13 |
| SYTL3 | 4.333022964 | 1.72502E-07 | 1.31379E-05 |
| NPAS4 | 4.062344232 | 1.0964E-08 | 1.11337E-06 |
| NR4A3 | 4.055150836 | 2.28334E-13 | 5.51233E-11 |
| LGALS12 | 3.559543156 | 1.45004E-19 | 5.62222E-17 |
| MYRF | 3.406805807 | 2.30833E-69 | 1.47675E-65 |
| CD248 | 3.399842274 | 1.08912E-54 | 3.48383E-51 |
| B3GNT7 | 3.006950867 | 1.2277E-08 | 1.23689E-06 |
| TGFBR3 | 2.690468819 | 2.68344E-13 | 6.35826E-11 |
| GLIPR1 | 2.628036455 | 6.94902E-31 | 5.23016E-28 |
| CABLES1 | 2.51139106 | 1.71985E-83 | 2.20054E-79 |
| RGS1 | 2.503084254 | 3.95852E-33 | 3.37662E-30 |
| LINC00473 | 2.305241485 | 2.77795E-11 | 4.73198E-09 |
| FHL2 | 2.21812839 | 3.30902E-27 | 2.11695E-24 |
| GBP1 | 2.207286646 | 4.0017E-45 | 7.31454E-42 |
| DUSP8 | 2.199602761 | 1.55139E-10 | 2.18133E-08 |
| FLNC | 2.190602762 | 0.000105425 | 0.003485571 |
| LINC00602 | 2.117329005 | 2.70902E-06 | 0.000153371 |
| TSC22D3 | 2.071373546 | 1.71731E-20 | 7.32431E-18 |
| LRP4 | 2.03164236 | 9.46035E-57 | 4.03484E-53 |
| DNAH10 | 1.907962785 | 1.35644E-06 | 8.42504E-05 |
| PDE10A | 1.903786638 | 0.001888477 | 0.031380604 |
| LOC63930 | 1.888202318 | 8.76572E-19 | 3.11548E-16 |
| EFCAB12 | 1.877442282 | 7.73654E-05 | 0.002734505 |
| RTP5 | 1.83978178 | 2.28387E-09 | 2.60912E-07 |
| PDE4D | 1.837964705 | 2.00549E-20 | 8.27751E-18 |
| SYCP2L | 1.799908266 | 0.000208987 | 0.005942209 |
| FMN1 | 1.790434703 | 3.99459E-46 | 8.51847E-43 |
| LOC100130357 | 1.768571649 | 0.001252932 | 0.023066565 |
| DMBT1 | 1.723681524 | 7.83147E-08 | 6.50673E-06 |
| BCL3 | 1.718294898 | 1.11395E-07 | 8.85276E-06 |
| JUN | 1.686541684 | 4.36481E-35 | 3.98912E-32 |
| RASSF2 | 1.685368128 | 1.69123E-48 | 4.32786E-45 |
| ARHGAP29 | 1.637705791 | 6.37751E-18 | 2.14737E-15 |
| KLF3 | 1.584839528 | 0.000498417 | 0.011679932 |
| DRP2 | 1.54504607 | 1.52412E-13 | 3.82375E-11 |
| GAB2 | 1.521992811 | 2.49072E-36 | 2.45145E-33 |
| ZYX | 1.501438678 | 7.83291E-25 | 4.00888E-22 |
| ABCA4 | 1.499884279 | 1.59484E-05 | 0.00072106 |
| KRT72 | 1.463498856 | 3.02096E-15 | 8.22407E-13 |
| PTGES3L-AARSD1 | 1.431688783 | 0.001020346 | 0.019543909 |
| RASSF8 | 1.431158934 | 2.27885E-19 | 8.57583E-17 |
| ARHGEF4 | 1.426250142 | 0.002480867 | 0.038336584 |
| KLF7 | 1.421321259 | 2.3648E-14 | 6.17502E-12 |
| APOLD1 | 1.418948795 | 1.17035E-09 | 1.36133E-07 |
| KLF6 | 1.406128074 | 4.85111E-40 | 5.64272E-37 |
| RAB30 | 1.385857809 | 2.98769E-09 | 3.32355E-07 |
| NABP1 | 1.379002554 | 4.01176E-12 | 8.02039E-10 |
| PRKCE | 1.339339502 | 8.86096E-29 | 6.29867E-26 |
| CNN2 | 1.330306027 | 4.51802E-26 | 2.75277E-23 |
| RASL10A | 1.325345762 | 0.001044791 | 0.019922656 |
| FERMT2 | 1.31962784 | 3.26825E-13 | 7.49394E-11 |
| SH3PXD2A | 1.301907583 | 0.000594586 | 0.013464996 |
| BACH2 | 1.293367532 | 2.32545E-44 | 3.71927E-41 |
| C1S | 1.292778057 | 0.000588847 | 0.013382414 |
| RASSF8-AS1 | 1.278557053 | 0.000183955 | 0.00539839 |
| LIMA1 | 1.269859983 | 8.99678E-24 | 4.42745E-21 |
| MICALCL | 1.267623585 | 0.000160392 | 0.004897891 |
| CD55 | 1.227332884 | 6.58518E-06 | 0.00033703 |
| MYLK4 | 1.223772222 | 0.001005784 | 0.019346218 |
| KIAA1549 | 1.213711591 | 2.40361E-07 | 1.76748E-05 |
| FKBP1A-SDCBP2 | 1.212689545 | 2.34434E-07 | 1.73386E-05 |
| KRT73 | 1.208894537 | 2.34094E-08 | 2.1863E-06 |
| PPP1R16B | 1.196091815 | 4.19893E-40 | 5.37253E-37 |
| LOC101927153 | 1.176832794 | 9.15929E-06 | 0.000443914 |
| COTL1 | 1.175734441 | 7.94618E-21 | 3.63112E-18 |
| SH2D2A | 1.171597933 | 3.61802E-11 | 5.93494E-09 |
| GVINP1 | 1.165486457 | 8.74447E-12 | 1.59836E-09 |
| CD69 | 1.161453881 | 5.22337E-12 | 9.97507E-10 |
| TPM4 | 1.14988834 | 4.39456E-37 | 4.6857E-34 |
| MCAM | 1.127638652 | 2.5122E-19 | 9.18388E-17 |
| CELF5 | 1.116875291 | 0.000136983 | 0.004274866 |
| RELB | 1.11021156 | 0.000148003 | 0.004563143 |
| FLT1 | 1.101068556 | 2.07286E-10 | 2.76273E-08 |
| GPR132 | 1.077197477 | 0.001449017 | 0.025432341 |
| LCP1 | 1.07585554 | 3.94815E-32 | 3.15729E-29 |
| RBM38 | 1.072100149 | 4.57039E-11 | 7.13148E-09 |
| CHRNA3 | 1.0666628 | 2.69882E-27 | 1.81744E-24 |
| ADAMTS6 | 1.066512023 | 0.000736152 | 0.01564628 |
| CTNND1 | 1.065027989 | 0.000194813 | 0.005677979 |
| RNF125 | 1.064478732 | 1.22449E-15 | 3.48164E-13 |
| CCDC146 | 1.048091603 | 0.000546187 | 0.012637359 |
| COLQ | 1.046769795 | 0.003477084 | 0.048889335 |
| TRANK1 | 1.037413102 | 5.85003E-11 | 8.91085E-09 |
| PKI55 | 1.036637665 | 4.82332E-11 | 7.43546E-09 |
| MICAL2 | 1.030817766 | 9.80453E-26 | 5.70223E-23 |
| NPTXR | 1.016012749 | 0.000358056 | 0.008921723 |
| SLC2A3 | 1.014903296 | 2.95323E-15 | 8.21447E-13 |
| NDST3 | 1.00876436 | 2.75361E-08 | 2.5166E-06 |
| CCNJL | 1.005885097 | 3.61525E-05 | 0.001477862 |
| MACF1 | 1.00458831 | 2.87254E-44 | 4.08379E-41 |
| ACVR2B-AS1 | -1.039746328 | 0.000294098 | 0.007679547 |
| MRM1 | -1.0478384 | 6.50184E-06 | 0.000334101 |
| FAM86B3P | -1.056778175 | 0.003056816 | 0.04449598 |
| APLN | -1.073266161 | 4.68154E-07 | 3.29123E-05 |
| LOC100289580 | -1.07681517 | 4.8627E-06 | 0.000262977 |
| MYC | -1.089363776 | 9.23716E-21 | 4.0755E-18 |
| FAM222A | -1.090381091 | 0.000856174 | 0.017360931 |
| TEX22 | -1.096150556 | 0.002696333 | 0.040876285 |
| ASH1L-AS1 | -1.117989888 | 0.000240974 | 0.006630667 |
| HPDL | -1.133635949 | 5.89709E-07 | 3.99223E-05 |
| DDN | -1.377345325 | 0.000312107 | 0.008083836 |
| LINC01311 | -1.437674107 | 0.002923829 | 0.043049928 |
| LOC100272216 | -1.633409848 | 0.000101795 | 0.003400704 |
| LOC339059 | -1.650815237 | 5.01897E-05 | 0.001916946 |
| LOC100507091 | -1.795433796 | 0.000667413 | 0.014547782 |
| ZNF750 | -2.045164775 | 4.8483E-05 | 0.001857306 |
